# Observation of an oxonium ion intermediate in ethanol dehydration to ethene on zeolite

**DOI:** 10.1038/s41467-019-09956-7

**Published:** 2019-04-29

**Authors:** Xue Zhou, Chao Wang, Yueying Chu, Jun Xu, Qiang Wang, Guodong Qi, Xingling Zhao, Ningdong Feng, Feng Deng

**Affiliations:** 1 National Centre for Magnetic Resonance in Wuhan, State Key Laboratory of Magnetic Resonance and Atomic and Molecular Physics, Key Laboratory of Magnetic Resonance in Biological Systems, Wuhan Institute of Physics and Mathematics, Chinese Academy of Sciences, Wuhan, 430071 China; 2 University of Chinese Academy of Sciences, Beijing, 100049 China; 3 Wuhan National Laboratory for Optoelectronics, Huazhong University of Science and Technology, Wuhan, 430074 China

**Keywords:** Catalytic mechanisms, Heterogeneous catalysis, Reaction mechanisms

## Abstract

Zeolite-catalyzed dehydration of ethanol offers promising perspectives for the sustainable production of ethene. Complex parallel-consecutive pathways are proposed to be involved in the reaction network of ethanol dehydration on zeolites, where the initial step of ethanol dehydration is still unclear particularly for the favorable production of ethene at lower temperature. Here we report the observation of a triethyloxonium ion (TEO) in the dehydration of ethanol on zeolite H-ZSM-5 by using ex situ and in situ solid-state NMR spectroscopy. TEO is identified as a stable surface species on the working catalyst, which shows high reactivity during reaction. Ethylation of the zeolite by TEO occurs at lower temperature, leading to the formation of surface ethoxy species and then ethene. The TEO-ethoxide pathway is found to be energetically preferable for the dehydration of ethanol to ethene in the initial stage, which is also supported by theoretical calculations.

## Abstract (web summary)

Zeolite-catalyzed dehydration of ethanol offers promising perspectives for the sustainable production of ethene, while the initial step of ethanol dehydration is still unclear. Here, the authors report the observation of a triethyloxonium ion intermediate in ethanol dehydration to ethene on H-ZSM-5 by using solid-state NMR spectroscopy.

## Introduction

Ethene is one of the most important commodity chemicals, which is currently produced by cracking processes from petroleum. Due to the large availability of bioethanol from renewable biomass sources and the dwindling of fossil resource^1^, the conversion of ethanol to ethene and higher hydrocarbons now is receiving increasing attention from both academia and industry^2,3^. Among of the heterogeneous catalysts studied, zeolites especially ZSM-5 or modified ZSM-5 are the promising catalysts as they can be tuned to exhibit higher activity than the traditional alumina-based catalysts^4–9^: the reaction temperature is lower and a low concentration of ethanol aqueous solution can be used. The formation of ethene is the first step in ethanol dehydration; subsequent polymerization, cracking and aromatization by the secondary reaction of ethene leads to the formation of longer-chain hydrocarbons^10^, very similar to the formation of hydrocarbons in the conversion of methanol over zeolites^11,12^. The detailed knowledge of the reaction mechanism of ethene formation in ethanol dehydration is important not only for understanding ethanol dehydration as a model reaction of alcohol conversion but also for the development of improved catalysts for light olefins production.

The formation of ethene in the initial dehydration process has been experimentally^13–15^ and theoretically^16–19^ investigated. A parallel-consecutive route was proposed^3^: dehydration of ethanol to ethene and diethyl ether in parallel and a consecutive decomposition of diethyl ether to ethene. In the unimolecular dehydration of ethanol to ethene, it was proposed that the acidic proton transfers from the zeolite to the OH group on ethanol, which subsequently releases water to form an intermediate such as carbocation or an alkoxide bound to zeolite. Solid-state NMR and infrared spectroscopic studies^16,20,21^ confirmed the formation of surface-bound ethoxy species as a stable intermediate in ethanol dehydration of on H-ZSM-5 and HY zeolites. Ethene and hydrocarbons are formed by the subsequent decomposition of the surface ethoxy species.

Diethyl ether (DEE) is concurrently produced with ethene, and the kinetic and spectroscopic studies revealed that intermolecular dehydration of ethanol leads to DEE via dimeric ethanol species or reaction of an ethoxy group with undissociated ethanol on zeolites^13,22,23^. The following cracking of DEE produces ethene, which occurs preferably at lower reaction temperatures as compared to the unimolecular dehydration of ethanol to ethene^14,24^. Note that the ethoxy species was generally proposed to be the direct precursor to ethene. However, the generation of such species is still not well understood. Thus, although different reaction schemes have been proposed, the detailed mechanism of the initial step of ethanol dehydration remains elusive, particularly for the favorable production of ethene from DEE at lower temperature.

Here we report the observation and identification of reaction intermediates in ethanol dehydration to ethene over zeolite H-ZSM-5. The ex situ and in situ solid-state NMR spectroscopy allows for exploration of the dehydration of ethanol at the initial stage. A triethyloxonium ion (TEO) is observed as a potential active intermediate on the working catalyst. We provide evidence for the involvement of TEO in the formation of surface ethoxy species and then ethene on the zeolite. The elementary reactions leading to the formation of ethene are identified by combining experiments and theoretical calculations, which suggests that a TEO-mediated reaction route is operative for the dehydration of ethanol to ethene.

## Results

### Observation of TEO

H-ZSM-5 (Si/Al = 11.5, Zeolyst) was used for the dehydration of ethanol. The XRD and ^27^Al and ^29^Si solid-state NMR spectra show structure information of the H-ZSM-5 catalyst (Supplementary Fig. 1 and 2). The acidic property was examined by FT-IR and NH_3_-TPD (Supplementary Fig. 3 and 4). The reactions were conducted in a pulse-quench reactor^25^, in which ethanol was pulse-injected at temperatures ranging from 160 to 350 °C and allowed to react for 4 s under continuous He carrier gas flow before the reaction was thermally quenched by pulsing liquid nitrogen onto the catalyst bed. The reaction effluent was analyzed by GC (Supplementary Fig. 5). Ethene is the only product at 160–200 °C, indicating that the dehydration of ethanol to ethene is favorable at lower temperature. With increasing reaction temperature, the secondary reaction of ethene leads to the formation of C_3_^+^ products including olefins and aromatics.

**Fig. 1:**
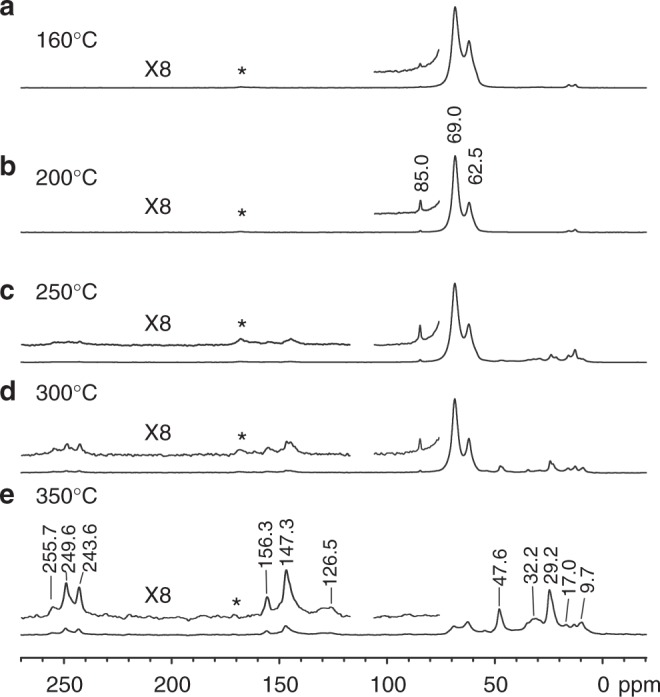
^13^C NMR analysis of the formed surface species on zeolite. ^13^C CP/MAS NMR spectra of trapped species on H-ZSM-5 obtained from reaction of CH_3_^13^CH_2_OH for 4 s at different temperatureof 160 °C–350 °C (**a**-**e**). Asterisks denote spinning sidebands

Figure 1 shows the ex situ ^13^C CP/MAS NMR spectra of trapped products from the dehydration of ^13^C-1-enriched ethanol (CH_3_^13^CH_2_OH, 99% ^13^C) on H-ZSM-5 at 160–350 °C for 4 s. Ethanol and DEE are observed as reflected by the strong signals at 62.5 and 69.0 ppm respectively, due to the ^13^C-enriched methylene carbons; the methyl carbons produce the weak signals at 12–17 ppm. The adsorbed species formed at 200 °C were further analyzed by two-dimensional (2D) ^1^H–^13^C HETCOR NMR experiment (Supplementary Fig. 6). A weak signal at 72.8 ppm that was seriously overlapped by the ethanol signal (69.0 ppm) in the 1D ^13^C NMR spectra was clearly discerned in the 2D spectrum, which can be ascribed to the methylene carbon of ethoxy group. The formation of the alkoxy species was well established in methanol and ethanol dehydration on acidic zeolites. Hunger et al.^20^ reported the surface ethoxy species from ethanol dehydration on HY zeolite, which has a chemical shift of 72.6 ppm.

Interestingly, a well-resolved signal appears at 85.0 ppm (Fig. 1) and grows up at intermediate temperature. The oligomeric alkoxy species formed by the oligomerization of ethene on acidic zeolites were previously proposed to produce broad signals at 78.0 to 90.0 ppm in the literatures^20,26^. Since there is no any C_3+_ species formed at this reaction condition, the oligomerization of ethene did not likely occur in our case. Furthermore, no methylene signals at 20.0 to 40.0 ppm can be observed that are supposed to be oligomerized compounds^27^. Therefore, the 85.0 ppm signal should not be ascribed to the oligomeric alkoxy species. Instead, we proposed on the basis of its chemical shift the formation of alkyl-substituted oxonium ions. Munson and Haw reported the formation of trimethyloxonium ion (TMO) with a characteristic ^13^C NMR signal at 80.0 ppm by the reaction of dimethyl ether on H-ZSM-5^28^, and they also confirmed that TMO was not an intermediate in the methanol to hydrocarbons conversion. Since DEE is readily formed and in equilibrium with ethanol (Fig. 1b), we assume that a triethyloxoium ion (TEO) may be formed.

**Fig. 2:**
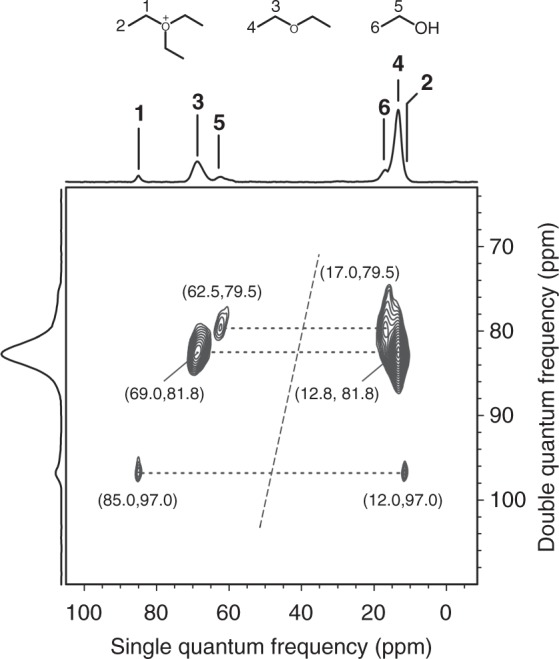
Identification of triethyloxoium ion by correlation NMR spectroscopy. 2D ^13^C–^13^C INADEQUATE MAS NMR spectrum of trapped species obtained from the reaction of ^13^CH_3_^13^CH_2_OH on H-ZSM-5 at 220 °C for 4 s

To gain insights into the structure of TEO, a ^13^C–^13^C *J*-based refocused INADEQUATE (Incredible Natural Abundance Double Quantum Transfer Experiment)^29^ MAS NMR experiment was performed, which provides an unambiguous identification of bond connectivity of carbon species. The INADEQUATE spectrum (Fig. 2) of H-ZSM-5 reacted with ^13^CH_3_^13^CH_2_OH at 200 °C for 4 s exhibits a clear correlation peak pair between the methylene carbon at 85.0 ppm and the methyl carbon at 12.0 ppm, confirming the formation of TEO that is composed of ethyl groups. Two other correlations are identified, showing the connectivity of the methylene carbons of DEE (69.0 ppm) and ethanol (62.5 ppm) to the corresponding methyl carbons at 12.8 and 17.0 ppm, respectively. The structure of TEO was also theoretically optimized in the H-ZSM-5 channel by periodic density functional theory (DFT) calculations (Supplementary Fig. 7). The ^13^C chemical shift of the methylene carbon of TEO is predicted to be 84.6 ppm, in good agreement with the experimental value. Therefore, our NMR experiments and theoretical calculations provide solid evidence for TEO formation in the ethanol dehydration process.

For the adsorbed species formed on H-ZSM-5 after reaction at 200 °C for 4 s, the amount of TEO (85.0 ppm), ethoxy (72.8 ppm), DEE (69.0 ppm) and ethanol (62.5 ) was determined by ^13^C NMR experiment to be 0.34, 0.46, 28.76 and 6.99 mmol/g, respectively. TEO and the ethoxy species are solely formed and located on Brønsted acid sites for charge compensation, while DEE and ethanol are trapped over zeolite via either chemical or physical adsorption. Considering the total Brønsted acid sites (about 1.03 mmol/g) on the catalyst, the amount (less than 0.23 mmol/g) of DEE adsorbed on Brønsted acid sites should be lower than that (0.34 mmol/g) of TEO at this reaction condition. At temperature above 300 °C (Fig. 2d, e), the 85.0 ppm signal is almost unobservable, which can be ascribed to the decomposition of TEO. In the meantime, the secondary reaction of ethene produces the dominating C_3+_ hydrocarbons, reflected by the strong high-field signals below 30.0 ppm. The signals at 147.3–156.3, 243.6–255.7 and 47.6 ppm are characteristics of the cyclic cations such as dimethylcyclopentenyl and ethylcyclopentenyl ions^30–32^, which are usually involved in the formation of aromatics. This implies that the aromatics-based cycle prevails in a similar route to the conversion of methanol to hydrocarbons^33,34^.

### Reactivity and intermediate role of TEO

**Fig. 3:**
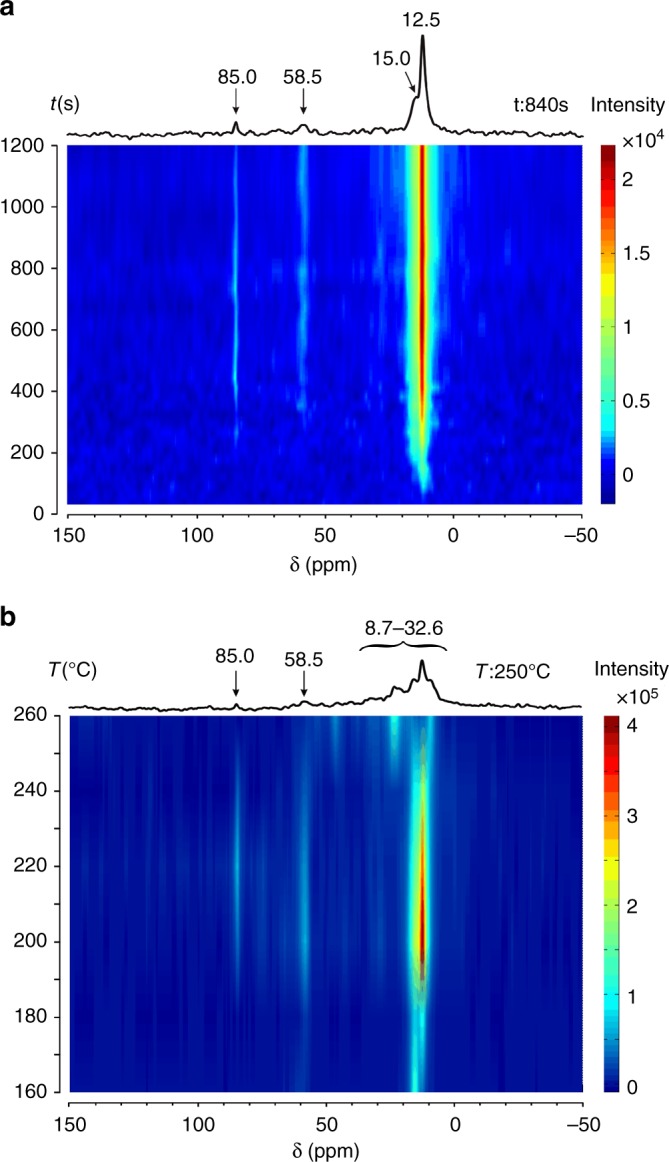
Real-time NMR monitoring of ethanol dehydration on zeolite. In situ ^13^C MAS NMR spectra of ^13^CH_3_^13^CH_2_OH conversion on H-ZSM-5 with time on stream (**a**) and at elevating temperatures (**b**). Spectra (**a**) were recorded at every 30 s from 0 to 1200 s at 220 °C and 30 scans were accumulated for each time slice. Spectra (**b**) were recorded at every 10 °C from 160–260 °C and 900 scans were accumulated for each temperature slice. The 1D spectra on the top are recorded at 840 s (**a**) and 250 °C (**b**), respectively

In order to explore the reactivity of TEO, continuous-flow ^13^CH_3_^13^CH_2_OH conversion over H-ZSM-5 was investigated by in situ ^13^C NMR spectroscopy (see methods section for details of the in situ NMR experiments). Figure 3 shows the real-time monitoring of formed surface species on H-ZSM-5 in the ethanol dehydration process. At a constant reaction temperature of 220 °C (Fig. 3a), the appearance of 85.0 ppm signal after 200 s points to the formation of TEO. The broad signal centered at 58.5 ppm should come from the adsorbed ethanol, whose methyl group produces a high-field strong signal at 15.0 ppm. Note that the methylene group of DEE (69.0 ppm) is invisible in the in situ experiment. This is probably due to the strong adsorption of DEE on zeolite, leading to large anisotropic interactions (chemical shifts and nuclear dipole-dipole couplings) which cannot be efficiently removed by the low magic spinning speed (2 kHz). However, the formation of DEE is evidenced by the 12.5 ppm signal due to its methyl group featured by high mobility. TEO is observable with increasing the reaction time up to 1200 s (20 min), indicative of its stability at the moderate reaction temperature. The GC analysis of the effluent products obtained from the reaction with time-on-stream shows the formation of ethene and a small amount of higher hydrocarbons (Supplementary Fig. 8). Figure 3b shows the in situ ^13^C NMR spectra obtained at elevating temperatures. The TEO is observable over a wide temperature range. The formation of hydrocarbons prevails at higher temperatures as reflected by a set of high-filed signals (8.7–32.6 ppm) while the TEO is significantly reduced due to its decomposition, consistent with the ex situ NMR result (Fig. 1). In comparison, the structurally similar species TMO formed on H-ZSM-5 was readily decomposed before the onset of hydrocarbon synthesis^35,36^.

**Fig. 4:**
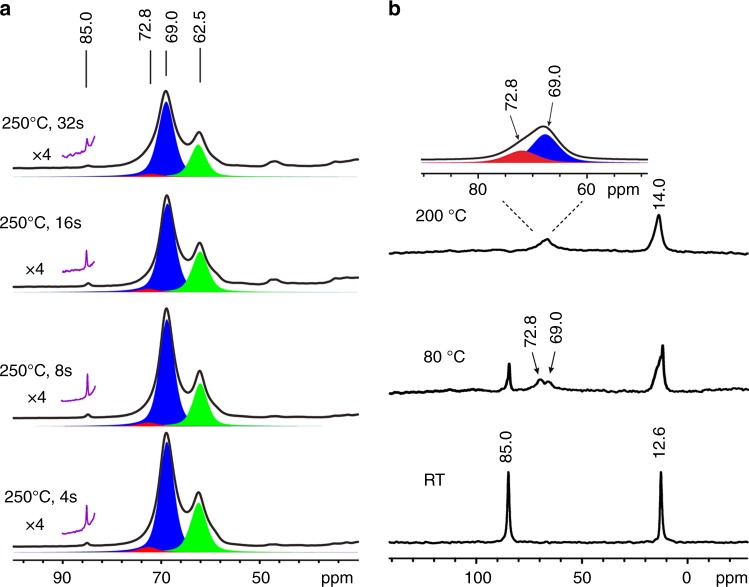
Evolution of TEO in the formation of ethene. ^13^C CP MAS NMR spectra of trapped species on H-ZSM-5 obtained from pulse-quench reactions of CH_3_^13^CH_2_OH at 250 ^o^C for different time (**a**) and ^13^C MAS NMR spectra of TEO-ZSM-5 at room temperature (RT), heated at 80 °C for 5 min, and at 200 °C for 5 min (**b**). The deconvoluted components are indicated in color

To confirm the intermediate role of TEO in the formation of ethene, the ^13^C NMR spectra of trapped species obtained from the pulse-quench reactions of CH_3_^13^CH_2_OH were recorded during the reaction course of 4~64 s at 250 °C. Figure 4a shows the expanded spectra and their deconvolutions (see Supplementary Fig. 9 for the whole spectra), in which the formation and evolution of TEO (85.0 ppm) is evident. We know from above experiments that the ethoxy species (72.8 ppm) is simultaneously produced, which is confirmed by 2D ^1^H–^13^C HETCOR MAS NMR (Supplementary Fig. 6). Comparison of the integrated ^13^C signal intensities shows that the ethoxy species keeps a similar evolution trend with TEO (Supplementary Table 1). Thus, the formation of surface ethoxy species is most likely related to TEO. Furthermore, TEO was deliberately prepared by an ion exchange of triethyloxonium tetrafluoroborate with H-ZSM-5. The formation of surface TEO on H-ZSM-5 is confirmed by its characteristic signal at 85.0 ppm in the ^13^C NMR spectra (Fig. 4b). The concentration of TEO determined by ^13^C MAS NMR is about 1 mmol/g, comparable to the amount (1.03 mmol/g) of Brønsted acid site. Importantly, the isolation of stable TEO at room temperature allows us to unambiguously trace its reactivity and transformation on the catalyst. After heating, TEO was partially converted into ethoxy species (at 72.8 ppm) and DEE (at 69.0 ppm) at lower temperature of 80 °C, while higher temperature (200 °C) led to its complete conversion. We also obtained the activation energy for the formation of ethoxy species from TEO by measuring the rate constants at different temperatures using ^13^C MAS NMR spectroscopy (Supplementary Fig. 10). The experimentally determined activation energy is ca.77.3 kJ/mol. It is worth noting that a large quantity of ethene was observed in the effluent product upon the consumption of TEO by GC (Supplementary Fig. 11). These results demonstrate a direct correlation of TEO to ethoxy species and ethene product.

### Mechanism of ethene formation via TEO

**Fig. 5:**
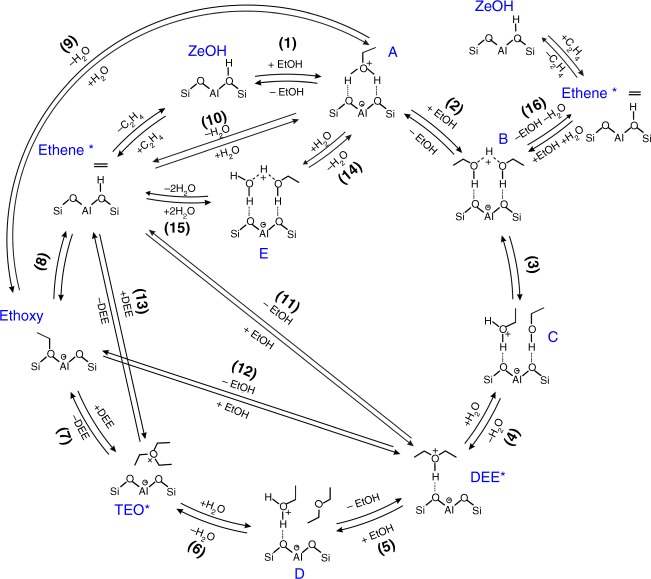
Proposed catalytic cycle for ethanol dehydration to ethene. The structures of transition states (TS) involved in these reaction steps are depicted in Supplementary Fig. 13. * indicates the adsorbed state

Our experimental observations indicate a strong potential of TEO serving as an active intermediate in the formation of ethene. A whole reaction network for ethanol dehydration to ethene was proposed (Fig. 5), which includes the previously theoretically predicted unimolecular and bimolecular processes^17,19^ and our proposed TEO-mediated routes. It consists of three types of mechanisms: direct ethanol dehydration to ethene (route 5, 6, 7 and 8), diethyl ether decomposition to ethene (route 3 and 4) and TEO-mediated ethene formation (route 1 and 2). DFT calculations were performed to explore the most favorable ethene formation route and the optimized transition state structures on the zeolite cluster model (Supplementary Fig. 12) are displayed in Supplementary Fig. 13. Our calculations show that all the kinetic steps of ethanol dehydration are endothermic processes (Supplementary Table 2). The calculated Gibbs free energy barrier (Supplementary Fig. 14–18 for the energy diagrams), pre-exponential factor, and rate coefficient at 473 K of the forward and reverse reactions are tabulated in Table 1. The comparison of energy barriers and rate coefficients indicates that the direct dehydration processes (steps 9, 10, 15 and 16) are energetically less favorable for ethene formation, in agreement with the previous work^17^.

**Table 1:** Reaction Gibbs free energy barrier (∆*G*_*a*ct_, kJ mol^−1^), pre-exponential factor(*A*, s^−1^), and rate coefficient (*k*, s^−1^) at 473 K of the forward (*f*) and reverse reactions (*r*) for the elementary step associated with the eight ethene production routes

	Elementary step	∆*G*_act(*f*)_	∆*G*_act(*r*)_	*A*_*f*_	*k*_*f*_	*A*_*r*_	*k*_*r*_
Route 1	${\rm{C}}\mathop { \leftrightarrow }\limits^{{\rm{TS4}}} {\rm{DEE}}{\rm{.H}}_2{\rm{O}} \ast$ (4)	129.0	79.4	6.43 × 10^11^	5.54 × 10^−2^	4.20 × 10^12^	1.70 × 10^4^
	${\rm{D}}\mathop { \leftrightarrow }\limits^{{\rm{TS6}}} {\rm{TEO}}{\rm{.H}}_2{\rm{O}} \ast$ (6)	94.1	65.0	1.19 × 10^13^	3.95 × 10^2^	8.68 × 10^11^	6.45 × 10^5^
	${\rm{TEO}} \ast \mathop { \leftrightarrow }\limits^{{\rm{TS7}}} {\rm{Ethoxy}}{\rm{.DEE}} \ast {\rm{(7)}}$	81.7	66.6	1.29 × 10^12^	9.61 × 10^3^	9.33 × 10^11^	4.38 × 10^5^
	${\rm{Ethoxy}}\mathop { \leftrightarrow }\limits^{{\rm{TS8}}} {\rm{Ethene}} \ast$ (8)	107.6	75.3	1.32 × 10^13^	1.30 × 10^1^	1.91 × 10^11^	4.74 × 10^4^
Route 2	${\rm{C}}\mathop { \leftrightarrow }\limits^{{\rm{TS4}}} {\rm{DEE}}{\rm{.H}}_2{\rm{O}} \ast$ (4)	129.0	79.4	6.43 × 10^11^	5.54 × 10^−2^	4.20 × 10^12^	1.70 × 10^4^
	${\rm{D}}\mathop { \leftrightarrow }\limits^{{\rm{TS6}}} {\rm{TEO}}{\rm{.H}}_2{\rm{O}} \ast$ (6)	94.1	65.0	1.19 × 10^13^	3.95 × 10^2^	8.68 × 10^11^	6.45 × 10^5^
	${\rm{TEO}} \ast \mathop { \leftrightarrow }\limits^{{\rm{TS13}}} {\rm{Ethene}}{\rm{.DEE}} \ast {\rm{(13)}}$	127.7	78.4	2.23 × 10^13^	7.81 × 10^−2^	9.32 × 10^9^	2.17 × 10^4^
Route 3	${\rm{C}}\mathop { \leftrightarrow }\limits^{{\rm{TS4}}} {\rm{DEE}}{\rm{.H}}_2{\rm{O}} \ast$ (4)	129.0	79.4	6.43 × 10^11^	5.54 × 10^−2^	4.20 × 10^12^	1.70 × 10^4^
	${\rm{DEE}} \ast \mathop { \leftrightarrow }\limits^{{\rm{TS12}}} {\rm{Ethoxy}}{\rm{.EtOH}} \ast {\rm{(12)}}$	155.9	78.2	7.12 × 10^12^	6.01 × 10^−5^	5.11 × 10^11^	2.26 × 10^4^
	${\rm{Ethoxy}}\mathop { \leftrightarrow }\limits^{{\rm{TS8}}} {\rm{Ethene}} \ast$ (8)	107.6	75.3	1.32 × 10^13^	1.30 × 10^1^	1.91 × 10^11^	4.74 × 10^4^
Route 4	${\rm{C}}\mathop { \leftrightarrow }\limits^{{\rm{TS4}}} {\rm{DEE}}{\rm{.H}}_2{\rm{O}} \ast$ (4)	129.0	79.4	6.43 × 10^11^	5.54 × 10^−2^	4.20 × 10^12^	1.70 × 10^4^
	${\rm{DEE}} \ast \mathop { \leftrightarrow }\limits^{{\rm{TS11}}} {\rm{Ethene}}{\rm{.EtOH}} \ast {\rm{(11)}}$	136.1	58.9	1.03 × 10^14^	9.24 × 10^−3^	5.11 × 10^11^	3.06 × 10^6^
Route 5	${\rm{A}}\mathop { \leftrightarrow }\limits^{{\rm{TS9}}} {\rm{Ethoxy}}{\rm{.H}}_2{\rm{O}} \ast {\rm{(9)}}$	146.3	69.8	6.75 × 10^13^	6.81 × 10^−4^	4.79 × 10^12^	1.91 × 10^5^
	${\rm{Ethoxy}}\mathop { \leftrightarrow }\limits^{{\rm{TS8}}} {\rm{Ethene}} \ast$ (8)	107.6	75.3	1.32 × 10^13^	1.30 × 10^1^	1.91 × 10^11^	4.74 × 10^4^
Route 6	${\rm{A}}\mathop { \leftrightarrow }\limits^{{\rm{TS10}}} {\rm{Ethene}}{\rm{.H}}_2{\rm{O}} \ast {\rm{(10)}}$	171.8	103.3	4.28 × 10^13^	1.04 × 10^−6^	2.96 × 10^10^	3.81 × 10^1^
Route 7	${\rm{E}}\mathop { \leftrightarrow }\limits^{{\rm{TS15}}} {\rm{Ethene}}{\rm{.2H}}_2{\rm{O}} \ast$ (15)	184.8	64.7	1.15 × 10^14^	3.89 × 10^−8^	1.71 × 10^11^	6.98 × 10^5^
Route 8	${\rm{B}}\mathop { \leftrightarrow }\limits^{{\rm{TS16}}} {\rm{Ethene}}{\rm{.H}}_2{\rm{O}}{\rm{.EtOH}} \ast {\rm{(16)}}$	145.2	119.2	5.37 × 10^13^	9.01 × 10^−4^	1.89 × 10^10^	6.76 × 10^−1^

Compared with the direct ethanol dehydration routes (route 5, 6, 7 and 8), the two molecules mediated route 4 including dimer-mediated etherification (∆*G*_act(*f*)_ = 129.0 kJ/mol, *k*_*f*_ = 5.54 × 10^−2^ s^−1^, step 4) and the following DEE decomposition to ethene (∆*G*_act(*f*)_ = 136.1 kJ mol^−1^, *k*_*f*_ = 9.24 × 10^−3^ s^−1^, step 11) is favorable for ethanol dehydration to ethene, in agreement with the previous report that the reaction path via DEE is preferentially involved in ethene formation at temperatures lower than 500 *K*^17^. It is reasonable to assume that TEO would be readily formed due to the facile formation of DEE. This is confirmed by a free energy barrier of ca. 94.1 kJ mol^−1^ for the formation of TEO from DEE (step 6), which is much lower than that of DEE decomposition routes (∆*G*_act(*f*)_ = 136.1 kJ/mol for step 11 in route 4 and ∆*G*_act(*f*)_ = 155.9 kJ mol^−1^ for step 12 in route 3). We have experimentally observed that the ethoxy species and DEE can be produced from TEO on H-ZSM-5 (Fig. 4). This can be understood by the fact that TEO ethylates the conjugate base site of zeolite to form a framework-bound ethoxy species with the release of a DEE, which shows the characteristic property of trialkyloxonium ions acting as an powerful alkylating agent^37^. This process is calculated to have an activation energy of 73.6 kJ mol^−1^ (step 7, Supplementary Table 2), in good agreement with our experimental value (77.3 kJ mol^−1^), strongly supporting the formation of ethoxy via TEO intermediate. The low free energy barrier of step 8 (107.6 kJ mol^−1^) suggests the transformation of ethoxy species to ethene can readily occur. Moreover, the calculated electronic energy barrier for the conversion of ethoxy species to ethene (127.6 kJ mol^−1^, Supplementary Table 2) is similar to the experimentally estimated value (ca. 122 kJ mol^−1^) for the decomposition of ethoxy species to ethene on H-ZSM-5^38^. The direct decomposition of TEO into ethene (step 13 in route 2) is also considered, which is however characterized by a higher free energy barrier (127.7 kJ mol^−1^). A full comparison of free energy barriers and rate coefficients of the rate-determination steps in the proposed reaction network indicates that route 1 (steps 4, 6, 7, 8) and route 2 (steps 4, 6, 13) involving TEO intermediate are the most favorable routes. Thus, concerning the overall kinetics during the ethanol dehydration to ethene, the presence of TEO provides an energetically preferable process at lower reaction temperature.

## Discussion

The dehydration of ethanol to ethene over H-ZSM-5 has been investigated by combined experiments and DFT calculations. Our ex situ and in situ solid-state NMR data provide the evidence for the formation of TEO in the dehydration process. Generally, onium ions that are often isolable as salts are well recognized as reaction intermediates^37^. It was reported that trimethyloxonium can be generated on H-ZSM-5 zeolite. However, mechanistic significance of trimethyloxonium is uncertain since its intermediate role for the initial C–C bond formation in methanol conversion was excluded based on experimental observations^27,28^. Herein, TEO is formed as a stable surface species on the working catalyst in both continuous-flow and pulse-quench reactions. We calculated the host-guest interaction free energy of TEO confined in the H-ZSM-5 channel, which is as low as −360.2 kJ mol^−1^ at 473 K, indicating that the zeolite framework can stabilize TEO intermediate effectively.

The implications of the observation of TEO in ethanol dehydration to ethene in present work are manifold. The ethoxy species is often experimentally observed during ethanol dehydration on zeolite, which is generally considered as the precursor to ethene in both processes of unimolecular dehydration of ethanol and bimolecular dehydration of ethanol followed by DEE decomposition. DEE is largely formed in the initial stage of dehydration of ethanol and its coverage on acid sites is estimated to be comparable to TEO at lower temperature (e.g., 473 K). The presence of TEO provides an energetically favorable mechanism to link DEE and ethoxy species, which is viable for the formation of ethene at low temperature. In the onium-ylide mechanism proposed for the C–C bond formation from C1 compound such as methanol and DME on zeolite, Olah et al. predicated the intermediate formation of ethyldimethyloxonium, which could undergo β-elimination to yield ethene and DME^39^. Analogously, the direct deprotonation and decomposition of TEO could lead to ethene and DEE on zeolites (route 2), which, however, occurs with a relatively higher energy barrier. We have experimentally shown that TEO tends to alkylate basic site (Si–O^−^–Al) of zeolite to form surface ethoxy species, which in nature resembles the alkyloxonium salt featured by strong alkylating ability in organic synthesis. This result indicates the reactivity of alkyloxonium ion could be altered by the zeolite catalyst, which leads to different reaction routes. Although the alkyloxonium ion (i.e., a protonated alcohol) has long been proposed as a key intermediate in the dehydration of alcohol to produce alkenes and ethers particularly in strong liquid acid systems, the detailed characterizations of TEO in the ethanol dehydration process in this work provide an example of uncovering the important role of this kind of intermediate in the formation of ethene on zeolite catalysts, which may open avenues for further experimental and theoretical exploration of the oxonium ions chemistry in alcohol conversion.

## Methods

### X-ray power diffraction (XRD)

The ammonium form ZSM-5 (Si/Al = 11.5, obtaind from Zeolyst) was calcined in air at 823 K for 5 h to obtain the proton form H-ZSM-5. The structure and crystalline nature of H-ZSM-5 zeolites were examined by X-ray diffractometer (X’Pert^3^ Powder) using a CuKα radiation with a step of 0.02° at a respective voltage of 40 kV and a current of 40 mA.

### FT-IR of pyridine adsorption

The FT-IR of pyridine adsorption measurements were performed on a Bruker Tensor 27 spectrometer. The catalysts were first activated at 773 K under high vacuum (<10^−5^ Pa) for 12 h. After cooling down to 313 K, a background spectrum was collected. Excessive amount of pyridine was then introduced to the infrared cell and held for 2 h to allow equilibrium. The residual pyridine was removed by vaccum. The FT-IR spectra of pyridine-adsorbed samples were measured at 313 K after evacuation at 373, 473, 573, 673 and 773 K, respectively.

### Temperature-programmed desorption of ammonia (NH_3_-TPD)

The NH_3_-TPD measurement was performed using a FINESORB-3010 chemisorption instrument. Typically, 100 mg of sample was pre-treated at 423 K for 1 h under 30 sccm of helium gas. After the sample cooled down to 323 K, NH_3_ was introduced and held for 1 h. The temperature was then elevated from 323 to 1023 K at a ramping rate of 10 K/min and the desorbed NH_3_ was detected by a thermal conductivity detector (TCD).

### Catalytic testing

The H-ZSM-5 powder was pressed into pellets between 60–80 mesh. The pellets (0.2 g) were activated at 400 °C in flowing helium for 1 h prior to the ethanol dehydration reactions. A pulse-quench reactor was used to quench the reaction by reducing the reaction temperature with liquid nitrogen within a very short period (<1 s)^25^. Typically, when the reaction proceeded in a pulse-quench reactor for a pre-set period, the reaction was thermally quenched by pulsing liquid nitrogen onto the catalyst bed, which was achieved by using high-speed valves controlled by GC computer. In each case, 10 μl of reactant was pulsed into the reactor (heated at 160–350 °C) containing 0.2 g H-ZSM-5 and allowed to react for different time, before quenching by liquid nitrogen. The trapped surface species were analyzed by ^13^C solid-state NMR spectroscopy and the effluent products were on line determined by GC-MS analysis. For the continuous flow reaction, ethanol with a weight hourly space velocity (WHSV) of 2 h^−1^ was reacted over the H-ZSM-5 (0.2 g) pellets in a fixed bed reactor at the temperature range of 140–260 °C.

### Gas chromatography

The effluent products drawn from the flowing gas were analyzed quantitatively by online GC-FID chromatograph (Shimadzu GC-2010 plus) which equipped with a flame-ionization detector and a Supelco Supel-Q^TM^ PLOT capillary column (30 m × 0.32 mm × 15 μm). The initial temperature programming started at 40 °C (maintained for 2 min), followed by a rate of 5 °C min^−1^ to 132.5 °C and a rate of 10 °C min^−1^ to the final temperature of 220 °C. The retained products in the catalyst was directly analyzed by solid-state NMR spectroscopy (see the following).

### Preparation of triethyloxoium ion (TEO) on H-ZSM-5

A concentration of 0.2 g of H-ZSM-5 zeolite (Si/Al = 11.5) was dehydrated on a vacuum line (<10^−3^ Pa) at 400 °C for 12 h. After dehydration, the sample was allowed to cool down to room temperature for subsequent use. A concentration of 0.3 g triethyloxonium tetrafluoroborate (Aldrich) was added into 3 g dry dichloromethane solvent, and then the dehydrated zeolite sample was added into the solution. All the operations were carried out in a glovebox filled with pure N_2_. After ultrasonic treatment of the mixture in ice water bath at 0 °C for 20 min, the mixtrue was filtrated and evacuated completely and the sample was dried at room tempearture for 5 h by vacuum. The obtianed triethyloxoium ion (TEO) exchanged ZSM-5 zeolite was denoted as TEO-ZSM-5.

### Solid-State NMR experiments

After the reaction was quenched, the pulse-quench reactor containing the catalyst was sealed. The sealed reactor was then transferred to a glove box filled with pure N_2_ and the catalyst was packed into to an NMR rotor for ex situ NMR measurements. To enhance the detection sensitivity, ^13^C labeled ethanol was used for ^13^C NMR experiments in both the pulse-quench and the in situ reactions.

All the ex-situ ^13^C solid-state NMR spectroscopy experiments were carried out at 9.4 T on a Bruker Avance III-400 spectrometer, equipped with a 4 mm probe, with resonance frequencies of 399.33 and 100.42 MHz for ^1^H and ^13^C, respectively. The magic angle spinning rate was set to 10 kHz. For the ^1^H → ^13^C CP/MAS NMR experiments, the Hartmann-Hahn condition was achieved using hexamethylbenzene (HMB), with a contact time of 5 ms and a repetition time of 2 s. The hpdec ^13^C MAS NMR experiments were performed using a ^13^C 90-degree pulse length of 5 μs and a recycle delay of 5 s. The ^13^C chemical shifts were referenced to HMB (a second reference to TMS).

The ^27^Al MAS NMR spectra were also acquired on the same 4 mm probe by small-flip angle technique with a pulse length of 0.3 μs (<π/12) and a recycle delay of 1 s. The magic angle spinning rate was set to 10 kHz. The ^27^Al chemical shifts were referenced to 1 M Al(NO_3_)_3_ aqueous solution (0 ppm). ^29^Si MAS NMR experiments were carried out at 7.1T on a Varian Infinity plus-300 spectrometer, with resonance frequencies of 299.78 and 70.11 MHz for ^1^H and ^29^Si, respectively. Single-pulse ^29^Si MAS NMR spectra with high power proton decoupling were recorded on a 7.5 mm probe, using a π/2 pulse of 5 μs, a recycle delay of 80 s and a spinning rate of 4 kHz. The ^29^Si chemical shifts were referenced to kaolinite (−91.5 ppm).

2D ^1^H –^13^C CP HETCOR experiment was performed using a Avance III 800 spectrometer operating at a ^1^H Larmor frequency of 800.36 MHz and a 4 mm HCN E-free probe at a spinning frequency of 12 kHz. The ^1^H *π*/2-pulse length was 4.85 μs. ^13^C magnetization was created using a cross-polarization (CP) ramp of magnitude 80 to 100% with a contacted time of 5 ms. 256 transients were co-added using a recycle delay of 2 s. A total of 256 *t*_*1*_ FIDs were recorded at increments of 3.19 ms using the States-TPPI method to achieve sign discrimination in F1. ^1^H SPINAL-64^40^ decoupling was applied during the *t*_*2*_ acquision with a RF-field amplitude of 51.5 kHz.

2D ^13^C-^13^C CP *J*-refocused INADEQUATE^29^ MAS NMR experiment was carried out using a Avance III 800 spectrometer at a MAS speed of 8 kHz. The ^1^H π/2-pulse length was 2.75 us. ^13^C magnetization was created using a cross-polarization (CP) ramp of magnitude 80 to 100% with a contacted time of 7 ms. ^13^C π/2 and π pulses were 4.8 us and 9.6 µs, respectively. The *J*-evolution τ periods were rotor synchronized and set to 3.24 ms. 64 transients were co-added using a recycle delay of 3 s. A total of 160 *t*_*1*_ FIDs were recorded at increments of 3.97 ms using the States-TPPI method to achieve sign discrimination in F1. ^1^H SPINAL-64 decoupling with a RF-field amplitude of 90.91 kHz was employed after CP covering all *t*_*1*_, *J*-evolution and *t*_*2*_ periods. The two frequency axes in the 2D spectra are used to assign the ^13^C resonances corresponding to through-bond ^13^C-^13^C connectivities, which allows for unambiguous assignment and structure determination of organic compounds.

All the in situ ^13^C solid-state NMR experiments were performed at 11.7 T on a Bruker Avance III 500 spectrometer, equipped with a 7 mm H/X MASCAT probe (Fig. S19), with resonance frequencies of 500.58 and 125.87 MHz for ^1^H and ^13^C, respectively. The magic angle spinning rate was set to 2 kHz. Prior to the in situ NMR experiments, 0.2 g of pre-dehydrated H-ZSM-5 was pressed into a 7 mm NMR rotor. An axial hole of 2.5 mm in diameter was made into the sample with a special tool, by which an uniform annular catalyst bed was formed in the rotor. After inserting the injection tube into the MAS rotor, the rotor was heated at 280 °C for 1 h via the bearing gas, keeping helium (200 ml min^−1^) injected into the rotor, then cooled down to the reaction temperature. ^13^C labelled ethanol (^13^CH_3_^13^CH_2_OH, 98% ^13^C, Sigma-Aldrich) was diluted to 50% (v/v) with ethanol in natural aboundance. The diluted ^13^C labelled ethanol was fed into the NMR rotor by the helium carrier gas (weight hourly space velocity (WHSV) = 2.2 h^−1^) through a saturator. The reactants flow inside the rotor via the injection tube and pass through the catalyst from the bottom to the top and leave the rotor via an exhaust tube in the rotor cap. The ^13^C CP/MAS NMR spectra were recorded with a contant time of 5 ms and a recycle delay of 1 s. 30 and 900 scans were accumulated for each spectrum at different time and temperature respectively. The ^13^C chemical shifts were referenced to HMB (a second reference to TMS). The in situ ^13^C MAS NMR spectra in Fig. 3 were generated by the overlay of 1D NMR spectra as a function of reaction time or temperature. The correlation between ^13^C resonances and the reaction parameters (time and temperature) was followed in the 2D maps, which facilitates the monitoring of the fate of the organic species during the time or temperature evolutions.

The activation energy of the formation of ethoxy species from TEO on H-ZSM-5 was measured as follows. The sealed NMR rotor containing the TEO-ZSM-5 sample was heated at a specific temperature (from 328 to 348 K) for a period of time, and then the reaction was quenched by liquid N_2_. ^13^C MAS NMR measurement was performed at room temperature. At such low reaction temperature, only ethoxy species and diethyl ether were produced on the TEO-ZSM-5. The concentration of TEO during the reaction was measured by normalized integrated NMR signal at 85 ppm. Finally, the temperature dependent rate constant was obtained and the activation energy was derived from the Arrhenius equation.

### Theoretical calculations

The host-guest interactions between the cations and zeolite framework would result in the ^13^C chemical shift moving due to the re-distributions of electronic environment invoked by the H-ZSM-5 confined pore. Thus, to unambiguously assign the experimental NMR results, the ^13^C NMR chemical shifts of the cations are further calculated using the periodic boundary condition.

The geometry optimizations were performed by using the CASTEP program^41^ with the generalized gradient approximation (GGA) proposed by Perdew–Burke–Ernzerhof (PBE) functional. And the ultrasoft pseudo potential, fine plane wave cut-off energy (340 eV) and a default fine level Monkhorst-Pack K-point (1 × 1 × 1) were adopted to sample the Brillouin zone. DFT-D method^42^ was used in the structure optimization and the NMR calculation to accurately describe the weak interaction in H-ZSM-5. During optimization, the 22T active site atoms and the adsorbed cation were relaxing to their equilibrium positions. All of the NMR shielding calculations were performed by the GIPAW method in the MS CASTEP-NMR code at the GGA/PBE level based on the optimized zeolite structures. All of the ^13^C chemical shifts (δ ^13^C) cal were derived using the CASTEP-NMR module available in the Materials Studio package based on the optimized structures of cation accommodation in the H-ZSM-5 zeolite. The fine K-point (1 × 1 × 1) and cut-off energy of 550 eV were employed, combined with core–valance interactions described by ultrasoft pseudo potential generated on-the-fly. The ^13^C calculated chemical shifts were further converted to (δ ^13^C) cal values, which were referred to the absolute shielding of TMO, namely 80 ppm for the experimental values.

For the ethene formation pathway calculation, H-ZSM-5 zeolites are represented by a 72T model (HSi71AlO179, 252 atoms), which were extracted from their crystallographic structural data (http://www.iza-structure.org/databases/). The 72T contains the complete double 10-MR intersection pores of H-ZSM-5 zeolite. The terminal Si–H was fixed at a bond length of 1.47 Å, oriented along the direction of the corresponding Si–O bond. The substituted Al atom was placed at the T12 site of the crystallographic position during structural optimization, whereas the proton was located at the O24 site. The previous works have demonstrated that the Si_12_-O_24_(H)-Al_12_ Bronsted acid site located at the channel intersection of H-ZSM-5 zeolite with maximum accessibility for bulky reactants and transition states^17,43,44^. The 22T active site atoms (HSi21AlO25, 48 atoms) and the adsorbed hydrocarbon complex were treated as the high-layer (See Fig. S12) while the rest of the frameworks were treated as the low-layer. To retain the structural integrities of the modeled zeolite, partial structure optimizations of the 72T cluster were performed by relaxing the atoms in the the high-level layer while keeping the rest of atoms fixed at their crystallographic positions. All the TS structures are found by the QST 3 method in the Gaussian program. Then the IRC (Intrinsic Reaction Coordinate) method was used to determine the structures of the corresponding reactant and product. Then based on the imaginary vibrational model of the optimized TS, we adjusted the positions of the vibrational atoms slightly along the calculated reaction coordinate on the two directions toward the reactant and product, respectively, and finally optimized the resulting structures to the minimum structures. These methods have been widely employed in the previous theoretical work.

A combined theoretical approach, namely ONIOM (ωB97xd/6–31 G(d,p): am1) was used for the geometry optimization of adsorption states and transition states (TS). The ωB97XD hybrid density function was developed to consider long-range-corrected hybrid functional, which implicitly accounted for empirical dispersion and could describe long-range dispersion interactions well with respect to the traditional density functional theory methods^42^. This functional was also recently found to perform very well for the description of adsorption and reactions on zeolites. All energies report herein were predicted at the ωB97XD/6–31 G(d, p) level based on the optimized structures.

The harmonic frequency calculations employing a partial Hessian vibrational analysis (PHVA)^45^, including the 22T high layer active acidic sites and organic species were performed to check whether the stationary points found exhibit the proper number of imaginary frequencies. In frequency calculations, besides the atoms in high level and organic fragments, the constraints of the zeolite framework were also kept as the same in geometry optimizations, so one negative frequency would be observed for transition state point and none for the corresponding reactant and product. The Gibbs free energies (*G*) at 423 K (*T*) were calculated from harmonic frequencies.

1 $$G = H - T \times S = \left( {E + \mathrm{ZPVE} + H_{{\mathrm{vib}}} + H_{{\mathrm{trans}}} + H_{{\mathrm{rot}}}} \right) - T \times S.$$

It can be seen that Gibbs free energies (*G*) include contributions from electronic energies (*E*), zero-point vibrational energies (ZPVE), vibrational enthalpies (*H*_vib_), translational (*H*_trans_) and rotational enthalpies (*H*_rot_) for reactant molecules and the effect of entropy energy (*T* × *S*), which can more accurately describe the mechanism of ethanol dehydration reaction.

And then, the reaction rate constants (*k*) at 473 K were further obtained by transition state theory (TST):

2 $$k = A.\exp \left( { - \frac{{\Delta H_{{\mathrm{act}}}}}{{{RT}}}} \right) = \frac{{k_{\mathrm{B}}T}}{h}.\exp \left( {\frac{{\Delta S_{{\mathrm{act}}}}}{R}} \right).\exp \left( { - \frac{{\Delta H_{{\mathrm{act}}}}}{{RT}}} \right) = \frac{{k_{\mathrm{B}}T}}{h}.\exp \left( { - \frac{{\Delta G_{{\mathrm{act}}}}}{{RT}}} \right),$$

where *A* is pre-exponential factor, *k*_B_ is Boltzmann’s constant, *h* is Planck’s constant and *T* is the reaction temperature. All the geometry optimizations and frequency calculations were performed using the Gaussian 09 package^46^.

## Supplementary information

Supplementary Information

## Data Availability

All the data supporting the findings of this study are available within the article and its supplementary information file or from the corresponding author upon reasonable request.

## References

[1] Alvira P, Tomás-Pejó E, Ballesteros M, Negro MJ (2010). Pretreatment technologies for an efficient bioethanol production process based on enzymatic hydrolysis: a review. Bioresour. Technol..

[2] Sun J, Wang Y (2014). Recent advances in catalytic conversion of ethanol to chemicals. ACS Catal..

[3] Zhang M, Yu Y (2013). Dehydration of ethanol to ethylene. Ind. Eng. Chem. Res..

[4] Diaz Alvarado FA, Gracia F (2010). Steam reforming of ethanol for hydrogen production: Thermodynamic analysis including different carbon deposits representation. Chem. Eng. J..

[5] Bi JD, Guo XW, Liu M, Wang XS (2010). High effective dehydration of bio-ethanol into ethylene over nanoscale HZSM-5 zeolite catalysts. Catal. Today.

[6] Roy S (2012). Mechanistic study of alcohol dehydration on gamma-Al2O3. ACS Catal..

[7] Janik MJ, Macht J, Iglesia E, Neurock M (2009). Correlating acid properties and catalytic function: a first-principles analysis of alcohol dehydration pathways on polyoxometalates. J. Phys. Chem. C..

[8] Sun J (2011). Direct conversion of bio-ethanol to isobutene on nanosized ZnxZryOz mixed oxides with balanced acid–base sites. J. Am. Chem. Soc..

[9] Phillips CB, Datta R (1997). Production of ethylene from hydrous ethanol on h-zsm-5 under mild conditions. Ind. Eng. Chem. Res..

[10] Van der Borght K (2016). Insights into the reaction mechanism of ethanol conversion into hydrocarbons on H-ZSM-5. Angew. Chem. Int. Ed..

[11] Hemelsoet K, Van der Mynsbrugge J, De Wispelaere K, Waroquier M, Van Speybroeck V (2013). Unraveling the reaction mechanisms governing methanol-to-olefins catalysis by theory and experiment. ChemPhysChem.

[12] Olsbye U (2012). Conversion of methanol to hydrocarbons: how zeolite cavity and pore size controls product selectivity. Angew. Chem. Int. Ed..

[13] Chiang H, Bhan A (2010). Catalytic consequences of hydroxyl group location on the rate and mechanism of parallel dehydration reactions of ethanol over acidic zeolites. J. Catal..

[14] Phung TK, Busca G (2015). Diethyl ether cracking and ethanol dehydration: Acid catalysis and reaction paths. Chem. Eng. J..

[15] Gayubo AG, Tarrío AM, Aguayo AT, Olazar M, Bilbao J (2001). Kinetic modelling of the transformation of aqueous ethanol into hydrocarbons on a HZSM-5 Zeolite. Ind. Eng. Chem. Res..

[16] Kondo JN (2010). Activation energies for the reaction of ethoxy species to ethene over zeolites. J. Phys. Chem. C..

[17] Alexopoulos K (2016). DFT-based microkinetic modeling of ethanol dehydration in H-ZSM-5. J. Catal..

[18] Kim S, Robichaud DJ, Beckham GT, Paton RS, Nimlos MR (2015). Ethanol dehydration in HZSM-5 studied by density functional theory: evidence for a concerted process. J. Phys. Chem. A.

[19] Xin H (2014). Catalytic dehydration of ethanol over post-treated ZSM-5 zeolites. J. Catal..

[20] Wang W, Jiao J, Jiang YJ, Ray SS, Hunger M (2005). Formation and decomposition of surface ethoxy species on acidic zeolite Y. Chemphyschem.

[21] Kondo JN, Ito K, Yoda E, Wakabayashi F, Domen K (2005). An ethoxy intermediate in ethanol dehydration on bronsted acid sites in zeolite. J. Phys. Chem. B.

[22] Zecchina A (1996). IR spectroscopy of neutral and ionic hydrogen-bonded complexes formed upon interaction of CH_3_OH, C_2_H_5_OH, (CH_3_)_2_O, (C_2_H_5_)_2_O and C_4_H_8_O with H-Y, H-ZSM-5 and H-mordenite: comparison with analogous adducts formed on the H-Nafion superacidic membrane. J. Chem. Soc., Faraday Trans..

[23] Lee CC, Gorte RJ, Farneth WE (1997). Calorimetric study of alcohol and nitrile adsorption complexes in H-ZSM-5. J. Phys. Chem. B.

[24] Nguyen TM, Le Van Mao R (1990). Conversion of ethanol in aqueous solution over ZSM-5 zeolites: Study of the reaction network. Appl. Catal..

[25] Goguen PW (1998). Pulse-quench catalytic reactor studies reveal a carbon-pool mechanism in methanol-to-gasoline chemistry on zeolite HZSM-5. J. Am. Chem. Soc..

[26] Stepanov AG, Luzgin MV, Romannikov VN, Sidelnikov VN, Paukshtis EA (1998). The Nature, structure, and composition of adsorbed hydrocarbon products of ambient temperature oligomerization of ethylene on acidic zeolite H-ZSM-5. J. Catal..

[27] Munson EJ, Kheir AA, Lazo ND, Haw JF (1992). In situ solid-state NMR study of methanol-to-gasoline chemistry in zeolite HZSM-5. J. Phys. Chem..

[28] Munson EJ, Haw JF (1991). Nmr observation of trimethyloxonium formation from dimethyl ether on zeolite HZSM-5. J. Am. Chem. Soc..

[29] Lesage A, Bardet M, Emsley L (1999). Through-bond carbon-carbon connectivities in disordered solids by NMR. J. Am. Chem. Soc..

[30] Wang C (2014). New insight into the hydrocarbon-pool chemistry of the methanol-to-olefins conversion over zeolite H-ZSM-5 from GC-MS, solid-state nmr spectroscopy, and DFT calculations. Chem. Eur. J..

[31] Wang C (2015). Experimental evidence on the formation of ethene through carbocations in methanol conversion over H-ZSM-5 zeolite. Chem. Eur. J..

[32] Wang C (2016). Direct detection of supramolecular reaction centers in the methanol-to-olefins conversion over zeolite H-ZSM-5 by ^13^C-^27^Al solid-state NMR spectroscopy. Angew. Chem. Int. Ed..

[33] Svelle S (2006). Conversion of methanol into hydrocarbons over zeolite H-ZSM-5: Ethene formation is mechanistically separated from the formation of higher alkenes. J. Am. Chem. Soc..

[34] Sun X (2014). On reaction pathways in the conversion of methanol to hydrocarbons on HZSM-5. J. Catal..

[35] Munson EJ, Kheir AA, Lazo ND, Haw JF (1992). Insitu solid-state NMR-study of methanol-to-gasoline chemistry in zeolite HZSM-5. J. Phys. Chem..

[36] Munson EJ, Kheir AA, Haw JF (1993). An in-situ solid-state nmr-study of the formation and reactivity of trialkylonium ions in zeolites. J. Phys. Chem..

[37] Olah, G. A., Laali, K. K., Wang, Q. & Prakash, G. S. *Onium Ions* (John Wiley & Sons, New York, 1998).

[38] Kondo JN, Yamazaki H, Osuga R, Yokoi T, Tatsumi T (2015). Mechanism of decomposition of surface ethoxy species to ethene and acidic OH groups on H-ZSM-5. J. Phys. Chem. Lett..

[39] Olah GA (1984). Onium Ylide chemistry. 1. Bifunctional acid-base-catalyzed conversion of heterosubstituted methanes into ethylene and derived hydrocarbons. The onium ylide mechanism of the C1.fwdarw. C2 conversion. J. Am. Chem. Soc..

[40] Fung BM, Khitrin AK, Ermolaev K (2000). An Improved broadband decoupling sequence for liquid crystals and solids. J. Magn. Reson..

[41] Clark Stewart J (2005). First principles methods using CASTEP. Z. Krist. Cryst. Mater..

[42] Chai JD, Head-Gordon M (2008). Long-range corrected hybrid density functionals with damped atom-atom dispersion corrections. Phys. Chem. Chem. Phys..

[43] Brändle M, Sauer J (1998). Acidity differences between inorganic solids induced by their framework structure. a combined quantum mechanics/molecular mechanics ab initio study on zeolites. J. Am. Chem. Soc..

[44] Svelle S, Tuma C, Rozanska X, Kerber T, Sauer J (2009). Quantum chemical modeling of zeolite-catalyzed methylation reactions: toward chemical accuracy for barriers. J. Am. Chem. Soc..

[45] Ghysels, A., Van Neck, D., Van Speybroeck, V., Verstraelen, T. & Waroquier, M. Vibrational modes in partially optimized molecular systems. *J. Chem. Phys*. **126**, 224102 (2007). 10.1063/1.2737444 17581039

[46] Frisch, M. J. et al. Gaussian 09. Gaussian Inc., Wallingford, CT, 2010.

