# Supplementary material for: Observation of an oxonium ion intermediate in ethanol dehydration to ethene on zeolite
Source: Nat Commun. 2019 Apr 29;10:1961. doi: 10.1038/s41467-019-09956-7 (PMC6488627; doi:10.1038/s41467-019-09956-7)
Supplement: Supplementary file 1 — Supplementary Information [file 41467_2019_9956_MOESM1_ESM.pdf]

Supplementary Information

Observation of An Oxonium Ion Intermediate in Ethanol Dehydration to Ethene on Zeolite

Zhou et al.

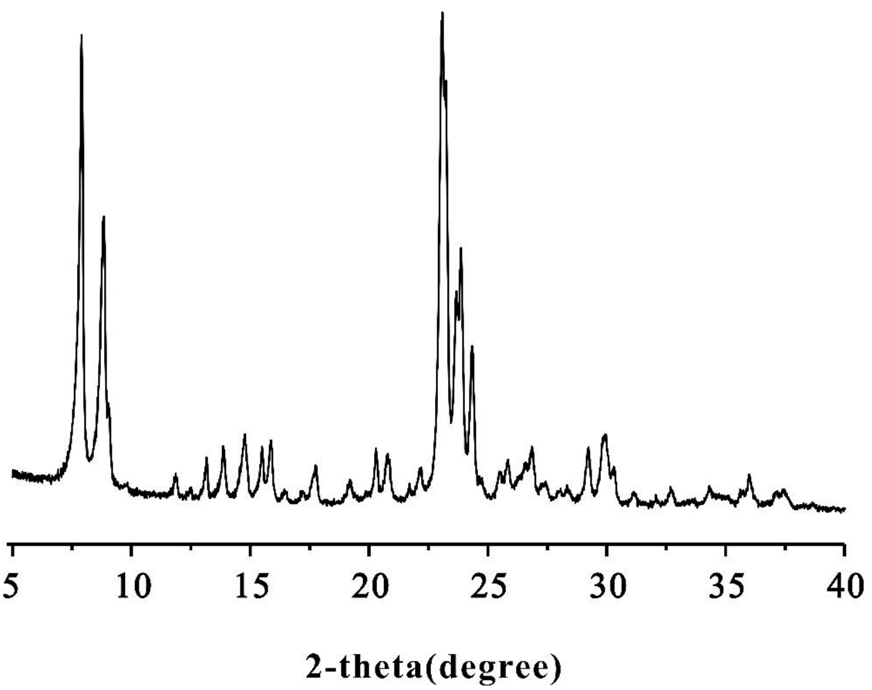

Supplementary Figure 1. XRD pattern of H-ZSM-5 zeolite.

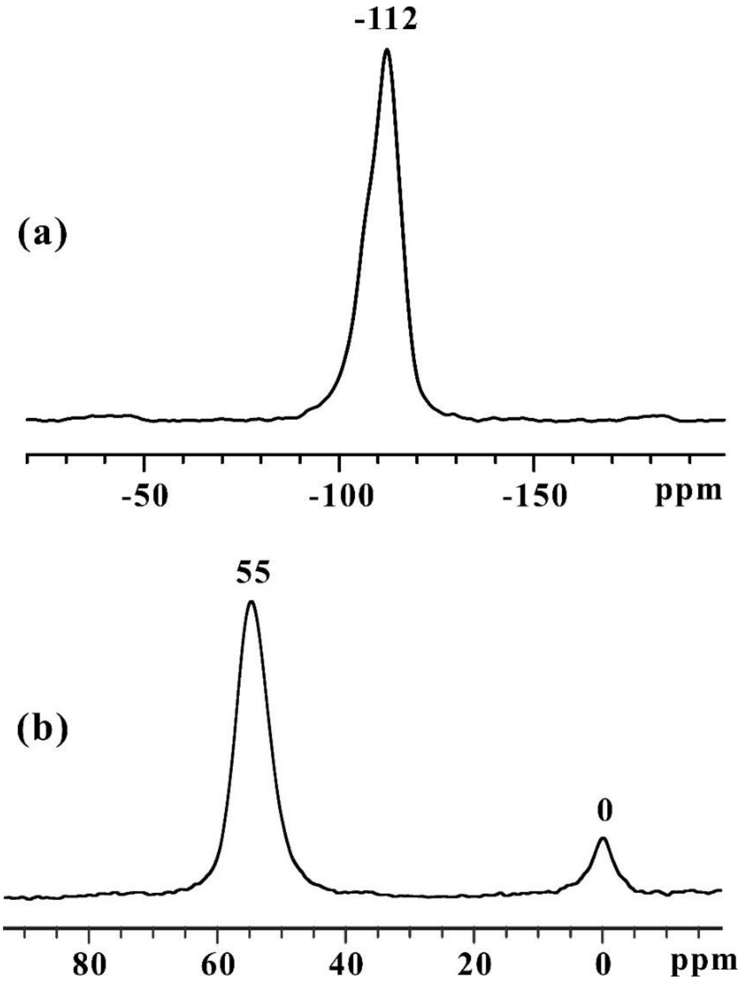

Supplementary Figure 2. ^{29}Si (a) and ^{27}Al (b) MAS NMR spectra of H-ZSM-5 zeolite. The signals at 55 ppm and 0 ppm on spectrum (b) are ascribed to framework tetrahedral Al and extra-framework octahedral Al respectively.

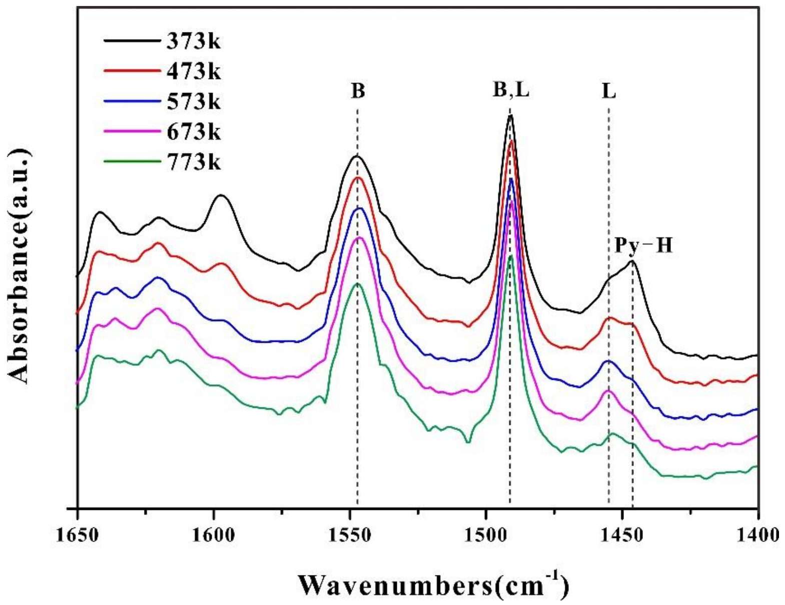

Supplementary Figure 3. FT-IR spectra of pyridine adsorbed on H-ZSM-5 after evacuation at different temperatures. The B, L, and Py-H peaks represent Brønsted acid sites, Lewis acid sites and hydrogen bonded pyridine, respectively. The amount of Brønsted acid site and Lewis acid site (extra-framework alumina) is determined to be 1.03 mmol.g^{-1} and 0.08 mmol.g^{-1} respectively.

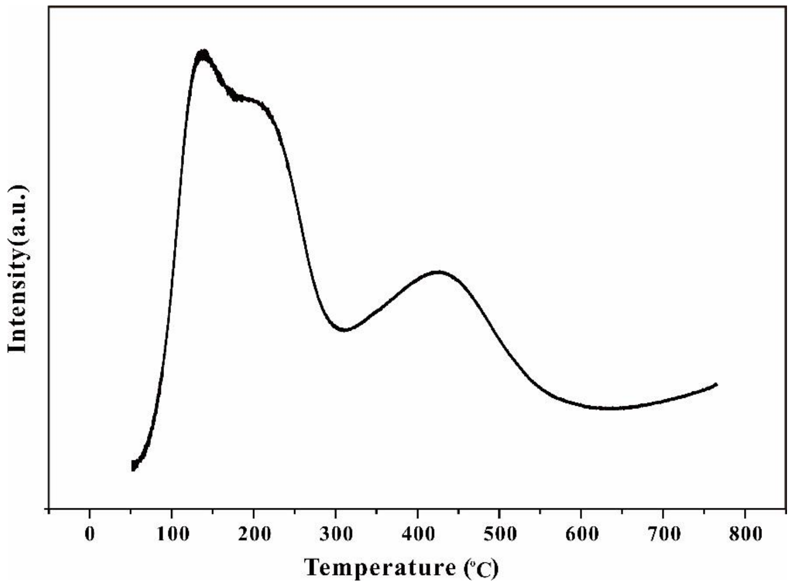

Supplementary Figure 4. NH_3-TPD profile of H-ZSM-5 zeolite. Three different NH_3 desorption temperature features (100-200, 200-320 and 350-550°C) indicate three kinds of acid sites with different acid strength, namely weak, medium and strong acid strengths respectively.

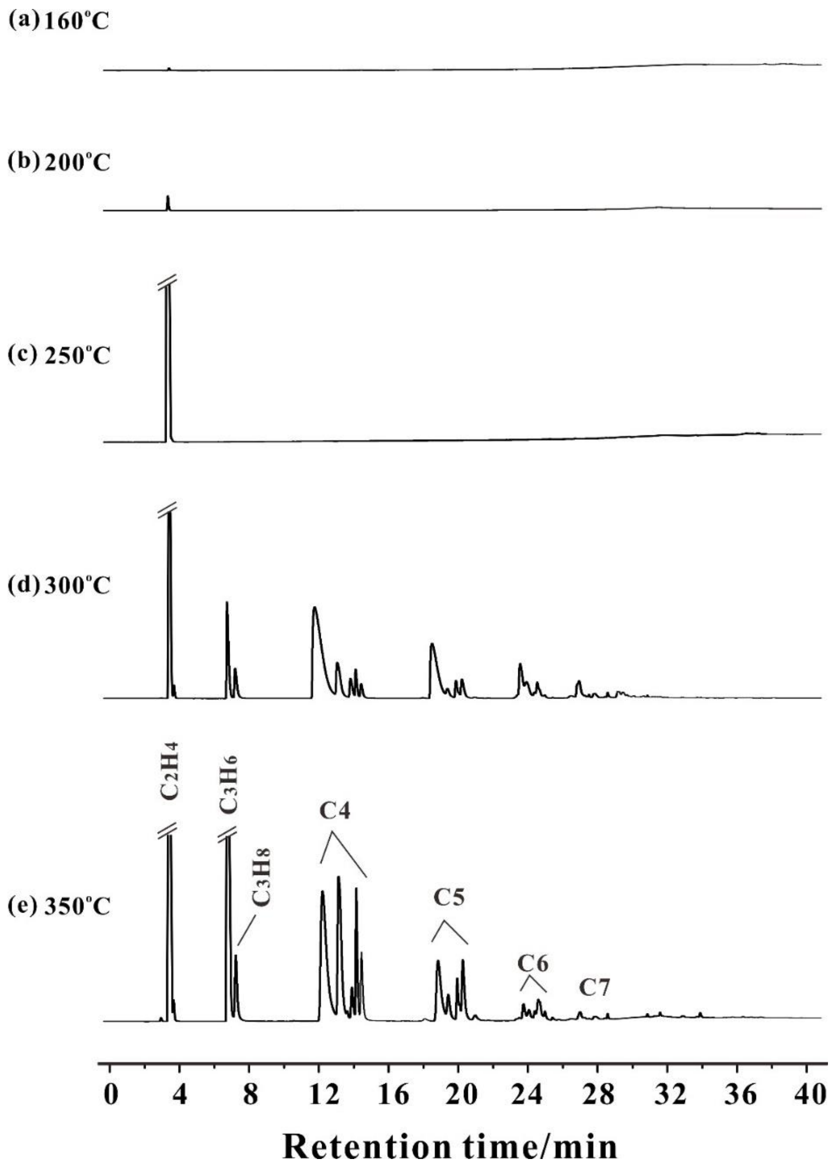

Supplementary Figure 5. GC analysis of effluent products obtained from pulsing ethanol reaction over H-ZSM-5 for 4 s at different temperatures.

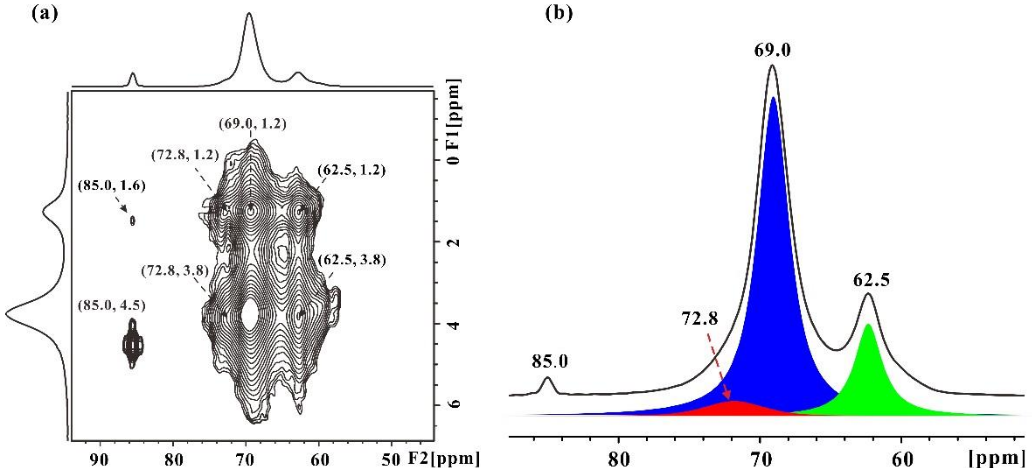

Supplementary Figure 6. 2D ^{1}H-^{13}C HETCOR MAS NMR spectrum (a) and signal deconvolution of the ^{13}C CP MAS NMR spectrum (b) of trapped species on H-ZSM-5 obtained from reaction of $CH_3{}^{13}CH_2OH$ at 200 °C for 4 s. The 72.8 ppm signal is due to the methylene carbon of ethoxy species.

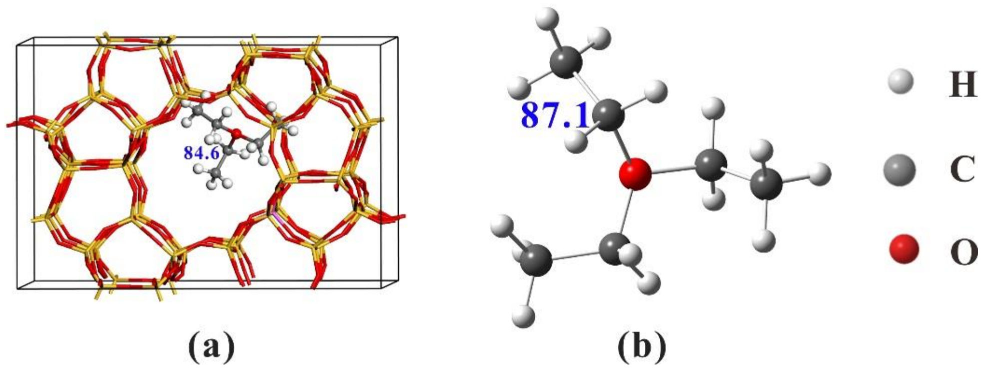

Supplementary Figure 7. Theoretically optimized structure of triethyloxonium ion (TEO) in ZSM-5 channel (a) and in gas state (b). The predicted ^{13}C chemical shift (in ppm) is indicated for one of the three methylene carbon atoms.

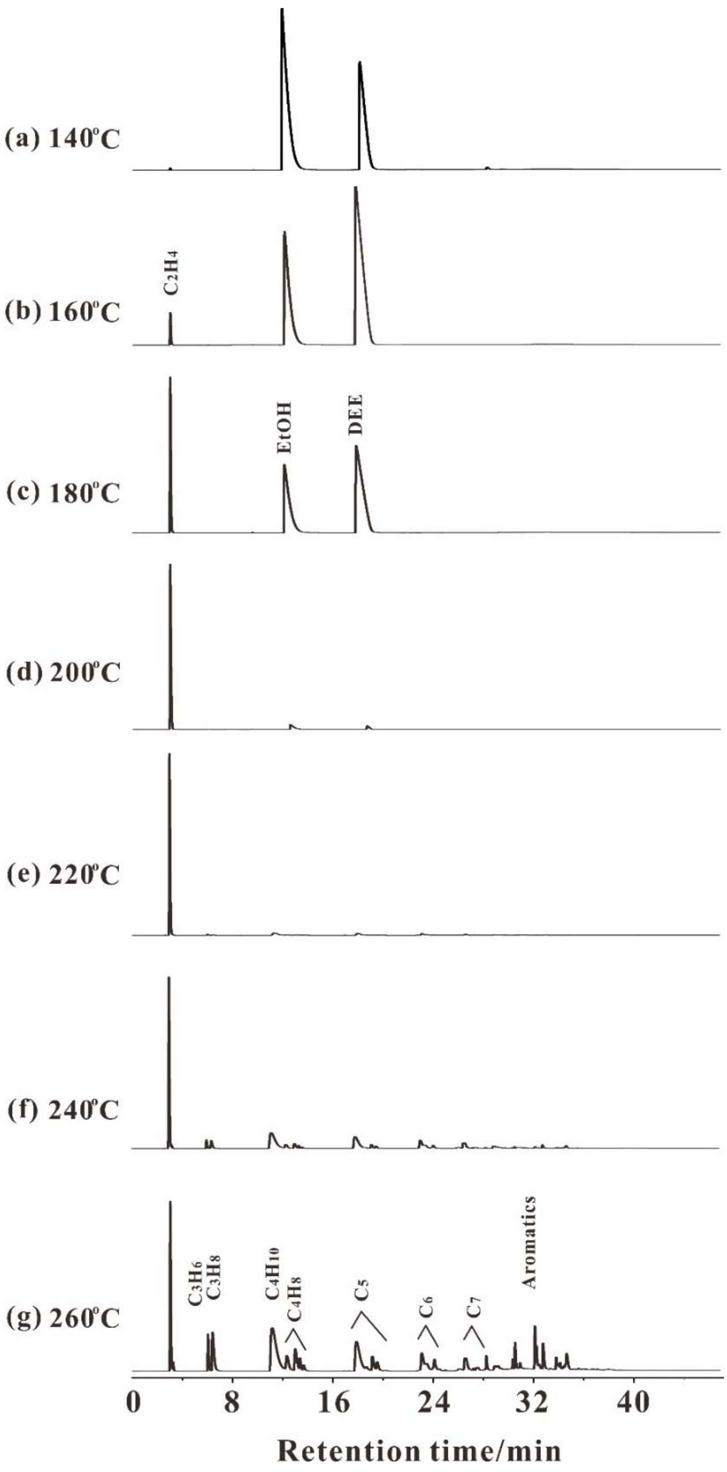

Supplementary Figure 8. GC analysis of effluent products obtained from continue-flow reaction of ethanol over H-ZSM-5 for 10 min at different temperatures.

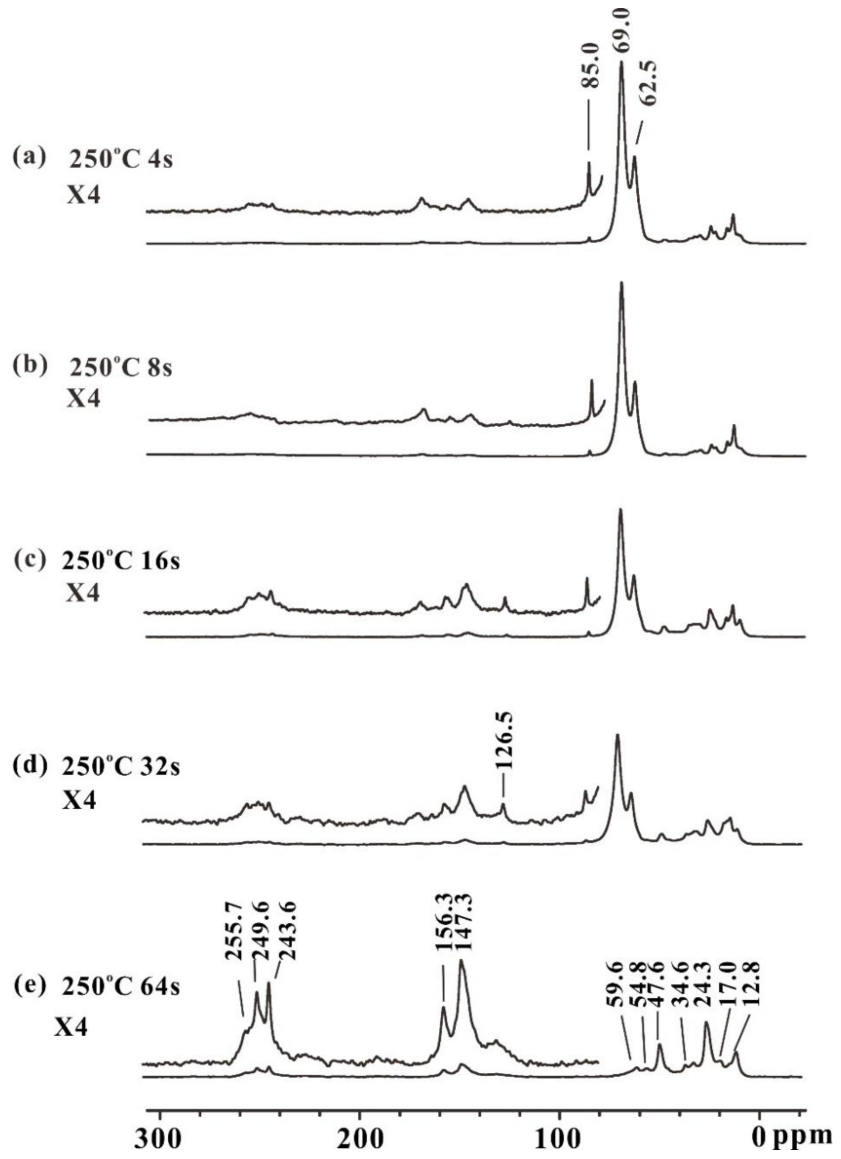

Supplementary Figure 9. ^{13}C CP MAS NMR spectra of trapped species on H-ZSM-5 obtained from pulse-quench reactions of $CH_3{}^{13}CH_2OH$ at 250 °C for different time.

Supplementary Table 1. Integrated ^{13}C signal intensity of TEO (85.0 ppm) and ethoxy species (72.8 ppm) in the reaction. The intensity is normalized to that of 85.0 ppm signal at reaction time of 4s.

Sample	85.0 ppm	72.8 ppm
4s	1	18.94
8s	0.93	16.84
16s	0.52	8.23
32s	0.36	4.57

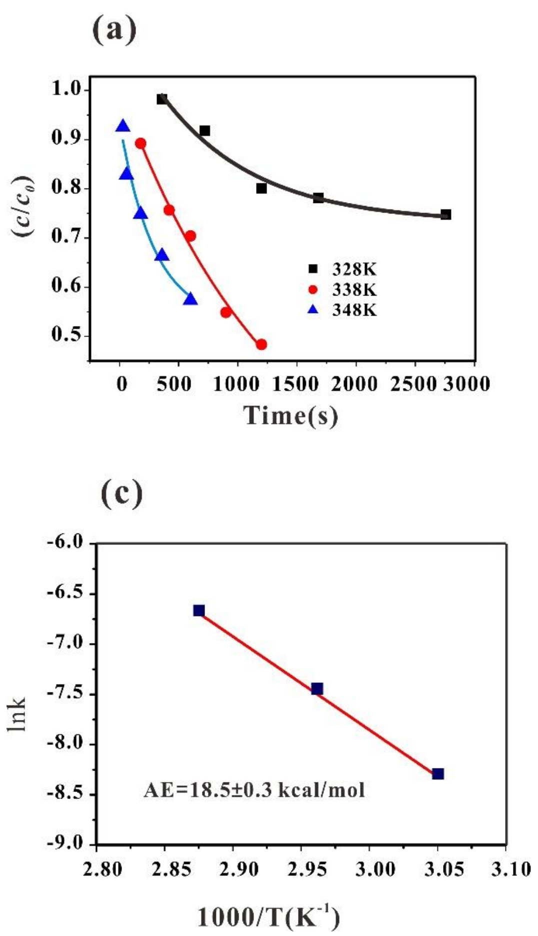

Supplementary Figure 10. The normalized concentration of TEO determined by ^{13}C MAS NMR as a function of reaction time at different temperatures (a), the analysis of initial reaction rate constants (at low TEO conversion) at different temperatures (b), and Arrhenius plot for the measurement of the activation energy of formation of ethoxy species from TEO by ^{13}C MAS NMR (c). Assuming that the TEO conversion follows the first order reaction rate equation: $\ln(c/c_0) = -kt + A$, in which c_0 is the initial reactant concentration, t is reaction time, c is the reactant concentration at reaction time t, k is the reaction rate constant and A is the constant.

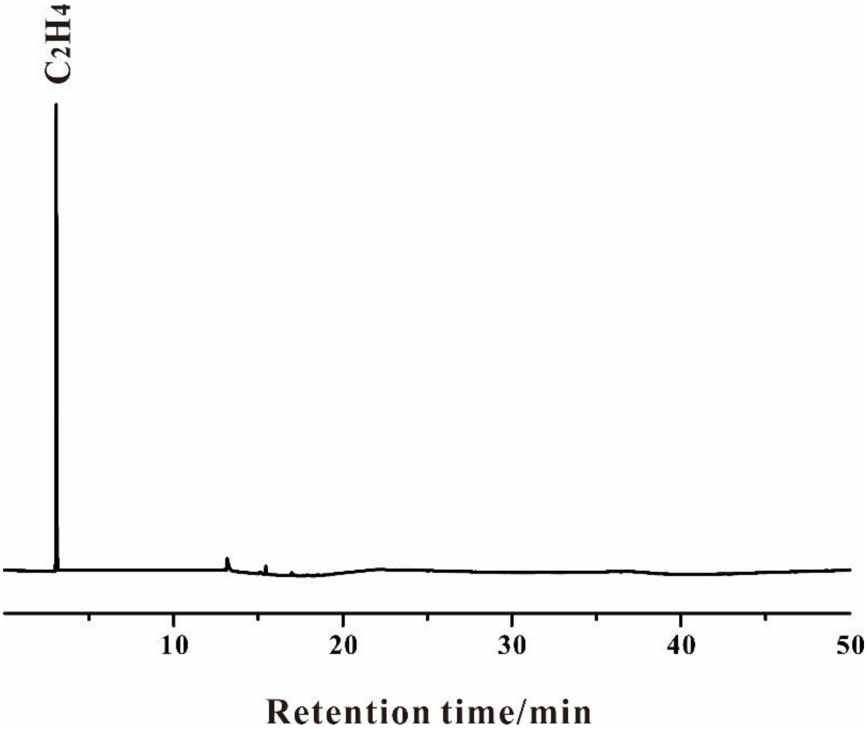

Supplementary Figure 11. GC analysis of the effluent product obtained by heating TEO-ZSM-5 at 200 ºC for 5min.

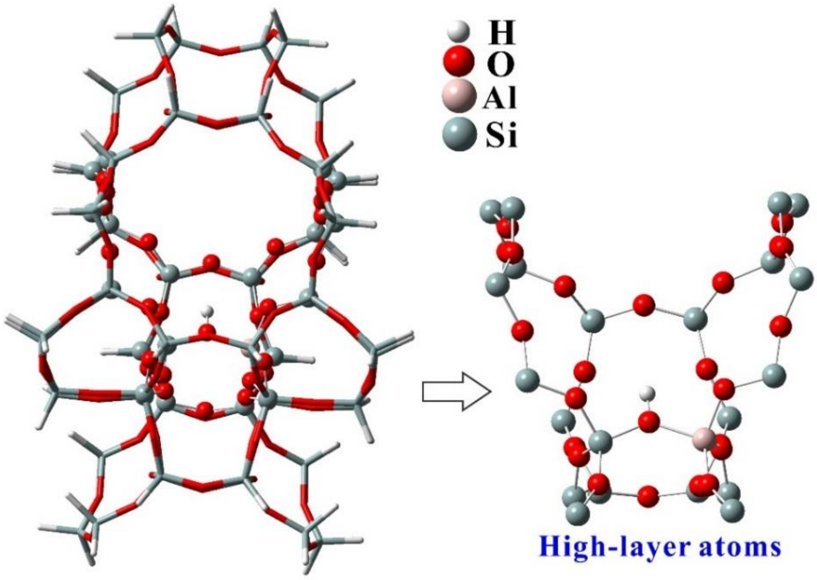

Supplementary Figure 12. Representation of H-ZSM-5 zeolite by a 72 T model (HSi71AlO179, 252 atoms). The 22T active site atoms (HSi21AlO25, 48 atoms) and the adsorbed hydrocarbon complex were treated as the high-layer.

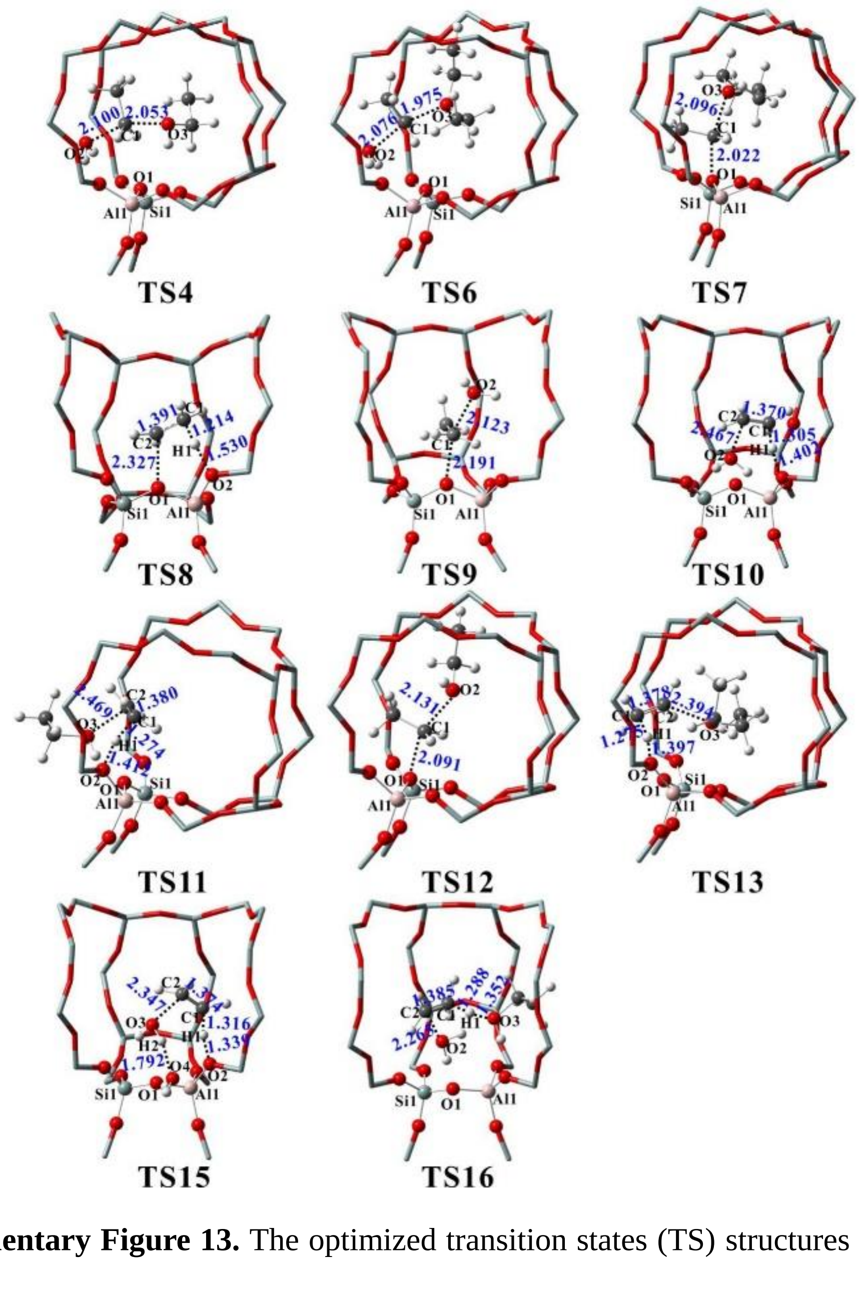

Supplementary Figure 13. The optimized transition states (TS) structures for ethene formation through various elementary steps shown in Figure 5. The main geometric parameters are labelled (in Å) for each structure. The XYZ coordinates for all the transition states in Supplementary Table 3.

Supplementary Table 2. Adsorption energy (ads, kJ mol^{-1}), reaction energy (react, J mol^{-1} K^{-1}), and energy barrier for (act, kJ mol^{-1}) the elementary steps (numbered as indicated in Figure 5). Δ*H* represents the enthalpy; Δ*S* represents the entropy; Δ*E* represents the electronic energy; Δ*G* represents the Gibbs free energy at 473 K; $_g$ represents gas stage.

	Elementary steps	ΔH_{ads}	ΔH_{react}	ΔH_{act}	ΔS_{ads}	ΔS_{react}	ΔS_{act}	ΔE_{ads}	ΔE_{react}	ΔE_{act}	ΔG_{ads}	ΔG_{react}	ΔG_{act}
(1)	$ZeOH+EtOH_{(g)} \leftrightarrow A$	-129.5			-184.3			-133.5			-42.3		
(2)	$A+EtOH_{(g)} \leftrightarrow B$	-139.0			-166.2			-129.4			-60.4		
(3)	$B \leftrightarrow C$	43.4			-6.1			27.0			46.3		
(4)	$C \overset{TS4}{\longleftrightarrow} DEE.H_2O^*$		42.3	118.3		-15.6	-22.7		39.4	119.2		49.6	129.0
(5)	$DEE^*+EtOH_{(g)} \leftrightarrow D$	-73.4			-194.1			-85.0			18.4		
(6)	$D \overset{TS6}{\longleftrightarrow} TEO.H_2O^*$		39.4	94.9		21.8	1.6		38.6	97.7		29.1	94.1
(7)	$TEO^* \overset{TS7}{\longleftrightarrow} Ethoxy.DEE^*$		16.3	73.6		2.7	-16.9		14.1	78.1		15.0	81.7
(8)	$Ethoxy \overset{TS8}{\longleftrightarrow} Ethene^*$		48.9	108.7		35.2	2.4		56.0	127.6		32.3	107.6
(9)	$A \overset{TS9}{\longleftrightarrow} Ethoxy.H_2O^*$		86.9	153.9		22	16		87	161.3		76.5	146.3
(10)	$A \overset{TS10}{\longleftrightarrow} Ethene.H_2O^*$		97.1	177.6		60.5	12.2		101.2	197.9		68.5	171.8

(11)	$DEE^* \overset{TS11}{\longleftrightarrow} Ethene.EtOH^*$		98	145.3		44.1	19.5		96.6	158.9		77.2	136.1
(12)	$DEE^* \overset{TS12}{\longleftrightarrow} Ethoxy.EtOH^*$		88	154.6		21.9	-2.7		83.7	156.4		77.7	155.9
(13)	$TEO^* \overset{TS13}{\longleftrightarrow} Ethene.DEE^*$		79.9	130.9		64.7	6.8		89.3	153.4		49.3	127.7
(14)	$A + H_2O_{(g)} \leftrightarrow E$	-112.1			-140.9			-118.6			-45.5		
(15)	$E \overset{TS15}{\longleftrightarrow} Ethene.2H_2O^*$		145.6	194.4		54.1	20.4		148.9	212.5		120.1	184.8
(16)	$B \overset{TS16}{\longleftrightarrow} Ethene.H_2O.EtOH^*$		57.3	151.9		66.1	14.1		64	167.6		26	145.2

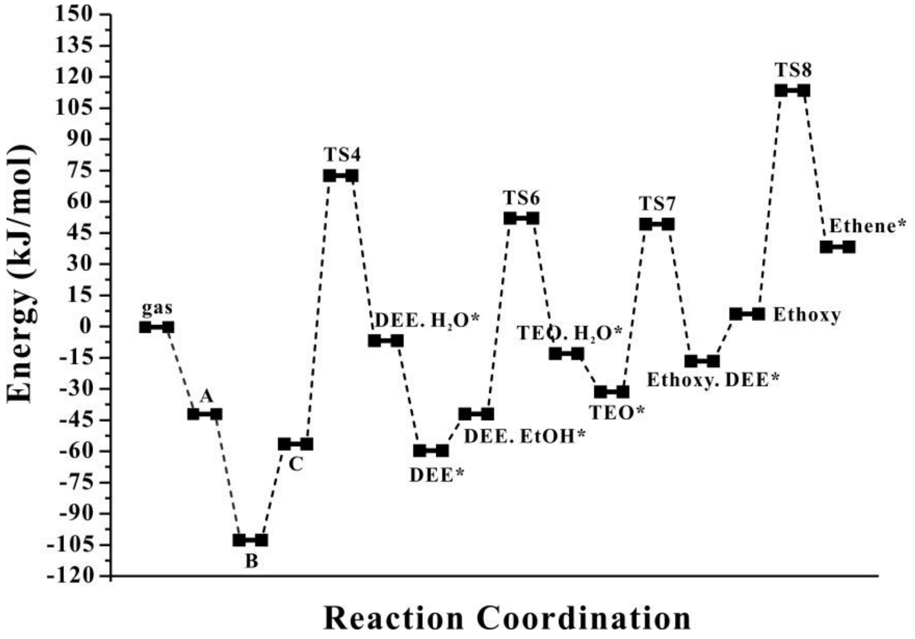

Supplementary Figure 14. The reaction Gibbs free energy profile of ethene formation through route 1 over H-ZSM-5 zeolite at 473 K.

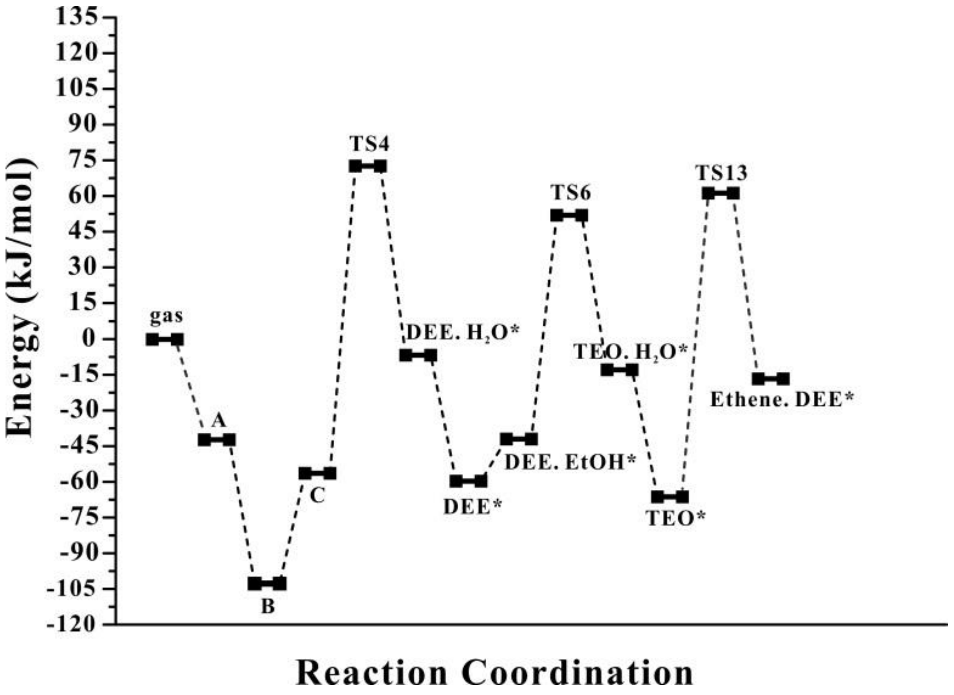

Supplementary Figure 15. The reaction Gibbs free energy profile of ethene formation through route 2 over H-ZSM-5 zeolite at 473 K.

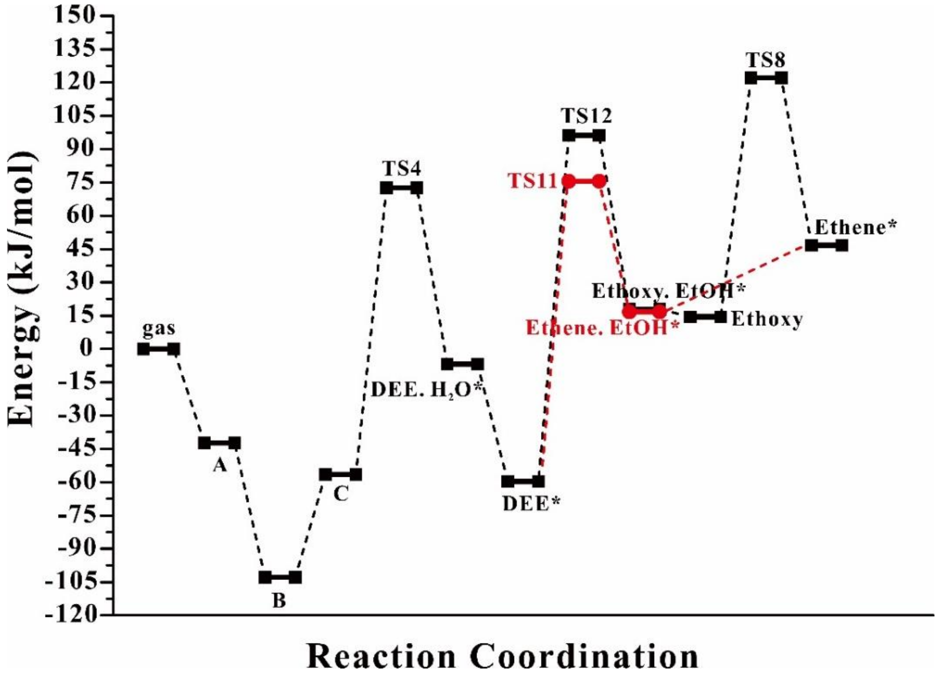

Supplementary Figure 16. The reaction Gibbs free energy profile of ethene formation through route 3 (■) and route 4 (●) over H-ZSM-5 zeolite at 473 K.

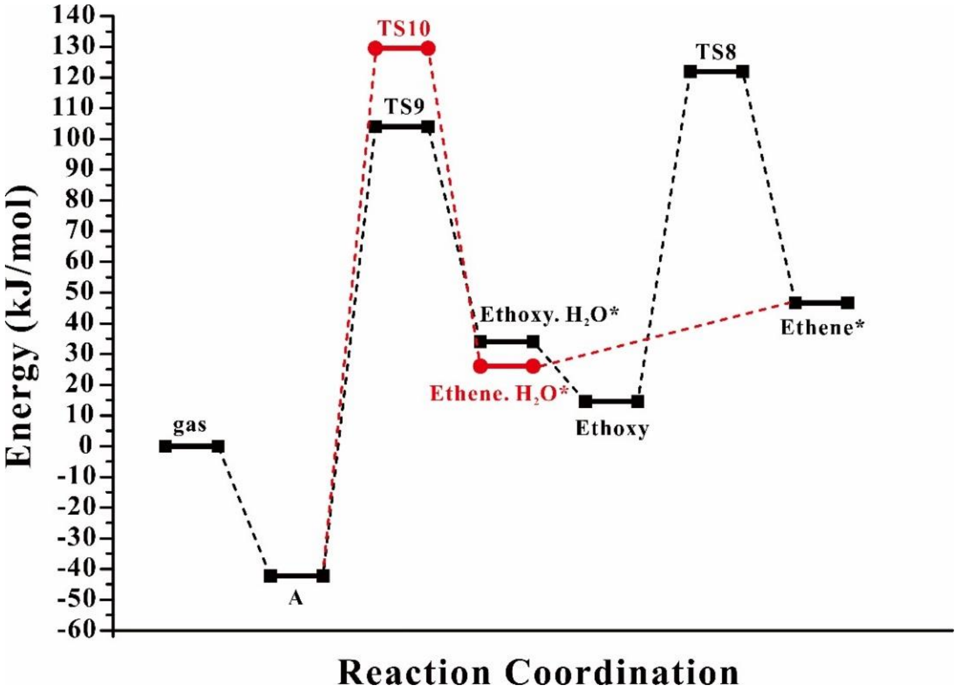

Supplementary Figure 17. The reaction Gibbs free energy profile of ethene formation through route 5 (■) and route 6 (●) over H-ZSM-5 zeolite at 473 K.

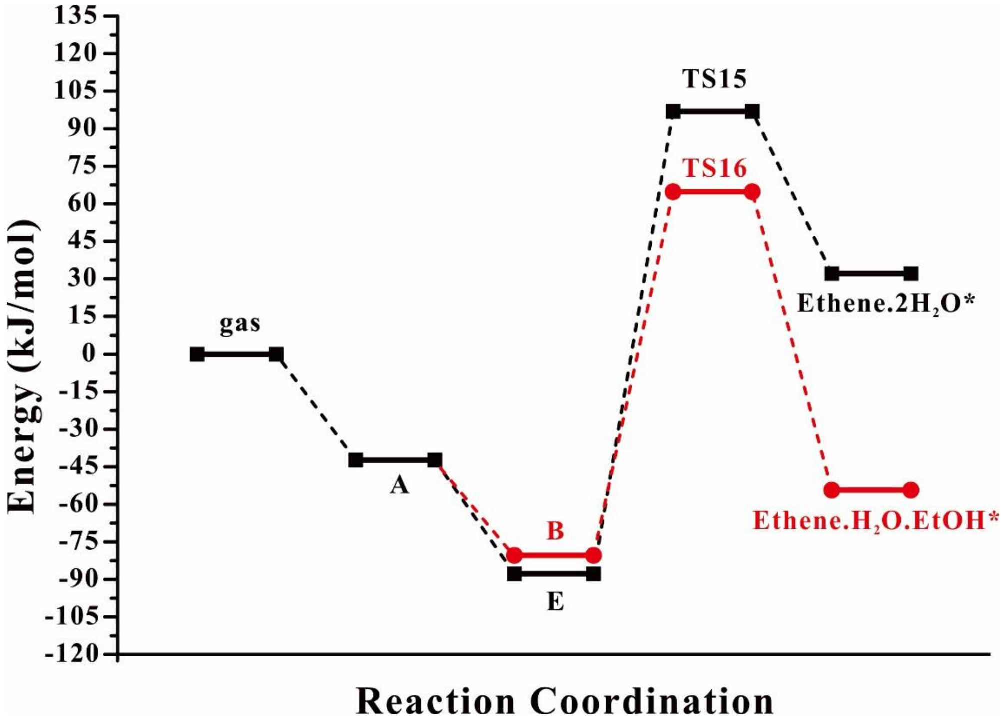

Supplementary Figure 18. The reaction Gibbs free energy profile of ethene formation through route 7 (■) and route 8 (●) over H-ZSM-5 zeolite at 473 K.

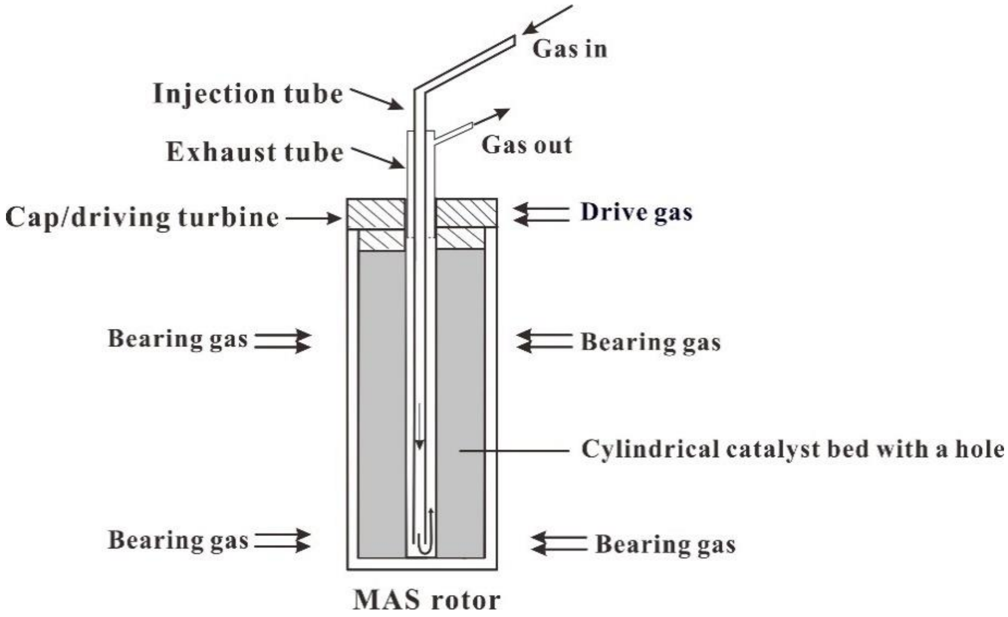

Supplementary Figure 19. Schematic of the MAS rotor used as the reactor for in situ NMR.

Supplementary Table 3

TS4

O	-4.30584000	3.33432900	1.76286100
O	-3.24770100	2.48707800	3.94308000
O	-7.35632200	-1.90032300	1.87531000
O	-7.72476200	0.63640200	1.88953400
O	-6.70476800	-0.49377400	3.96482700
O	-4.21754200	-3.37877600	3.80618400
O	-3.11397500	-1.39307300	4.99526200
O	-1.23068000	-5.69114100	3.88770400
O	-4.21640600	-3.38962400	-3.78726200
O	-3.11212300	-1.40777100	-4.98179600
O	-1.22934000	-5.70238100	-3.86098200
Si	-1.42304100	-7.26580300	3.81716000
Si	-1.42172700	-7.27683300	-3.78593400
O	2.66297700	-5.00240000	2.44867900
O	-2.97182200	-7.63094700	3.91412200
O	-0.92064800	-7.81546400	2.40834000
O	2.66381900	-5.00946700	-2.42261500
O	-2.97047500	-7.64225500	-3.88237100
O	-0.91982200	-7.82240200	-2.37535300
Si	2.77441400	-6.30406400	1.54518600
Si	0.35705000	-8.24899200	1.55379200
Si	2.77494400	-6.30850500	-1.51531100
Si	0.35758100	-8.25344700	-1.51910600
O	1.58048800	-7.29232600	1.89861600
O	4.14184000	-7.04563400	1.77930000
O	2.69781500	-5.82861600	0.01418400
O	-0.06725800	-8.13414700	0.01705000
O	1.58113900	-7.29778600	-1.86628200
O	4.14245100	-7.05074800	-1.74679700
O	-4.30526200	3.32910900	-1.76336600
O	-3.24629700	2.47569600	-3.94074600
O	-7.35565400	-1.90574200	-1.86174300
O	-7.72417400	0.63094800	-1.88340300
O	-6.70345300	-0.50526400	-3.95507200
Si	-3.67016500	-0.48296700	-6.14257900
Si	-3.08761000	2.45383400	-5.51523800
Si	-6.71863000	-0.56663200	-5.52138600
O	-2.99794100	0.95946100	-6.04785900
O	-5.25097600	-0.35140700	-6.07024800
O	-7.27097300	-1.96423200	-6.03045300
Si	-3.08951400	2.46985100	5.51764800

Si	-6.72055600	-0.55055400	5.53130600
Si	-3.67230700	-0.46525800	6.15340300
O	-3.00008500	0.97701300	6.05464000
O	-7.27312600	-1.94668900	6.04423100
O	-5.25306200	-0.33383300	6.08003900
Si	-5.67325100	4.07576900	1.52865100
Si	-8.09059900	2.13087300	1.53726500
Si	-5.67270400	4.07137200	-1.53182600
Si	-8.09006700	2.12641800	-1.53562300
O	-6.86715700	3.08754100	1.88210200
O	-5.78466800	5.37747000	2.43215700
O	-5.74983400	4.55125400	-0.00233600
O	-8.51491200	2.24571700	0.00053600
O	-6.86651200	3.08207600	-1.88279900
O	-5.78383300	5.37040100	-2.43912900
Si	-7.34848400	-3.44717100	1.55121600
Si	-7.34793900	-3.45164100	-1.53309500
O	-6.21085400	-4.18890300	2.37037200
O	-7.06806200	-3.68415200	0.00938400
O	-6.21003300	-4.19575900	-2.34972700
Si	-5.41880600	-4.39445800	3.73229200
O	-4.86227000	-5.87923000	3.77195300
O	-6.40087200	-4.16166500	4.95120200
Si	-7.27077800	-3.52843600	6.10823000
Si	-4.41618200	-7.23906200	4.44810400
Si	-5.41747200	-4.40531400	-3.71077600
O	-4.86096700	-5.89013300	-3.74594500
O	-6.39916900	-4.17599300	-4.93069100
Si	-7.26867200	-3.54613200	-6.08985800
Si	-4.41465100	-7.25192200	-4.41798600
O	5.67985200	-2.97294200	3.76955800
O	5.68115000	-2.98384700	-3.74833400
O	6.60147200	0.54495500	3.88001500
O	6.60281100	0.53371600	-3.86867700
O	5.60776000	3.61893800	1.88348200
O	5.60841100	3.61347300	-1.88141500
O	2.59171900	5.84031500	1.80013600
O	2.59234200	5.83508300	-1.80555900
O	-1.38445000	5.74044300	2.35679900
O	-1.38363800	5.73359700	-2.36329600
Si	-6.14039200	6.01543100	3.84597700
Si	-6.13906000	6.00426300	-3.85491500
Si	-1.42698500	6.56036400	3.71710100
Si	-1.42570000	6.54956700	-3.72599300

Si	6.81847800	4.58656900	1.53008700
Si	6.81900600	4.58212700	-1.53040900
Si	2.83689200	7.37024700	1.52196000
Si	2.83741800	7.36581500	-1.53173500
O	-5.36406100	7.40634200	3.96949300
O	-5.36268700	7.39480700	-3.98219700
O	-0.18858600	7.53031700	3.79110800
O	-2.76780000	7.40692500	3.75304100
O	-0.18727600	7.51930200	-3.80238500
O	-2.76650100	7.39601700	-3.76484900
Si	-4.00973100	8.12120100	4.42589200
Si	-4.00819800	8.10833900	-4.44019800
O	4.34810700	7.73911200	1.82948500
O	1.86987800	8.30446800	2.39933800
O	2.52518600	7.70889900	-0.00548500
O	4.34874500	7.73379000	-1.83981000
O	1.87071000	8.29749000	-2.41215500
Si	1.04841600	8.49740100	3.74871800
Si	1.04971000	8.48650500	-3.76237100
O	6.36974800	6.07655900	1.76064700
O	8.06896100	4.21479600	2.43624400
O	7.27084200	4.41206200	0.00011600
O	6.37035600	6.07144100	-1.76544800
O	8.06980200	4.20773000	-2.43505200
Si	5.80782100	7.41984900	2.37610200
Si	5.80864200	7.41294100	-2.38499300
Si	8.61917500	3.73962000	3.85182800
Si	8.62050900	3.72845000	-3.84906300
Si	8.06893200	0.76178300	4.42875300
Si	8.07046000	0.74894800	-4.41753700
O	8.62145400	2.15790100	3.91582300
O	8.62280500	2.14655100	-3.90846700
O	9.02090400	-0.36940800	3.81405900
O	9.02221900	-0.38045300	-3.79923300
Si	9.49612700	-1.88254200	3.76523700
Si	9.49742900	-1.89344000	-3.74585600
O	8.25906500	-2.84955000	3.80762700
O	8.26037400	-2.86056400	-3.78586900
Si	7.02066400	-3.81950300	3.73361800
Si	7.02195300	-3.83029900	-3.70947500
O	7.06319800	-4.63942200	2.37331200
O	7.06401400	-4.64626800	-2.34678100
Si	5.34210900	-7.86745000	2.39819900
Si	5.34293200	-7.87435500	-2.36289500

Si	7.55974700	-5.90563500	1.55733500
Si	7.56028000	-5.91011000	-1.52696100
O	6.71610600	-7.19513600	1.95106000
O	6.71677500	-7.20074900	-1.91723400
O	7.38699900	-5.58581400	0.01464100
H	-5.31109700	-8.33783100	-3.99598300
H	-5.31248400	-8.32618900	4.02894300
H	-8.66876100	-3.96812100	5.99324900
H	-8.67444400	-3.96780000	1.91402000
H	-8.67379000	-3.97332600	-1.89487400
H	-8.66669600	-3.98547900	-5.97408400
H	-6.75996400	-3.98886300	-7.39603900
H	-6.76252100	-3.96737600	7.41586500
H	-7.59446500	0.48798700	6.09565200
H	-7.59242000	0.47026200	-6.08904100
H	-9.26661600	2.52990800	2.32382600
H	-9.26582000	2.52316100	-2.32372700
H	-7.57823100	6.31594900	3.90258900
H	-7.57688200	6.30461300	-3.91289300
H	-5.72790300	5.09107100	-4.93098700
H	-4.09253600	9.50224000	3.92912000
H	-3.86282900	8.10511800	5.88844500
H	-4.09117800	9.49081300	-3.94746400
H	-3.86079500	8.08801400	-5.90264700
H	-5.72960700	5.10536800	4.92483700
H	-4.24079000	3.11542400	-6.14244900
H	-1.84159600	3.11020700	-5.93655900
H	-4.24288200	3.13330600	6.14254100
H	-1.84361800	3.12737400	5.93749800
H	-1.40227900	5.62536500	4.85115000
H	-1.40060100	5.61128300	-4.85731100
H	1.91797800	8.22844400	4.90302500
H	0.60819100	9.89920500	3.79395300
H	0.60950600	9.88817300	-3.81182600
H	1.91967300	8.21420100	-4.91559300
H	2.12828100	4.50572100	3.85260300
H	2.12961100	4.49454200	-3.85430400
H	5.75669200	7.28590000	-3.84857000
H	6.67379600	8.52833000	-1.97472500
H	6.67311500	8.53404300	1.96290100
H	5.75536300	7.29705200	3.84002500
H	7.80984000	4.32875400	4.92830400
H	10.01716000	4.17930300	3.96681200
H	10.01853000	4.16779700	-3.96483600

H	7.81154200	4.31445500	-4.92752100
H	4.50307000	1.53688800	4.88024000
H	8.05419000	0.70847400	5.89771400
H	10.36566200	-2.15143500	4.91958100
H	10.25484400	-2.06068000	2.51883400
H	4.50475600	1.52274100	-4.87250400
H	4.58685900	-2.29185200	-5.88613000
H	10.36735700	-2.16568000	-4.89911300
H	10.25571000	-2.06795900	-2.49868100
H	4.58482000	-2.27474600	5.90496200
H	8.05622400	0.69137800	-5.88633800
H	7.04704900	-4.76858600	-4.84079500
H	8.96718300	-6.20970800	-1.82987800
H	8.96654700	-6.20435300	1.86160600
H	7.04537500	-4.75450200	4.86766600
H	5.35771900	-9.24886400	1.89585900
H	5.35836600	-9.25430900	-1.85654600
H	2.71804100	-5.27449800	4.94135500
H	2.71974500	-5.28879400	-4.91446900
H	0.69771200	-9.64083500	1.88184300
H	0.69835400	-9.64624000	-1.84300100
H	-0.68720600	-7.94535800	4.89310000
H	-0.68551800	-7.95950600	-4.85964200
H	-0.77864900	-4.29806200	5.90344900
H	-0.77661300	-4.31515500	-5.88060500
H	-4.40437200	-7.08915900	5.91039200
H	-4.40233300	-7.10626400	-5.88069900
H	-3.27196500	-1.01942300	7.45461500
H	-3.26938500	-1.04103000	-7.44213500
H	5.21019500	-7.90227900	-3.82662500
H	5.20886900	-7.89112400	3.86195800
Al	-3.47617200	-0.37741500	-1.51229200
Si	-2.88374500	-2.37167600	3.65022400
Si	-3.50485700	-0.40726200	1.50631100
Si	-2.97360600	2.44252900	2.28544500
Si	-6.57981000	-0.49232700	2.28803100
Si	-2.94684200	2.43060800	-2.27359200
Si	-2.88763700	-2.37529400	-3.61515000
Si	-6.57048800	-0.48855300	-2.28029000
O	-2.96341200	-1.76651600	2.18625800
O	-2.80596300	0.85776800	2.21565300
O	-2.89981500	-0.47907600	0.07358200
O	-5.11497400	-0.28425000	1.67185700
O	-5.15998000	-0.16132800	-1.67176000

O	-2.93192300	-1.88526600	-2.12035900
O	-2.51124700	0.90978900	-2.13025900
O	-1.43549900	-2.98065100	-3.81603200
O	-1.44723500	-3.00731000	3.76861400
O	0.78235600	-4.05965900	-3.93974500
O	0.79050300	-4.02942400	3.99334400
O	2.98491400	-2.89314200	-4.07026100
O	4.16751700	-0.71164900	-4.02538500
O	4.49193700	1.13512600	-2.34212900
O	2.96518100	3.17755100	-1.77730300
O	0.55835900	3.92759300	-2.12969600
O	-1.80102500	3.22931200	-1.47130700
O	-1.75408200	3.14485900	1.52748700
O	0.56439500	3.90929700	2.15682800
O	2.97854900	3.19693500	1.78079600
O	2.99107400	-2.87945700	4.08421900
O	4.16569700	-0.69498300	4.04454800
O	4.48631500	1.14587900	2.35259400
O	4.54591100	2.01822700	0.00106100
O	-0.45210800	4.99569300	0.02187200
Si	-0.69539100	-4.21868100	-4.46799400
Si	2.33184900	-4.33373100	-3.92792600
Si	-0.69099000	-4.20938900	4.48576100
Si	2.33757900	-4.31919600	3.95076300
Si	4.35713700	-2.19723800	4.50773100
Si	4.35553800	-2.21389800	-4.48972200
Si	4.90610000	0.67170500	-3.80882600
Si	4.90587200	0.68551800	3.81849800
Si	4.37889100	2.48184000	-1.51234600
Si	4.38468000	2.48707100	1.51131400
Si	2.08025100	4.34202500	2.44675000
Si	2.07547100	4.33168400	-2.44498300
Si	-0.79213000	4.42194100	1.47494700
Si	-0.79426200	4.45036400	-1.44511600
H	0.95761800	-3.33419800	-1.16542900
C	1.07838000	-2.35078400	-0.70338300
H	1.51273500	-2.46311700	0.29032300
H	1.76196300	-1.78197400	-1.33952000
C	-0.23453800	-1.66660900	-0.69357800
O	0.25825600	0.27233500	-1.15522500
C	0.37786900	1.08624400	0.02734700
H	0.73489000	2.07071800	-0.28923800
H	-0.60781000	1.19849300	0.48683200
C	1.36296900	0.47473500	0.99947200

H	2.32388300	0.27708600	0.51654400
H	1.52803900	1.18213300	1.81227300
H	0.99650500	-0.45381300	1.44424500
O	-1.50091600	-3.25990700	-0.17658900
H	-0.68186000	-1.22671600	0.17907100
H	-0.81046600	-1.64630200	-1.60364400
H	-2.19128700	-3.00320200	-0.81936100
H	-1.87558900	-3.00441900	0.67941800
H	-0.54627300	0.57347700	-1.62221900

TS6

O	-4.33751700	3.33895700	1.76370900
O	-3.27568800	2.49305800	3.94265700
O	-7.36638600	-1.90837700	1.86918400
O	-7.74527900	0.62678600	1.88695300
O	-6.72043600	-0.50204700	3.96058800
O	-4.22135500	-3.37655000	3.79772800
O	-3.12586900	-1.38796200	4.98945400
O	-1.22497900	-5.67669200	3.87577900
O	-4.22089800	-3.37688400	-3.79572500
O	-3.12490800	-1.38884300	-4.98761400
O	-1.22433200	-5.67720500	-3.87291400
Si	-1.41085400	-7.25203600	3.80307300
Si	-1.41021900	-7.25253700	-3.80002900
O	2.66566800	-4.96991600	2.43736000
O	-2.95810800	-7.62369500	3.89966800
O	-0.90633400	-7.79767000	2.39344800
O	2.66607400	-4.97023800	-2.43393900
O	-2.95745900	-7.62420800	-3.89683300
O	-0.90593600	-7.79798600	-2.39025000
Si	2.78238400	-6.26986000	1.53205500
Si	0.37305800	-8.22474500	1.53818600
Si	2.78264100	-6.27006200	-1.52844400
Si	0.37331400	-8.22494600	-1.53471500
O	1.59257500	-7.26352200	1.88422400
O	4.15287600	-7.00610900	1.76502000
O	2.70368000	-5.79261200	0.00172000
O	-0.05186600	-8.10952400	0.00164200
O	1.59289000	-7.26377000	-1.88067800
O	4.15317400	-7.00634000	-1.76108100
O	-4.33725600	3.33861800	-1.76252400
O	-3.27498900	2.49259200	-3.94117800
O	-7.36605300	-1.90862100	-1.86787300

O	-7.74502800	0.62655500	-1.88598700
O	-6.71982900	-0.50257000	-3.95932000
Si	-3.68686800	-0.46474300	-6.14706600
Si	-3.11636400	2.47356500	-5.51571300
Si	-6.73490300	-0.56183200	-5.52571600
O	-3.02058800	0.98031100	-6.05041000
O	-5.26820100	-0.33980100	-6.07441100
O	-7.28153000	-1.96099100	-6.03666800
Si	-3.11728200	2.47430400	5.51718500
Si	-6.73584100	-0.56106000	5.52698800
Si	-3.68791100	-0.46406000	6.14893000
O	-3.02164800	0.98110500	6.05210200
O	-7.28260300	-1.96016900	6.03803000
O	-5.26920000	-0.33905000	6.07588900
Si	-5.70799700	4.07507600	1.53064700
Si	-8.11730500	2.12022200	1.53678500
Si	-5.70772300	4.07491600	-1.52983400
Si	-8.11704900	2.12002200	-1.53610600
O	-6.89778500	3.08144600	1.88283700
O	-5.82469200	5.37505600	2.43596400
O	-5.78668600	4.55235900	0.00032700
O	-8.54223600	2.23544400	0.00025500
O	-6.89747700	3.08119400	-1.88206800
O	-5.82429200	5.37473200	-2.43532700
Si	-7.35220400	-3.45472900	1.54294900
Si	-7.35193400	-3.45492900	-1.54136600
O	-6.21144800	-4.19289900	2.36097500
O	-7.07095400	-3.68841800	0.00076400
O	-6.21104800	-4.19321900	-2.35912900
Si	-5.41842900	-4.39707100	3.72253700
O	-4.85577400	-5.87959000	3.76009300
O	-6.40132900	-4.17001400	4.94185700
Si	-7.27372800	-3.54198000	6.09983900
Si	-4.40402000	-7.23850600	4.43432100
Si	-5.41776000	-4.39762200	-3.72053900
O	-4.85514400	-5.88008300	-3.75781300
O	-6.40051100	-4.17066000	-4.94004600
Si	-7.27271300	-3.54278600	-6.09826300
Si	-4.40328100	-7.23908900	-4.43177800
O	5.67427900	-2.92987000	3.76077700
O	5.67490400	-2.93036600	-3.75712500
O	6.58140100	0.59164100	3.87602000
O	6.58204700	0.59113000	-3.87268000
O	5.57483600	3.66426000	1.88383300

O	5.57515000	3.66400900	-1.88106700
O	2.54965600	5.87329900	1.80383400
O	2.54995600	5.87306000	-1.80186500
O	-1.42601500	5.75626700	2.36071400
O	-1.42562500	5.75595800	-2.35938600
Si	-6.18290700	6.00958800	3.85069800
Si	-6.18226400	6.00908300	-3.85020200
Si	-1.47179900	6.57412300	3.72215400
Si	-1.47118000	6.57363100	-3.72094800
Si	6.78152100	4.63736200	1.53167000
Si	6.78177500	4.63715900	-1.52883000
Si	2.78849400	7.40461200	1.52775300
Si	2.78874700	7.40440800	-1.52594500
O	-5.41230400	7.40351400	3.97607000
O	-5.41164200	7.40299000	-3.97562900
O	-0.23740300	7.54906800	3.79739200
O	-2.81609000	7.41509900	3.75938600
O	-0.23677000	7.54856700	-3.79610800
O	-2.81546200	7.41460000	-3.75851200
Si	-4.06089000	8.12331800	4.43333700
Si	-4.06014800	8.12273400	-4.43276400
O	4.29820500	7.77927800	1.83565300
O	1.81772100	8.33362500	2.40651000
O	2.47524900	7.74409100	0.00080700
O	4.29851400	7.77903700	-1.83364500
O	1.81812300	8.33330800	-2.40498800
Si	0.99559800	8.52130200	3.75623000
Si	0.99622300	8.52080500	-3.75486700
O	6.32667500	6.12516900	1.76433300
O	8.03361300	4.26949300	2.43719900
O	7.23445400	4.46683800	0.00141800
O	6.32696800	6.12493300	-1.76176600
O	8.03401900	4.26917200	-2.43410200
Si	5.75927500	7.46527900	2.38169700
Si	5.75966900	7.46496300	-2.37940300
Si	8.58591500	3.79463000	3.85207500
Si	8.58656100	3.79412400	-3.84882400
Si	8.04800700	0.81375500	4.42492600
Si	8.04874400	0.81316900	-4.42137300
O	8.59472000	2.21284700	3.91388000
O	8.59537200	2.21233100	-3.91041800
O	9.00457600	-0.31265000	3.80858100
O	9.00520900	-0.31315300	-3.80471900
Si	9.48602700	-1.82374400	3.75762200

Si	9.48665600	-1.82424000	-3.75347900
O	8.25296500	-2.79590100	3.79878500
O	8.25359500	-2.79640100	-3.79471900
Si	7.01856500	-3.77084600	3.72354400
Si	7.01918800	-3.77133600	-3.71955700
O	7.06435000	-4.58870000	2.36210000
O	7.06474200	-4.58901100	-2.35799800
Si	5.35658300	-7.82382600	2.38267200
Si	5.35698000	-7.82413800	-2.37842600
Si	7.56603600	-5.85172400	1.54432700
Si	7.56629200	-5.85192800	-1.53997200
O	6.72775500	-7.14523500	1.93634200
O	6.72807800	-7.14549300	-1.93195700
O	7.39182300	-5.53048300	0.00209200
H	-5.29520400	-8.32926700	-4.01119800
H	-5.29587300	-8.32873600	4.01373600
H	-8.66989900	-3.98726400	5.98437400
H	-8.67597200	-3.98132200	1.90515100
H	-8.67565800	-3.98157400	-1.90374800
H	-8.66890200	-3.98805200	-5.98297100
H	-6.76230900	-3.98160800	-7.40510000
H	-6.76354300	-3.98063000	7.40681900
H	-7.61397000	0.47308800	6.09285000
H	-7.61301500	0.47223500	-6.09185700
H	-9.29488300	2.51331800	2.32400300
H	-9.29450200	2.51300500	-2.32355500
H	-7.62196800	6.30409800	3.90785500
H	-7.62131700	6.30358400	-3.90763600
H	-5.76745000	5.09908300	-4.92757500
H	-4.14943400	9.50469100	3.93848400
H	-3.91378300	8.10581800	5.89585200
H	-4.14877900	9.50417000	-3.93810800
H	-3.91280300	8.10503900	-5.89525200
H	-5.76827100	5.09973400	4.92826000
H	-4.27232100	3.13126200	-6.14190300
H	-1.87310600	3.13564900	-5.93623600
H	-4.27331500	3.13213300	6.14309900
H	-1.87406600	3.13637600	5.93783300
H	-1.44313100	5.63766500	4.85490500
H	-1.44232100	5.63702500	-4.85356700
H	1.86637300	8.25433400	4.91008500
H	0.54960500	9.92121700	3.80344500
H	0.55023900	9.92071600	-3.80234300
H	1.86719100	8.25368600	-4.90854300

H	2.09191800	4.53396700	3.85449300
H	2.09256100	4.53346000	-3.85242300
H	5.70810500	7.33973500	-3.84315000
H	6.62025700	8.58334000	-1.96766900
H	6.61993000	8.58360000	1.96996100
H	5.70746400	7.34024100	3.84545300
H	7.77426100	4.37893300	4.92943700
H	9.98208700	4.23991300	3.96754200
H	9.98274800	4.23939000	-3.96411500
H	7.77508300	4.37828100	-4.92639800
H	4.47902300	1.57352900	4.87780500
H	8.03362500	0.75835300	5.89381300
H	10.35677200	-2.09064700	4.91151500
H	10.24535200	-1.99702700	2.51090600
H	4.47983700	1.57288600	-4.87494800
H	4.57756600	-2.23992900	-5.89386200
H	10.35758900	-2.09129600	-4.90719000
H	10.24577100	-1.99735900	-2.50661400
H	4.57658200	-2.23915000	5.89724300
H	8.03460500	0.75757400	-5.89025100
H	7.04804400	-4.70794600	-4.85217700
H	8.97439000	-6.14530600	-1.84343000
H	8.97408400	-6.14506200	1.84805800
H	7.04723800	-4.70730400	4.85629400
H	5.37783800	-9.20446800	1.87842000
H	5.37815100	-9.20471700	-1.87398900
H	2.72209100	-5.24523500	4.92965100
H	2.72291500	-5.24588400	-4.92618300
H	0.71948600	-9.61562500	1.86427900
H	0.71979500	-9.61587200	-1.86056900
H	-0.67212100	-7.93004000	4.87800400
H	-0.67130700	-7.93068400	-4.87474800
H	-0.77850200	-4.28455400	5.89341000
H	-0.77752000	-4.28533100	-5.89065700
H	-4.39268800	-7.09058000	5.89681500
H	-4.39170400	-7.09135700	-5.89428900
H	-3.28516300	-1.01837000	7.44933700
H	-3.28391600	-1.01935000	-7.44742900
H	5.22422000	-7.85058500	-3.84218200
H	5.22358100	-7.85007600	3.84641000
Al	-3.48805400	-0.38383600	-1.51566100
Si	-2.89758100	-2.36074200	3.64442500
Si	-3.53169800	-0.38416300	1.49884500
Si	-2.99941200	2.45055600	2.28310300

Si	-6.59633600	-0.49568300	2.28168700
Si	-2.98086300	2.43847100	-2.27514500
Si	-2.89191300	-2.35200200	-3.61365200
Si	-6.58615700	-0.49054300	-2.28202600
O	-3.03492300	-1.76145800	2.18510300
O	-2.83116100	0.86375800	2.22792000
O	-2.91216600	-0.47713600	0.07512200
O	-5.14196500	-0.25765300	1.65512400
O	-5.18434100	-0.21114900	-1.64073400
O	-2.91882500	-1.89327700	-2.10656900
O	-2.60925500	0.91612700	-2.16522400
O	-1.44114400	-2.95385900	-3.83803200
O	-1.45214100	-2.98782400	3.74595600
O	0.78109100	-4.03458000	-3.96659500
O	0.78487100	-3.99799100	3.97039800
O	2.97899400	-2.84988400	-4.06949400
O	4.15254600	-0.66466900	-4.02234400
O	4.47037300	1.18283600	-2.34137300
O	2.92900300	3.21547400	-1.78843000
O	0.52025000	3.95729700	-2.12838900
O	-1.83456000	3.23576500	-1.46744500
O	-1.78015100	3.14994200	1.52404100
O	0.52372300	3.94720200	2.16149700
O	2.92886600	3.20784700	1.79110700
O	2.98734500	-2.85088500	4.06967500
O	4.15139800	-0.66263900	4.01857400
O	4.47283100	1.18460000	2.34313600
O	4.50766100	2.07365800	-0.00174500
O	-0.49915000	5.01737200	0.02020000
Si	-0.70197900	-4.18896400	-4.48331900
Si	2.32967600	-4.29416700	-3.93999000
Si	-0.69731600	-4.18939700	4.47144400
Si	2.33257400	-4.29261700	3.94191500
Si	4.34935600	-2.16041700	4.49935300
Si	4.34413300	-2.16500000	-4.49577500
Si	4.88502400	0.72269200	-3.81157600
Si	4.88689100	0.72734200	3.81282400
Si	4.34544500	2.53443500	-1.51807400
Si	4.34892700	2.53383700	1.51180800
Si	2.03751900	4.37079800	2.44829500
Si	2.03033400	4.37233500	-2.44491400
Si	-0.83584200	4.43963300	1.47155600
Si	-0.84208200	4.46354000	-1.44518100
H	-0.06979100	-4.03561800	-0.98186700

C	0.46850000	-3.11484100	-0.75059900
H	1.39921800	-3.40376400	-0.25402200
H	0.68871400	-2.60273000	-1.68604500
C	-0.38080100	-2.28202800	0.14394900
O	0.89334300	-0.82047500	0.52033700
C	0.67499300	0.44803000	-0.12447900
H	1.39169000	1.16249800	0.29549000
H	-0.33121400	0.76593300	0.16268700
C	0.79025800	0.36349500	-1.63503300
H	0.02765600	-0.30090200	-2.04971100
H	0.61372100	1.35635200	-2.05505000
H	1.77874200	0.01685700	-1.94891100
C	2.14883900	-1.05896800	1.16492500
H	2.31114600	-0.26425500	1.89990500
H	2.00491300	-1.98997400	1.72060500
C	3.32514400	-1.16581700	0.21362500
H	3.52110700	-0.22481700	-0.30077400
H	4.22278400	-1.41136600	0.78950200
H	3.16670000	-1.94654200	-0.53510400
O	-2.10463000	-3.43144000	0.00732200
H	-0.41703700	-2.47813000	1.20689600
H	-1.02869200	-1.50678700	-0.22993500
H	-2.48794800	-3.02955500	-0.80270400
H	-2.63410100	-3.05565000	0.72662000

TS7

O	4.33381700	3.31126300	-1.75997100
O	3.27245300	2.46268900	-3.93810900
O	7.36585800	-1.93435600	-1.86059100
O	7.74318900	0.60101600	-1.88072900
O	6.71902900	-0.53037200	-3.95330200
O	4.22164800	-3.40622000	-3.78767900
O	3.12497000	-1.41942400	-4.98127100
O	1.22669800	-5.70827500	-3.86353300
O	4.22131800	-3.39942500	3.80572100
O	3.12419300	-1.41095300	4.99582800
O	1.22618800	-5.70153400	3.88514600
Si	1.41355300	-7.28343100	-3.78936800
Si	1.41304600	-7.27681500	3.81374600
O	-2.66433900	-5.00251600	-2.42570700
O	2.96102300	-7.65424200	-3.88563800
O	0.90938300	-7.82805500	-2.37921700
O	-2.66466200	-4.99827600	2.44558900

O	2.96050900	-7.64745400	3.91086400
O	0.90906900	-7.82389100	2.40447800
Si	-2.78024900	-6.30168300	-1.51918900
Si	-0.36973400	-8.25510700	-1.52353200
Si	-2.78045500	-6.29901900	1.54131000
Si	-0.36993800	-8.25243000	1.54936900
O	-1.58984600	-7.29495300	-1.87045000
O	-4.15030000	-7.03898300	-1.75143900
O	-2.70181100	-5.82295400	0.01069700
O	0.05514600	-8.13818800	0.01289600
O	-1.59009300	-7.29167300	1.89445200
O	-4.15053400	-7.03591300	1.77466100
O	4.33360800	3.31422700	1.76625800
O	3.27185100	2.46975200	3.94572700
O	7.36558300	-1.93108700	1.87648000
O	7.74302800	0.60433100	1.89220300
O	6.71856100	-0.52347300	3.96661800
Si	3.68560900	-0.48544600	6.15437500
Si	3.11330500	2.45182100	5.52028000
Si	6.73368100	-0.58126400	5.53306200
O	3.01843200	0.95911300	6.05638300
O	5.26685700	-0.35959100	6.08156800
O	7.28116800	-1.97960800	6.04531400
Si	3.11401800	2.44231800	-5.51261200
Si	6.73443100	-0.59083300	-5.51963700
Si	3.68643100	-0.49627400	-6.14161400
O	3.01928700	0.94857400	-6.04613100
O	7.28204300	-1.99008800	-6.02937900
O	5.26764400	-0.37023800	-6.06871700
Si	5.70383500	4.04841000	-1.52761600
Si	8.11433200	2.09502200	-1.53197500
Si	5.70361700	4.05111200	1.53285300
Si	8.11412600	2.09769900	1.54091700
O	6.89422300	3.05517700	-1.87889600
O	5.81972300	5.34761600	-2.43415600
O	5.78226000	4.52717500	0.00224800
O	8.53922100	2.21193900	0.00444200
O	6.89397800	3.05845100	1.88599700
O	5.81940900	5.35185100	2.43713300
Si	7.35262700	-3.48039700	-1.53289400
Si	7.35240900	-3.47770800	1.55140400
O	6.21230600	-4.22002400	-2.35021700
O	7.07154100	-3.71281300	0.00950700
O	6.21199500	-4.21592000	2.36989500

Si	5.41938500	-4.42595600	-3.71157400
O	4.85763000	-5.90885300	-3.74773000
O	6.40212500	-4.19944600	-4.93112200
Si	7.27412100	-3.57196500	-6.08970600
Si	4.40669200	-7.26867500	-4.42067600
Si	5.41884400	-4.41954100	3.73150300
O	4.85713200	-5.90230700	3.77017000
O	6.40147200	-4.19084400	4.95078700
Si	7.27331900	-3.56134700	6.10839100
Si	4.40610900	-7.26095400	4.44542000
O	-5.67418400	-2.96551700	-3.75096000
O	-5.67471500	-2.95900400	3.76692100
O	-6.58346200	0.55503800	-3.86964000
O	-6.58400300	0.56215400	3.87910000
O	-5.57922300	3.62984900	-1.88041100
O	-5.57860800	3.63404900	1.88458600
O	-2.55447600	5.84112900	-1.80194100
O	-2.55506300	5.84451200	1.80332000
O	1.42085300	5.72611500	-2.35924700
O	1.42050100	5.73031800	2.36089600
Si	6.17752800	5.98104100	-3.84949200
Si	6.17701900	5.98774700	3.85140500
Si	1.46606200	6.54286900	-3.72136800
Si	1.46557900	6.54930100	3.72171000
Si	-6.78569800	4.60352500	-1.52901300
Si	-6.78642100	4.60554400	1.53162300
Si	-2.79476200	7.37280900	-1.52792400
Si	-2.79495500	7.37542900	1.52603300
O	5.40607400	7.37437900	-3.97615500
O	5.40555100	7.38130100	3.97554000
O	0.23108600	7.51694900	-3.79752300
O	2.80985600	7.38458900	-3.75943500
O	0.23059100	7.52356600	3.79597500
O	2.80936400	7.39112500	3.75846200
Si	4.05421300	8.09293100	-4.43407200
Si	4.05362800	8.10065200	4.43202500
O	-4.30461400	7.74624700	-1.83595400
O	-1.82451300	8.30156100	-2.40736500
O	-2.48168200	7.71386600	-0.00105000
O	-4.30486800	7.74943600	1.83338600
O	-1.82479800	8.30574900	2.40414900
Si	-1.00250200	8.48847300	-3.75724800
Si	-1.00299700	8.49501000	3.75384800
O	-6.33207500	6.09096600	-1.76297800

O	-8.03791700	4.23359900	-2.43408600
O	-7.23882000	4.43373900	0.00145700
O	-6.33232800	6.09402900	1.76311900
O	-8.03824000	4.23783300	2.43716200
Si	-5.76551200	7.43084100	-2.38164000
Si	-5.76582300	7.43498400	2.37945900
Si	-8.58994500	3.75709800	-3.84852900
Si	-8.59045600	3.76380100	3.85236700
Si	-8.05025100	0.77613100	-4.41850300
Si	-8.05080300	0.78365200	4.42775200
O	-8.59778200	2.17525800	-3.90886600
O	-8.59830500	2.18206000	3.91544100
O	-9.00610800	-0.35038600	-3.80116900
O	-9.00660400	-0.34377100	3.81212200
Si	-9.48663700	-1.86173300	-3.74879600
Si	-9.48713400	-1.85519600	3.76230000
O	-8.25298300	-2.83317700	-3.78907100
O	-8.25348000	-2.82656600	3.80443000
Si	-7.01799400	-3.80729200	-3.71294100
Si	-7.01848000	-3.80082400	3.73016100
O	-7.06325000	-4.62391200	-2.35073400
O	-7.06356500	-4.61979600	2.36936300
Si	-5.35351900	-7.85801000	-2.36830500
Si	-5.35383200	-7.85386500	2.39279100
Si	-7.56415500	-5.88647000	-1.53176500
Si	-7.56435800	-5.88378500	1.55253200
O	-6.72509500	-7.17983800	-1.92258400
O	-6.72535000	-7.17647300	1.94571300
O	-7.39011100	-5.56367900	0.01016500
H	5.29868600	-8.35098500	4.02584500
H	5.29921400	-8.35796800	-3.99908500
H	8.67056400	-4.01629200	-5.97385000
H	8.67670500	-4.00652500	-1.89463400
H	8.67645700	-4.00320900	1.91426200
H	8.66977600	-4.00587200	5.99349100
H	6.76320600	-3.99925600	7.41564800
H	6.76418100	-4.01214800	-7.39626500
H	7.61191000	0.44332200	-6.08648400
H	7.61118100	0.45386900	6.09821800
H	9.29165500	2.48809600	-2.31958100
H	9.29135500	2.49213600	2.32797700
H	7.61640700	6.27637200	-3.90695100
H	7.61589200	6.28317700	3.90853700
H	5.76277700	5.07850400	4.92963800

H	4.14192600	9.47482100	-3.94051500
H	3.90709100	8.07397300	-5.89656800
H	4.14140900	9.48167800	3.93607600
H	3.90631700	8.08423600	5.89453200
H	5.76342700	5.06992500	-4.92619400
H	4.26887200	3.11090200	6.14583500
H	1.86964800	3.11364900	5.94020700
H	4.26964600	3.10027800	-6.13916300
H	1.87039900	3.10325500	-5.93385800
H	1.43797300	5.60529300	-4.85326400
H	1.43732300	5.61374500	4.85520200
H	-1.87313500	8.21989600	-4.91083800
H	-0.55736200	9.88861500	-3.80578000
H	-0.55786300	9.89523500	3.80000400
H	-1.87378200	8.22844300	4.90778800
H	-2.09666600	4.50055800	-3.85151900
H	-2.09691700	4.50708100	3.85519300
H	-5.71415800	7.31115700	3.84332100
H	-6.62709900	8.55245000	1.96669300
H	-6.62684100	8.54902100	-1.97093700
H	-5.71364900	7.30446300	-3.84528000
H	-7.77866600	4.34088400	-4.92645300
H	-9.98639000	4.20142100	-3.96438700
H	-9.98691000	4.20832600	3.96726600
H	-7.77931400	4.34946100	4.92937900
H	-4.48174600	1.53758900	-4.87236700
H	-8.03584100	0.71924300	-5.88743300
H	-10.35723800	-2.13024700	-4.90242500
H	-10.24583400	-2.03431100	-2.50190700
H	-4.48238900	1.54600600	4.88049100
H	-4.57775500	-2.26590400	5.90299200
H	-10.35788300	-2.12170100	4.91627600
H	-10.24616400	-2.02994300	2.51561200
H	-4.57696200	-2.27612000	-5.88809100
H	-8.03661600	0.72950300	5.89662800
H	-7.04674600	-4.73638800	4.86365200
H	-8.97227200	-6.17773500	1.85629000
H	-8.97202900	-6.18094900	-1.83519800
H	-7.04611400	-4.74483100	-4.84481800
H	-5.37392500	-9.23819500	-1.86275900
H	-5.37417200	-9.23492900	1.88964900
H	-2.72063800	-5.28019000	-4.91773900
H	-2.72129100	-5.27162900	4.93808900
H	-0.71532200	-9.64650200	-1.84831700

H	-0.71556600	-9.64326100	1.87653100
H	0.67520400	-7.96289400	-4.86364300
H	0.67456200	-7.95440500	4.88910500
H	0.77935400	-4.31830000	-5.88246700
H	0.77857900	-4.30805100	5.90159800
H	4.39524400	-7.12212500	-5.88330800
H	4.39446600	-7.11186000	5.90779200
H	3.28400600	-1.05205000	-7.44149600
H	3.28302300	-1.03908300	7.45526600
H	-5.22103100	-7.87885900	3.85657000
H	-5.22052600	-7.88555100	-3.83201900
Al	3.53156200	-0.43056800	1.53626700
Si	2.88419300	-2.40904200	-3.65832500
Si	3.55659800	-0.43177200	-1.49088800
Si	2.98909200	2.44239000	-2.26997200
Si	6.60189200	-0.52358100	-2.27460500
Si	2.97659700	2.42319300	2.27124800
Si	2.88322200	-2.38613200	3.63621800
Si	6.59198700	-0.51295400	2.28917000
O	3.00994800	-1.71017700	-2.26311600
O	2.81643100	0.87353400	-2.06450100
O	2.93971100	-0.69714100	-0.06065200
O	5.15488400	-0.27115000	-1.62534800
O	5.21227700	-0.19827800	1.61192900
O	2.92171300	-1.87290700	2.16692900
O	2.61437000	0.91177100	2.04518100
O	1.43118600	-2.97394100	3.86770000
O	1.43596300	-3.02375000	-3.74695300
O	-0.78145800	-4.06656900	3.96980600
O	-0.79367300	-4.03508000	-3.96876700
O	-2.97423100	-2.87490200	4.08853900
O	-4.15292700	-0.69282700	4.03763800
O	-4.47912100	1.14999300	2.34877400
O	-2.94526200	3.18559500	1.79259200
O	-0.53588300	3.92050100	2.12575100
O	1.82997000	3.23350800	1.48114700
O	1.76592200	3.14708000	-1.52859900
O	-0.53993700	3.91233000	-2.15237300
O	-2.94213000	3.17664700	-1.78274800
O	-2.98542700	-2.87687100	-4.05820500
O	-4.15514700	-0.69427600	-4.02866900
O	-4.46864300	1.14091400	-2.33484600
O	-4.52766700	2.03277400	0.00709100
O	0.47471700	5.00276000	-0.01412100

Si	0.69702700	-4.21628300	4.49497600
Si	-2.32847600	-4.32112700	3.94807200
Si	0.68634100	-4.22930200	-4.46479500
Si	-2.34078900	-4.32420800	-3.92860400
Si	-4.35151600	-2.19756900	-4.49172800
Si	-4.34295800	-2.19558800	4.50637700
Si	-4.88757200	0.69075600	3.82113700
Si	-4.88940100	0.69253800	-3.80604700
Si	-4.36012000	2.49872200	1.52190400
Si	-4.35880400	2.49313200	-1.50584900
Si	-2.05255800	4.33667400	-2.44504200
Si	-2.04279200	4.34187800	2.44675800
Si	0.81291700	4.42534700	-1.46430800
Si	0.82211200	4.44499000	1.44986100
C	0.93527400	-2.54762100	-0.14906900
H	0.81513500	-2.87329100	-1.18161400
H	0.12605000	-2.93402400	0.47560800
C	0.95250200	-1.07084500	-0.02159400
H	0.89581800	-0.39637900	-0.85830700
H	0.94804100	-0.62473400	0.95915800
O	-1.13036200	-0.83869300	0.01309800
C	-1.53035400	0.34820700	0.70841700
H	-2.61253100	0.44544500	0.57718400
H	-1.04956000	1.22875600	0.26239300
C	-1.18850900	0.22400000	2.18043600
H	-0.11333900	0.27446600	2.36832300
H	-1.64286500	1.06285900	2.71075500
H	-1.59003000	-0.70466800	2.59292200
C	-1.74892200	-1.02629500	-1.26870200
H	-1.55603000	-2.07042200	-1.52876200
H	1.86978500	-2.93099900	0.26480500
H	-2.83115400	-0.90229400	-1.14137300
C	-1.23747000	-0.09685700	-2.35875400
H	-1.80183600	-0.30264000	-3.27066300
H	-1.37867400	0.95614400	-2.10130800
H	-0.18133100	-0.26727300	-2.58388500

TS8

O	-4.30863300	3.30940700	1.76092400
O	-3.24818400	2.46433800	3.94086300
O	-7.34245700	-1.93500500	1.86996300
O	-7.71897300	0.60052200	1.88549200
O	-6.69575600	-0.52752600	3.96036800

O	-4.19932900	-3.40448500	3.80062600
O	-3.10232300	-1.41594000	4.99097800
O	-1.20514200	-5.70740700	3.88146400
O	-4.19679500	-3.41117700	-3.79305200
O	-3.09854700	-1.42518500	-4.98616900
O	-1.20242300	-5.71447700	-3.86731100
Si	-1.39247800	-7.28263100	3.81002100
Si	-1.38967000	-7.28954500	-3.79304000
O	2.68656500	-5.00549000	2.44353600
O	-2.94011000	-7.65275500	3.90649000
O	-0.88807900	-7.82992800	2.40099600
O	2.68835900	-5.00994900	-2.42775800
O	-2.93727200	-7.65984000	-3.89000600
O	-0.88634400	-7.83427400	-2.38269800
Si	2.80231500	-6.30630700	1.53935400
Si	0.39115300	-8.25892400	1.54645400
Si	2.80342600	-6.30908800	-1.52113900
Si	0.39227100	-8.26171600	-1.52644600
O	1.61147700	-7.29855700	1.89202500
O	4.17205200	-7.04364600	1.77332400
O	2.72448500	-5.83027800	0.00859500
O	-0.03323300	-8.14459900	0.00969500
O	1.61284600	-7.30197700	-1.87287200
O	4.17333700	-7.04684900	-1.75277300
O	-4.30748100	3.30615100	-1.76531800
O	-3.24536200	2.45715500	-3.94293100
O	-7.34104200	-1.93838300	-1.86696000
O	-7.71759900	0.59709000	-1.88733300
O	-6.69287800	-0.53476900	-3.95943800
Si	-3.65927900	-0.50158600	-6.14649000
Si	-3.08622500	2.43671400	-5.51743500
Si	-6.70763200	-0.59528400	-5.52590000
O	-2.99176200	0.94293000	-6.05082600
O	-5.24056500	-0.37512700	-6.07438600
O	-7.25542400	-1.99437100	-6.03582600
Si	-3.09025000	2.44674800	5.51545800
Si	-6.71166600	-0.58520600	5.52679900
Si	-3.66381400	-0.49055400	6.14952200
O	-2.99616800	0.95391000	6.05165900
O	-7.25988200	-1.98337100	6.03886600
O	-5.24496900	-0.36411700	6.07592500
Si	-5.67834200	4.04659300	1.52685000
Si	-8.08948300	2.09400800	1.53395000
Si	-5.67721500	4.04384700	-1.53363800

Si	-8.08842200	2.09122100	-1.53900900
O	-6.86916400	3.05437600	1.87953200
O	-5.79406900	5.34744400	2.43102800
O	-5.75615300	4.52266100	-0.00390500
O	-8.51389300	2.20832900	-0.00280300
O	-6.86779600	3.05095200	-1.88536600
O	-5.79230500	5.34301100	-2.44025900
Si	-7.32961700	-3.48165400	1.54502800
Si	-7.32852100	-3.48446700	-1.53935200
O	-6.18979700	-4.22020200	2.36400000
O	-7.04817100	-3.71690600	0.00312200
O	-6.18803200	-4.22454000	-2.35607500
Si	-5.39735000	-4.42397100	3.72595500
O	-4.83609600	-5.90698800	3.76490700
O	-6.38037000	-4.19497400	4.94481100
Si	-7.25251100	-3.56513800	6.10200900
Si	-4.38580800	-7.26576000	4.44041400
Si	-5.39459200	-4.43079700	-3.71710900
O	-4.83335900	-5.91381800	-3.75302200
O	-6.37681300	-4.20390900	-4.93707000
Si	-7.24807400	-3.57622600	-6.09608600
Si	-4.38258400	-7.27381600	-4.42568000
O	5.69672100	-2.96715900	3.76607500
O	5.69946700	-2.97400000	-3.75182200
O	6.60711900	0.55359300	3.87860700
O	6.60993800	0.54655000	-3.87009100
O	5.60400100	3.62547700	1.88354700
O	5.60537000	3.62205300	-1.88135100
O	2.58091500	5.83729000	1.80083300
O	2.58222000	5.83400900	-1.80485600
O	-1.39502100	5.72446000	2.35670100
O	-1.39332900	5.72013800	-2.36342500
Si	-6.15208600	5.98350300	3.84512800
Si	-6.14928500	5.97650600	-3.85576700
Si	-1.44040900	6.54350900	3.71743100
Si	-1.43769000	6.53675600	-3.72565300
Si	6.81169800	4.59714800	1.53090500
Si	6.81281000	4.59436600	-1.52959300
Si	2.82127500	7.36814300	1.52353600
Si	2.82238800	7.36536600	-1.53016800
O	-5.38020900	7.37681100	3.96954300
O	-5.37731800	7.36958300	-3.98215200
O	-0.20512400	7.51735400	3.79219800
O	-2.78392300	7.38577500	3.75357900

O	-0.20236100	7.51045200	-3.80129800
O	-2.78118700	7.37894300	-3.76431300
Si	-4.02824600	8.09572900	4.42658400
Si	-4.02501900	8.08767200	-4.43951400
O	4.33124900	7.74165100	1.83154100
O	1.85112800	8.29880800	2.40123600
O	2.50877600	7.70663000	-0.00378700
O	4.33258800	7.73831500	-1.83775400
O	1.85288000	8.29443500	-2.41026200
Si	1.02880300	8.48839400	3.75056300
Si	1.03153300	8.48156700	-3.76053100
O	6.35818500	6.08557700	1.76218600
O	8.06318900	4.22886600	2.43709500
O	7.26489800	4.42491000	0.00092500
O	6.35946700	6.08237000	-1.76391300
O	8.06496000	4.22444000	-2.43420400
Si	5.79186800	7.42673900	2.37826100
Si	5.79359800	7.42241100	-2.38283800
Si	8.61464800	3.75467900	3.85252600
Si	8.61745200	3.74768200	-3.84837100
Si	8.07378000	0.77479200	4.42773500
Si	8.07699700	0.76675200	-4.41855700
O	8.62195000	2.17294000	3.91566600
O	8.62479600	2.16582900	-3.90863100
O	9.02946200	-0.35303000	3.81261000
O	9.03223100	-0.35994900	-3.80068600
Si	9.50950800	-1.86461800	3.76305800
Si	9.51224400	-1.87144400	-3.74803800
O	8.27552200	-2.83557900	3.80469500
O	8.27828100	-2.84247900	-3.78880400
Si	7.04022900	-3.80942700	3.72992900
Si	7.04294100	-3.81618600	-3.71316800
O	7.08562600	-4.62847100	2.36918800
O	7.08734300	-4.63276200	-2.35091000
Si	5.37481600	-7.86197300	2.39200500
Si	5.37654800	-7.86629900	-2.36909000
Si	7.58635400	-5.89265400	1.55261900
Si	7.58747600	-5.89545800	-1.53168000
O	6.74674900	-7.18504900	1.94548800
O	6.74815700	-7.18856700	-1.92280900
O	7.41287700	-5.57255100	0.01006300
H	-5.27564700	-8.36280000	-4.00443300
H	-5.27856700	-8.35550600	4.02049600
H	-8.64906500	-4.00920800	5.98652700

H	-8.65399200	-4.00669500	1.90729400
H	-8.65259700	-4.01016300	-1.90162200
H	-8.64471300	-4.02008200	-5.98081100
H	-6.73771700	-4.01662800	-7.40240800
H	-6.74310400	-4.00316500	7.40950300
H	-7.58898000	0.45023900	6.09154100
H	-7.58461200	0.43911400	-6.09315700
H	-9.26692200	2.48887000	2.32050000
H	-9.26521800	2.48465000	-2.32706800
H	-7.59088500	6.27941300	3.90163300
H	-7.58804400	6.27231100	-3.91385400
H	-5.73502400	5.06521100	-4.93225600
H	-4.11535600	9.47676700	3.93054200
H	-3.88156700	8.07932200	5.88915400
H	-4.11249100	9.46960800	-3.94604700
H	-3.87728100	8.06860600	-5.90194500
H	-5.73860800	5.07416900	4.92357500
H	-4.24139600	3.09497300	-6.14450500
H	-1.84224000	3.09726500	-5.93816800
H	-4.24584000	3.10618900	6.14049100
H	-1.84653400	3.10801200	5.93589600
H	-1.41294200	5.60797600	4.85098100
H	-1.40941100	5.59915200	-4.85748400
H	1.89900400	8.22158100	4.90488900
H	0.58411100	9.88876700	3.79647400
H	0.58687800	9.88185400	-3.80931000
H	1.90257400	8.21265800	-4.91373500
H	2.12135000	4.50011800	3.85249400
H	2.12415800	4.49311700	-3.85441900
H	5.74232700	7.29599700	-3.84649300
H	6.65512300	8.54032600	-1.97180400
H	6.65369000	8.54390500	1.96582600
H	5.73952900	7.30298400	3.84210700
H	7.80324200	4.34064900	4.92916800
H	10.01120500	4.19874600	3.96800900
H	10.01409000	4.19153800	-3.96364300
H	7.80682500	4.33169200	-4.92666300
H	4.50538600	1.53830000	4.87897400
H	8.05893400	0.72064300	5.89666600
H	10.37967800	-2.13136700	4.91742200
H	10.26902100	-2.03966400	2.51670500
H	4.50893200	1.52943500	-4.87377800
H	4.60337800	-2.28433100	-5.88945100
H	10.38325000	-2.14028900	-4.90127900

H	10.27084500	-2.04422500	-2.50081400
H	4.59907800	-2.27360800	5.90165300
H	8.06321900	0.70993200	-5.88739400
H	7.07123300	-4.75378000	-4.84499200
H	8.99538300	-6.19041600	-1.83449300
H	8.99404200	-6.18706200	1.85699400
H	7.06770300	-4.74495600	4.86347600
H	5.39491600	-9.24306000	1.88892100
H	5.39628100	-9.24647200	-1.86348500
H	2.74203800	-5.27876900	4.93607300
H	2.74564600	-5.28776500	-4.91973900
H	0.73618500	-9.64985300	1.87381700
H	0.73753600	-9.65324000	-1.85103000
H	-0.65468200	-7.96042300	4.88572900
H	-0.65113000	-7.96928800	-4.86701600
H	-0.75792300	-4.31400000	5.89805200
H	-0.75355600	-4.32481200	-5.88609700
H	-4.37474700	-7.11661200	5.90278500
H	-4.37045900	-7.12732800	-5.88831200
H	-3.26196000	-1.04413900	7.45050200
H	-3.25647600	-1.05764400	-7.44625000
H	5.24417400	-7.89385400	-3.83286200
H	5.24137800	-7.88686600	3.85572600
Al	-3.47437500	-0.41535100	-1.50761300
Si	-2.86077200	-2.40703000	3.66739200
Si	-3.51517800	-0.44335600	1.48753200
Si	-2.96410300	2.43922200	2.27464300
Si	-6.57139500	-0.52444500	2.28011200
Si	-2.94316600	2.42647800	-2.27132900
Si	-2.85880900	-2.40299000	-3.63862700
Si	-6.56770500	-0.52385000	-2.28407800
O	-2.96779800	-1.73286600	2.25335100
O	-2.81153500	0.86660200	2.09102100
O	-2.87238200	-0.67945600	0.06763400
O	-5.12258900	-0.30213600	1.62128200
O	-5.15748000	-0.26447700	-1.64866200
O	-2.80580600	-1.87631500	-2.14584100
O	-2.58266700	0.90821700	-2.07612000
O	-1.41374200	-3.00596200	-3.85453400
O	-1.41440900	-3.02489400	3.75945700
O	0.80905300	-4.06639200	-3.93484100
O	0.81583200	-4.04828400	3.98532200
O	3.00286700	-2.88893400	-4.07943400
O	4.17686600	-0.70258400	-4.03527200

O	4.50290900	1.13493000	-2.34021900
O	2.96985400	3.17200500	-1.79439600
O	0.55917300	3.91946000	-2.13179100
O	-1.80490800	3.23070900	-1.47058500
O	-1.74183500	3.13968100	1.52840100
O	0.56262000	3.91493200	2.16426500
O	2.96993700	3.17652800	1.78802800
O	3.00824900	-2.88084600	4.08461200
O	4.17461700	-0.69161200	4.04799300
O	4.50021400	1.14092900	2.34528200
O	4.55032900	2.02793000	-0.00001900
O	-0.44797300	4.99691200	0.01826900
Si	-0.65777400	-4.24037100	-4.48242000
Si	2.36157000	-4.33245700	-3.93114600
Si	-0.66310700	-4.22795100	4.48139100
Si	2.36578900	-4.32351900	3.94647000
Si	4.37342300	-2.19326100	4.50529600
Si	4.37330400	-2.20457100	-4.49370900
Si	4.91372100	0.68099600	-3.80968800
Si	4.91153600	0.69027500	3.81550300
Si	4.38267100	2.48471400	-1.51386800
Si	4.38369300	2.48686000	1.51199900
Si	2.08013300	4.33285100	2.44725700
Si	2.07235300	4.32844800	-2.44620500
Si	-0.78608900	4.41914900	1.46887900
Si	-0.79109000	4.44148200	-1.44554100
C	-0.62756200	-1.28830900	0.14348900
H	-0.91962300	-1.72906600	1.09151500
H	-0.50807400	-0.21073700	0.11532800
C	-0.34247100	-2.07806400	-0.96488400
H	-1.46320800	-2.03102300	-1.42795200
H	0.24125800	-1.63348900	-1.76893200
H	-0.18694800	-3.14261300	-0.79527800

TS9

O	-4.32487400	3.29802000	1.76082800
O	-3.26192100	2.45724400	3.94120700
O	-7.33959100	-1.95739300	1.87010200
O	-7.72535000	0.57674700	1.88503800
O	-6.69857000	-0.54716100	3.96040000
O	-4.19163100	-3.41503300	3.80186700
O	-3.10219100	-1.42227200	4.99211700
O	-1.18909200	-5.70701100	3.88393100

O	-4.18709600	-3.42319000	-3.79180800
O	-3.09578300	-1.43343900	-4.98502800
O	-1.18433000	-5.71557400	-3.86484100
Si	-1.37066700	-7.28292100	3.81274700
Si	-1.36585400	-7.29130000	-3.79031200
O	2.70040500	-4.99119300	2.44687800
O	-2.91696400	-7.65866500	3.90888700
O	-0.86390900	-7.82865000	2.40396000
O	2.70348500	-4.99659000	-2.42441500
O	-2.91207100	-7.66725200	-3.88760700
O	-0.86091400	-7.83391700	-2.37973200
Si	2.82113100	-6.29175500	1.54297900
Si	0.41710000	-8.25314600	1.54983500
Si	2.82304800	-6.29512500	-1.51751300
Si	0.41902900	-8.25652900	-1.52306500
O	1.63382600	-7.28827000	1.89553500
O	4.19348600	-7.02405100	1.77744900
O	2.74196500	-5.81630900	0.01210800
O	-0.00729900	-8.14066600	0.01294200
O	1.63618800	-7.29241500	-1.86936200
O	4.19570000	-7.02793300	-1.74864800
O	-4.32279200	3.29408500	-1.76541300
O	-3.25701900	2.44854300	-3.94258500
O	-7.33719000	-1.96149100	-1.86682000
O	-7.72298100	0.57258800	-1.88778500
O	-6.69360200	-0.55593000	-3.95940300
Si	-3.65957500	-0.51211500	-6.14567300
Si	-3.09739900	2.42837600	-5.51704200
Si	-6.70772700	-0.61680200	-5.52585700
O	-2.99735200	0.93484400	-6.05011700
O	-5.24133000	-0.39140500	-6.07400500
O	-7.25028300	-2.01797500	-6.03565300
Si	-3.10433300	2.44053500	5.51584600
Si	-6.71467700	-0.60459400	5.52683800
Si	-3.66735200	-0.49871400	6.15033500
O	-3.00494900	0.94815400	6.05236300
O	-7.25792600	-2.00465000	6.03903500
O	-5.24893800	-0.37805500	6.07630200
Si	-5.69720000	4.03016400	1.52625400
Si	-8.10121000	2.06880600	1.53311000
Si	-5.69526600	4.02682800	-1.53423300
Si	-8.09933900	2.06542600	-1.53984800
O	-6.88448900	3.03368200	1.87882100
O	-5.81790300	5.33076000	2.43014900

O	-5.77634700	4.50564800	-0.00461300
O	-8.52563400	2.18128000	-0.00377600
O	-6.88212900	3.02953200	-1.88607600
O	-5.81485300	5.32538900	-2.44113600
Si	-7.32102900	-3.50404900	1.54547200
Si	-7.31912000	-3.50745500	-1.53890700
O	-6.17873900	-4.23827700	2.36488300
O	-7.03832700	-3.73857200	0.00368400
O	-6.17572900	-4.24352500	-2.35519000
Si	-5.38590800	-4.43889300	3.72708400
O	-4.81926400	-5.91984800	3.76647100
O	-6.37007400	-4.21324500	4.94563900
Si	-7.24480600	-3.58636700	6.10248800
Si	-4.36420200	-7.27683900	4.44235900
Si	-5.38118700	-4.44715300	-3.71597800
O	-4.81454500	-5.92812700	-3.75145600
O	-6.36391200	-4.22408300	-4.93623800
Si	-7.23715200	-3.59980400	-6.09560300
Si	-4.35864000	-7.28660200	-4.42373200
O	5.70276800	-2.94164800	3.76980000
O	5.70749700	-2.94993700	-3.74809300
O	6.60029800	0.58242100	3.88188300
O	6.60516100	0.57388500	-3.86681300
O	5.58650900	3.65024100	1.88596500
O	5.58887100	3.64609200	-1.87893300
O	2.55540400	5.85100500	1.80203400
O	2.55766000	5.84703000	-1.80365500
O	-1.42023800	5.72379200	2.35689100
O	-1.41730100	5.71856000	-2.36323300
Si	-6.17860300	5.96578400	3.84403300
Si	-6.17377100	5.95730300	-3.85686100
Si	-1.46896600	6.54293400	3.71745000
Si	-1.46428400	6.53474700	-3.72563200
Si	6.79074900	4.62623900	1.53344600
Si	6.79266800	4.62286800	-1.52705100
Si	2.79025500	7.38267100	1.52450000
Si	2.79217400	7.37930400	-1.52920300
O	-5.41184300	7.36192000	3.96837600
O	-5.40685400	7.35316000	-3.98331600
O	-0.23725900	7.52129000	3.79234800
O	-2.81555000	7.38030400	3.75308500
O	-0.23249300	7.51292500	-3.80114600
O	-2.81083100	7.37202400	-3.76480500
Si	-4.06262900	8.08584800	4.42562800

Si	-4.05706400	8.07608500	-4.44046600
O	4.29877700	7.76174000	1.83282500
O	1.81649300	8.30996300	2.40176700
O	2.47692200	7.71971900	-0.00296900
O	4.30108300	7.75769700	-1.83646900
O	1.81951400	8.30466300	-2.40973000
Si	0.99313000	8.49681300	3.75084400
Si	0.99784200	8.48853900	-3.76024700
O	6.33175400	6.11305000	1.76431900
O	8.04333800	4.26269700	2.44003200
O	7.24497200	4.45535800	0.00361700
O	6.33396600	6.10916400	-1.76177900
O	8.04639300	4.25733300	-2.43126500
Si	5.76039300	7.45225900	2.37998500
Si	5.76337900	7.44701300	-2.38111200
Si	8.59615300	3.79079800	3.85570000
Si	8.60098800	3.78231700	-3.84519500
Si	8.06600000	0.80907100	4.43135000
Si	8.07155000	0.79932600	-4.41494100
O	8.60920300	2.20910800	3.91915000
O	8.61411300	2.20049000	-3.90514500
O	9.02594700	-0.31537800	3.81669100
O	9.03072300	-0.32376500	-3.79660100
Si	9.51151200	-1.82521600	3.76755900
Si	9.51622800	-1.83348900	-3.74353500
O	8.28106200	-2.80066200	3.80906500
O	8.28582400	-2.80902500	-3.78443200
Si	7.04934700	-3.77902100	3.73416700
Si	7.05402100	-3.78721300	-3.70892700
O	7.09808300	-4.59815800	2.37359900
O	7.10104500	-4.60335800	-2.34649700
Si	5.39906300	-7.83787000	2.39660100
Si	5.40205000	-7.84311200	-2.36449300
Si	7.60362700	-5.86066500	1.55740600
Si	7.60556200	-5.86406400	-1.52689200
O	6.76863500	-7.15603500	1.95030900
O	6.77106300	-7.16029900	-1.91798700
O	7.42938500	-5.54149600	0.01474300
H	-5.24783700	-8.37875200	-4.00250500
H	-5.25287400	-8.36991200	4.02242300
H	-8.63970200	-4.03554700	5.98673100
H	-8.64357500	-4.03384400	1.90749700
H	-8.64117700	-4.03804600	-1.90141900
H	-8.63219400	-4.04872600	-5.98060400

H	-6.72485400	-4.03859700	-7.40170700
H	-6.73414600	-4.02228100	7.41020000
H	-7.59590600	0.42775500	6.09115100
H	-7.58832400	0.41428300	-6.09354400
H	-9.28028500	2.45952400	2.31927700
H	-9.27735600	2.45440900	-2.32829000
H	-7.61848700	6.25645800	3.90010600
H	-7.61358400	6.24785100	-3.91537900
H	-5.75591200	5.04731500	-4.93306400
H	-4.15464300	9.46646400	3.92929400
H	-3.91627200	8.07026100	5.88823900
H	-4.14970100	9.45778700	-3.94729200
H	-3.90887600	8.05727300	-5.90285600
H	-5.76209500	5.05817200	4.92276300
H	-4.25479800	3.08229900	-6.14454100
H	-1.85572000	3.09337600	-5.93758100
H	-4.26248200	3.09588200	6.14045100
H	-1.86314500	3.10641000	5.93648000
H	-1.43838400	5.60772700	4.85118900
H	-1.43229400	5.59703300	-4.85727300
H	1.86399700	8.23339700	4.90544800
H	0.54332600	9.89556400	3.79636600
H	0.54809900	9.88718500	-3.80941500
H	1.87015800	8.22258200	-4.91317300
H	2.10018100	4.51256600	3.85383600
H	2.10502200	4.50407900	-3.85307500
H	5.71294900	7.32013000	-3.84475500
H	6.62071400	8.56814100	-1.97007200
H	6.61824300	8.57247800	1.96755700
H	5.70812400	7.32859800	3.84384100
H	7.78233600	4.37401600	4.93201600
H	9.99105100	4.23997500	3.97145800
H	9.99602800	4.23123900	-3.96019100
H	7.78851900	4.36316000	-4.92381100
H	4.49472900	1.55965600	4.88151200
H	8.05096900	0.75515300	5.90028600
H	10.38234800	-2.08856700	4.92220100
H	10.27198200	-1.99773500	2.52143700
H	4.50084700	1.54891200	-4.87123600
H	4.60945800	-2.26468100	-5.88614100
H	10.38850900	-2.09938200	-4.89649600
H	10.27512900	-2.00326200	-2.49608000
H	4.60204800	-2.25168800	5.90495800
H	8.05836100	0.74217200	-5.88377000

H	7.08602400	-4.72491700	-4.84056100
H	9.01461400	-6.15394600	-1.82928200
H	9.01229900	-6.14988000	1.86220400
H	7.07993400	-4.71422400	4.86790400
H	5.42432700	-9.21897100	1.89379300
H	5.42668100	-9.22310600	-1.85861300
H	2.75622500	-5.26378400	4.93948200
H	2.76243200	-5.27467800	-4.91632700
H	0.76711400	-9.64274400	1.87755700
H	0.76944800	-9.64684900	-1.84728800
H	-0.63068600	-7.95781100	4.88877900
H	-0.62456100	-7.96855500	-4.86396300
H	-0.74747900	-4.31159200	5.90036400
H	-0.74000400	-4.32467400	-5.88378100
H	-4.35406700	-7.12736700	5.90470500
H	-4.34666800	-7.14035500	-5.88638900
H	-3.26382200	-1.05057900	7.45152700
H	-3.25441000	-1.06695300	-7.44522100
H	5.27015900	-7.87143300	-3.82829400
H	5.26533500	-7.86296300	3.86029200
Al	-3.50606400	-0.42886100	-1.49796600
Si	-2.85891200	-2.40831500	3.66070900
Si	-3.49712500	-0.45353800	1.49630200
Si	-2.98113800	2.42687500	2.27842000
Si	-6.56849600	-0.54408700	2.27711500
Si	-2.96317500	2.40841800	-2.27329800
Si	-2.86662700	-2.39536100	-3.61570200
Si	-6.56553400	-0.54169500	-2.27757000
O	-2.95398500	-1.76489300	2.22853000
O	-2.81601100	0.84858100	2.14505100
O	-2.82726700	-0.61190200	0.07415100
O	-5.10968100	-0.32956800	1.62891000
O	-5.19375600	-0.23484200	-1.57808600
O	-2.92422200	-1.89122500	-2.14158200
O	-2.61659300	0.88761400	-2.10077400
O	-1.40158500	-2.99167800	-3.81589800
O	-1.41032400	-3.02016300	3.77296300
O	0.80567300	-4.05054000	-3.86995700
O	0.82168800	-4.04770600	3.97715800
O	3.00519300	-2.88045400	-4.09153000
O	4.17574600	-0.68651600	-4.02649800
O	4.49868100	1.15492400	-2.33571900
O	2.95384800	3.18541800	-1.79622700
O	0.54092700	3.92427600	-2.12753700

O	-1.81336600	3.20626100	-1.47735200
O	-1.75841300	3.12530600	1.52752800
O	0.54073300	3.92219200	2.16072900
O	2.95157000	3.19137700	1.79251100
O	3.01364100	-2.86642300	4.08579400
O	4.17295300	-0.67246000	4.04748700
O	4.49156300	1.16236600	2.34703700
O	4.53464200	2.04997700	0.00290500
O	-0.48657000	4.99302000	0.01612400
Si	-0.66156600	-4.22507600	-4.46697500
Si	2.36561900	-4.32123800	-3.93122500
Si	-0.65686700	-4.22375900	4.48437900
Si	2.37313700	-4.31002400	3.95173800
Si	4.37616100	-2.17203400	4.50839200
Si	4.37843300	-2.18450500	-4.49102600
Si	4.90803500	0.70238300	-3.80534400
Si	4.90422800	0.71370700	3.81742100
Si	4.36877100	2.50575300	-1.51156700
Si	4.36814800	2.50917800	1.51501400
Si	2.05569300	4.34594600	2.44814300
Si	2.05088800	4.34024300	-2.44485800
Si	-0.81469000	4.41573100	1.46862900
Si	-0.81904900	4.43379700	-1.44913900
C	-0.78213200	-1.34542400	-0.20458300
H	-1.27332100	-1.71190000	-1.09341000
H	-0.86231400	-1.96337400	0.68438500
C	-0.10604800	-0.03246000	-0.12843700
H	-0.47707900	0.50972200	0.74448800
H	0.96490000	-0.20156700	0.02048500
H	-0.27770600	0.55891300	-1.02587100
O	0.83168900	-2.48863300	-0.97702200
H	1.16892700	-3.24221900	-0.47866300
H	0.63127200	-2.81915000	-1.86639500

TS10

O	-4.26656600	3.37248900	1.76352600
O	-3.21490300	2.51731400	3.94378100
O	-7.35337000	-1.84089100	1.87374300
O	-7.70418200	0.69832500	1.88855300
O	-6.69260800	-0.43946600	3.96381800
O	-4.22544100	-3.34161000	3.80505900
O	-3.10844000	-1.36395600	4.99496100
O	-1.25474300	-5.67472400	3.88676500

O	-4.22238000	-3.35039500	-3.78861600
O	-3.10397600	-1.37595500	-4.98218300
O	-1.25148900	-5.68393100	-3.86200700
Si	-1.45802100	-7.24798900	3.81573500
Si	-1.45468800	-7.25700100	-3.78732400
O	2.64398700	-5.01265900	2.44896300
O	-3.00933100	-7.60239000	3.91217900
O	-0.95908200	-7.80075100	2.40689900
O	2.64611800	-5.01846200	-2.42233000
O	-3.00595400	-7.61162500	-3.88431500
O	-0.95701700	-7.80641700	-2.37679300
Si	2.74662500	-6.31482800	1.54514300
Si	0.31580500	-8.24291700	1.55257800
Si	2.74794700	-6.31845300	-1.51534900
Si	0.31713600	-8.24655600	-1.52032200
O	1.54576800	-7.29486700	1.89798700
O	4.10880400	-7.06594100	1.77942400
O	2.67374100	-5.83845100	0.01424900
O	-0.10728000	-8.12471700	0.01575300
O	1.54739800	-7.29932600	-1.86690900
O	4.11033200	-7.07011700	-1.74667200
O	-4.26517000	3.36826200	-1.76271500
O	-3.21153600	2.50795700	-3.94001100
O	-7.35169600	-1.84530000	-1.86317900
O	-7.70254700	0.69385200	-1.88427100
O	-6.68918300	-0.44889400	-3.95598500
Si	-3.65523200	-0.44703900	-6.14280000
Si	-3.05249100	2.48547600	-5.51449600
Si	-6.70442600	-0.50968300	-5.52243200
O	-2.97312300	0.99066700	-6.04747400
O	-5.23516100	-0.30454800	-6.07085900
O	-7.26632300	-1.90328700	-6.03202300
Si	-3.05727800	2.49855400	5.51839300
Si	-6.70922400	-0.49655500	5.53026300
Si	-3.66061800	-0.43261400	6.15320800
O	-2.97836600	1.00498500	6.05500700
O	-7.27161600	-1.88895600	6.04266500
O	-5.24040600	-0.29018600	6.07944800
Si	-5.62871800	4.12344900	1.52914100
Si	-8.05951600	2.19539200	1.53657600
Si	-5.62737900	4.11985900	-1.53134600
Si	-8.05824300	2.19175800	-1.53638200
O	-6.82955800	3.14344200	1.88199600
O	-5.73133200	5.42565200	2.43295700

O	-5.70158200	4.59986400	-0.00174900
O	-8.48262600	2.31358800	-0.00024300
O	-6.82793000	3.13898000	-1.88290100
O	-5.72923100	5.41987500	-2.43832900
Si	-7.35617300	-3.38767900	1.54922900
Si	-7.35486400	-3.39134200	-1.53515000
O	-6.22395800	-4.13751200	2.36849300
O	-7.07700400	-3.62618900	0.00740900
O	-6.22186700	-4.14315300	-2.35158000
Si	-5.43372200	-4.34892800	3.73056800
O	-4.88752400	-5.83754400	3.76996800
O	-6.41446800	-4.10965300	4.94928200
Si	-7.28027400	-3.47069900	6.10623800
Si	-4.45107600	-7.20062400	4.44588000
Si	-5.43045000	-4.35780800	-3.71249400
O	-4.88426700	-5.84644800	-3.74795900
O	-6.41022700	-4.12131300	-4.93259600
Si	-7.27499400	-3.48515200	-6.09185300
Si	-4.44723900	-7.21112600	-4.42021100
O	5.67453400	-3.00456600	3.77119200
O	5.67780000	-3.01348100	-3.74670200
O	6.62054300	0.50681300	3.88284300
O	6.62389800	0.49763300	-3.86585300
O	5.64875200	3.58815800	1.88686800
O	5.65038100	3.58369600	-1.87802900
O	2.64823400	5.83046000	1.80330800
O	2.64978800	5.82618400	-1.80238000
O	-1.32868400	5.75806400	2.35888400
O	-1.32666600	5.75244000	-2.36124000
Si	-6.08299800	6.06569000	3.84685500
Si	-6.07966400	6.05656800	-3.85403800
Si	-1.36588000	6.57790200	3.71938800
Si	-1.36264600	6.56909500	-3.72369400
Si	6.86625800	4.54744900	1.53406000
Si	6.86758200	4.54382300	-1.52643700
Si	2.90411100	7.35872400	1.52561500
Si	2.90543600	7.35510400	-1.52808800
O	-5.29705600	7.45114100	3.97095400
O	-5.29361500	7.44171900	-3.98073800
O	-0.12079800	7.53920300	3.79399100
O	-2.70079500	7.43374400	3.75519900
O	-0.11751000	7.53020600	-3.79950300
O	-2.69753900	7.42483800	-3.76269100
Si	-3.93791500	8.15645000	4.42791000

Si	-3.93407500	8.14594800	-4.43818500
O	4.41776800	7.71700000	1.83364100
O	1.94337300	8.29940800	2.40298400
O	2.59517700	7.69994300	-0.00182500
O	4.41936000	7.71265200	-1.83565300
O	1.94545800	8.29370700	-2.40851300
Si	1.12290500	8.49768200	3.75219300
Si	1.12615500	8.48878300	-3.75889900
O	6.42782800	6.04045900	1.76490000
O	8.11388300	4.16675500	2.44045100
O	7.31781000	4.37021200	0.00416300
O	6.42935400	6.03627900	-1.76119800
O	8.11599100	4.16098500	-2.43084600
Si	5.87507900	7.38745700	2.38056500
Si	5.87713800	7.38181500	-2.38053200
Si	8.66039900	3.68739100	3.85605600
Si	8.66373600	3.67826900	-3.84483900
Si	8.08932700	0.71329300	4.43203000
Si	8.09315500	0.70281200	-4.41426000
O	8.65167200	2.10567600	3.91962600
O	8.65505900	2.09640700	-3.90466900
O	9.03358300	-0.42432000	3.81728800
O	9.03687800	-0.43334000	-3.79600500
Si	9.49829500	-1.94070700	3.76818600
Si	9.50155000	-1.94960500	-3.74290800
O	8.25453300	-2.89910600	3.80998600
O	8.25781700	-2.90810100	-3.78351100
Si	7.00944400	-3.86041100	3.73538400
Si	7.01267000	-3.86922300	-3.70771100
O	7.04664800	-4.68024300	2.37486900
O	7.04869100	-4.68583600	-2.34522700
Si	5.30316700	-7.89624200	2.39842500
Si	5.30522900	-7.90188100	-2.36266900
Si	7.53460800	-5.94965600	1.55868400
Si	7.53594300	-5.95331100	-1.52561400
O	6.68192200	-7.23337200	1.95183600
O	6.68359800	-7.23795700	-1.91646000
O	7.36450300	-5.62823200	0.01602700
H	-5.35132100	-8.29089200	-3.99874100
H	-5.35479600	-8.28138400	4.02618600
H	-8.68124600	-3.90063000	5.99076400
H	-8.68582700	-3.89917800	1.91153000
H	-8.68416900	-3.90369700	-1.89738500
H	-8.67606600	-3.91480500	-5.97657100

H	-6.76902200	-3.93105700	-7.39801400
H	-6.77543300	-3.91350800	7.41389200
H	-7.57604800	0.54787800	6.09465300
H	-7.57083800	0.53339200	-6.09004100
H	-9.23295600	2.60237500	2.32292300
H	-9.23093100	2.59687300	-2.32464400
H	-7.51873000	6.37617500	3.90316300
H	-7.51534800	6.36691700	-3.91232200
H	-5.67457200	5.14083000	-4.93024600
H	-4.01099100	9.53816500	3.93148600
H	-3.79152400	8.13895700	5.89049600
H	-4.00758200	9.52883300	-3.94510100
H	-3.78642300	8.12498800	-5.90059900
H	-5.67883700	5.15250700	4.92558200
H	-4.20088500	3.15523300	-6.14183900
H	-1.80184500	3.13327700	-5.93530800
H	-4.20617800	3.16983800	6.14315300
H	-1.80696000	3.14729900	5.93875300
H	-1.34798000	5.64244700	4.85319400
H	-1.34377800	5.63094500	-4.85526800
H	1.99026800	8.22238100	4.90666200
H	0.69241900	9.90250000	3.79768700
H	0.69571200	9.89348900	-3.80809400
H	1.99451700	8.21075000	-4.91195900
H	2.17498600	4.49857100	3.85529500
H	2.17832700	4.48944300	-3.85161600
H	5.82470400	7.25552900	-3.84415700
H	6.74991100	8.49105700	-1.96973200
H	6.74820600	8.49572200	1.96789700
H	5.82137400	7.26463700	3.84444000
H	7.85488600	4.28184400	4.93247300
H	10.06137400	4.11731900	3.97153100
H	10.06480700	4.10792300	-3.96011800
H	7.85915100	4.27016800	-4.92335500
H	4.52881500	1.51303300	4.88277200
H	8.07381800	0.65969700	5.90097400
H	10.36562800	-2.21594300	4.92269200
H	10.25609300	-2.12377800	2.52194200
H	4.53303500	1.50147800	-4.86997700
H	4.58892100	-2.31332600	-5.88460700
H	10.36987800	-2.22757400	-4.89600500
H	10.25826400	-2.12972300	-2.49557600
H	4.58380600	-2.29935000	5.90649300
H	8.07891700	0.64573500	-5.88308200

H	7.03155100	-4.80736400	-4.83927800
H	8.94081300	-6.26259900	-1.82823300
H	8.93921700	-6.25822600	1.86325300
H	7.02735000	-4.79586200	4.86918700
H	5.30931500	-9.27759800	1.89571800
H	5.31093900	-9.28204600	-1.85668700
H	2.69649400	-5.28580700	4.94157800
H	2.70078300	-5.29752200	-4.91423100
H	0.64670300	-9.63718000	1.88034600
H	0.64831200	-9.64159500	-1.84450000
H	-0.72721400	-7.93292700	4.89168700
H	-0.72298800	-7.94448300	-4.86105500
H	-0.79358900	-4.28537000	5.90301100
H	-0.78840900	-4.29943400	-5.88113400
H	-4.43862000	-7.05119700	5.90821200
H	-4.43351600	-7.06516600	-5.88288200
H	-3.26449500	-0.98988800	7.45437100
H	-3.25798100	-1.00750300	-7.44237700
H	5.17269700	-7.92849200	-3.82644400
H	5.16936900	-7.91938300	3.86214200
Al	-3.44722800	-0.35267100	-1.49582700
Si	-2.87638800	-2.35658100	3.66982000
Si	-3.50530900	-0.38106900	1.49723800
Si	-2.93139400	2.48264300	2.28026200
Si	-6.56751900	-0.43741300	2.28700200
Si	-2.90743800	2.47708800	-2.27098600
Si	-2.87067700	-2.35757600	-3.64451500
Si	-6.56654200	-0.44161600	-2.28566600
O	-2.99708700	-1.71842900	2.23230700
O	-2.79398000	0.90262400	2.14032500
O	-2.90752200	-0.61430000	0.07544900
O	-5.11535300	-0.22375500	1.64421800
O	-5.12645400	-0.25191500	-1.70001100
O	-2.72812900	-1.82901700	-2.13713100
O	-2.55973800	0.94936200	-2.10961700
O	-1.44520000	-2.98573600	-3.87842800
O	-1.43801600	-2.98571800	3.76868800
O	0.77933000	-4.03888400	-3.92130000
O	0.78216500	-4.03594800	3.99131000
O	2.98413400	-2.89966200	-4.07504500
O	4.17796000	-0.72477600	-4.03171000
O	4.52336800	1.10823200	-2.33520000
O	3.01024600	3.16051900	-1.79235300
O	0.60578800	3.93355100	-2.13229900

O	-1.76340500	3.26547500	-1.46743700
O	-1.70960100	3.17581800	1.52668800
O	0.60683500	3.92991200	2.17095800
O	3.00959900	3.16907500	1.78876000
O	2.98780900	-2.89200600	4.08867800
O	4.17684400	-0.71389500	4.05255000
O	4.51984900	1.11640500	2.34959500
O	4.58102300	2.00193100	0.00436000
O	-0.39011900	5.01770300	0.02220900
Si	-0.68147200	-4.22572500	-4.48210100
Si	2.32999800	-4.33639500	-3.92467800
Si	-0.69942500	-4.19981100	4.48479100
Si	2.32819800	-4.32689100	3.95196400
Si	4.35850800	-2.21721300	4.51010100
Si	4.36217500	-2.22782600	-4.48885400
Si	4.92941700	0.65147300	-3.80409900
Si	4.92592400	0.66090300	3.81942300
Si	4.41552700	2.45948400	-1.50864800
Si	4.41519800	2.46283100	1.51612300
Si	2.12937900	4.33126900	2.45009300
Si	2.12428600	4.32578300	-2.44322100
Si	-0.73697400	4.44238000	1.47291300
Si	-0.73738000	4.46762200	-1.44148000
C	-0.14555700	-2.21406700	-0.31996000
H	0.21231700	-3.23726100	-0.35797600
H	-0.46814100	-1.84169400	0.64544900
C	-0.17857300	-1.41691100	-1.43314600
H	-1.41187500	-1.78274000	-1.65505800
H	-0.35241200	-0.34926400	-1.31669400
H	0.31732700	-1.75704300	-2.33899500
O	-2.17014300	-3.56489700	0.08548000
H	-2.51405200	-2.99302600	0.78578300
H	-2.73900700	-3.35931600	-0.66516900

TS11

O	4.10313700	3.58183300	-1.75580400
O	3.08643900	2.68615400	-3.93638100
O	7.39126400	-1.50699800	-1.86469700
O	7.64262900	1.04398300	-1.87951100
O	6.67699200	-0.13252700	-3.95506900
O	4.32501400	-3.12882000	-3.79702200
O	3.13202900	-1.19635000	-4.98740700
O	1.44774600	-5.57619700	-3.87964000

O	4.31965900	-3.13744900	3.79665200
O	3.12457000	-1.20816000	4.98973600
O	1.44216100	-5.58524800	3.86913000
Si	1.71229800	-7.14031800	-3.80846200
Si	1.70667600	-7.14918300	3.79459500
O	-2.47436900	-5.06687500	-2.44321900
O	3.27630000	-7.43385600	-3.90435300
O	1.23484100	-7.71209700	-2.39977200
O	-2.47796600	-5.07258300	2.42807300
O	3.27057700	-7.44293900	3.89214000
O	1.23133600	-7.71767000	2.38391900
Si	-2.52637900	-6.37202700	-1.53937100
Si	-0.02209800	-8.20369400	-1.54587100
Si	-2.52862200	-6.37559200	1.52112100
Si	-0.02435500	-8.20727300	1.52702900
O	-1.28803500	-7.30442300	-1.89175200
O	-3.85809800	-7.17578300	-1.77408700
O	-2.47269100	-5.89311200	-0.00847600
O	0.39551200	-8.06900300	-0.00890600
O	-1.29079800	-7.30880900	1.87314300
O	-3.86068700	-7.17989000	1.75200900
O	4.10068200	3.57767900	1.77043700
O	3.08070100	2.67695300	3.94741000
O	7.38846600	-1.51133700	1.87222500
O	7.63985900	1.03958300	1.89331300
O	6.67118500	-0.14180000	3.96473200
Si	3.63871700	-0.25837900	6.15049800
Si	2.92210600	2.64833200	5.52184100
Si	6.68824700	-0.20189200	5.53118800
O	2.90100400	1.15158300	6.05486500
O	5.21189900	-0.05428500	6.07909600
O	7.30397300	-1.57246600	6.04104100
Si	2.93021400	2.66119500	-5.51104600
Si	6.69637000	-0.18898000	-5.52150600
Si	3.64781000	-0.24419200	-6.14550800
O	2.90989000	1.16566400	-6.04761500
O	7.31290000	-1.55836900	-6.03364400
O	5.22080300	-0.04016100	-6.07120900
Si	5.43483400	4.38543600	-1.52098500
Si	7.93909300	2.55379900	-1.52748400
Si	5.43257300	4.38190500	1.53950200
Si	7.93689400	2.55022800	1.54547400
O	6.67316200	3.45307000	-1.87337600
O	5.48681900	5.69062000	-2.42483000

O	5.48850200	4.86438800	0.00990700
O	8.35672900	2.68848700	0.00947500
O	6.67040000	3.44868100	1.89152100
O	5.48325200	5.68493900	2.44645600
Si	7.45437100	-3.05248400	-1.54010600
Si	7.45213500	-3.05608600	1.54427200
O	6.35259500	-3.84600000	-2.35972500
O	7.18419600	-3.30166300	0.00162900
O	6.34908600	-3.85155100	2.36034800
Si	5.57169300	-4.08817100	-3.72206300
O	5.08407400	-5.59698800	-3.76158000
O	6.54276900	-3.81081300	-4.94044900
Si	7.38335800	-3.13856900	-6.09713700
Si	4.70143600	-6.97609900	-4.43757400
Si	5.56618300	-4.09690700	3.72099800
O	5.07855400	-5.60574500	3.75634700
O	6.53555100	-3.82227800	4.94142700
Si	7.37440700	-3.15278300	6.10095200
Si	4.69493100	-6.98642700	4.42851500
O	-5.58058100	-3.17873800	-3.76659500
O	-5.58611000	-3.18750600	3.75129700
O	-6.66298900	0.29300600	-3.87874700
O	-6.66867600	0.28397700	3.86994800
O	-5.81299400	3.41002900	-1.88258600
O	-5.81575600	3.40564100	1.88230900
O	-2.90238200	5.76782700	-1.79809800
O	-2.90502100	5.76362100	1.80758900
O	1.07452100	5.85080900	-2.35229500
O	1.07108400	5.84527800	2.36782800
Si	5.81370800	6.34385600	-3.83863600
Si	5.80805600	6.33488400	3.86225500
Si	1.08013900	6.67142600	-3.71282700
Si	1.07466400	6.66276300	3.73025400
Si	-7.06716400	4.32104300	-1.53024700
Si	-7.06941000	4.31747700	1.53025000
Si	-3.21785600	7.28493800	-1.52057000
Si	-3.22010000	7.28137900	1.53313300
O	4.97429100	7.69754600	-3.96307600
O	4.96845700	7.68827900	3.98861500
O	-0.20151700	7.58335500	-3.78790800
O	2.38061700	7.57875700	-3.74821800
O	-0.20709000	7.57450700	3.80558500
O	2.37509800	7.56999700	3.76967100
Si	3.58879700	8.34921000	-4.42053600

Si	3.58228800	8.33888200	4.44555700
O	-4.74424500	7.58380500	-1.82913700
O	-2.29429000	8.26240100	-2.39765200
O	-2.92301700	7.63801900	0.00696100
O	-4.74694100	7.57952900	1.84015700
O	-2.29782300	8.25679400	2.41384400
Si	-1.48172500	8.49252500	-3.74658700
Si	-1.48723500	8.48377200	3.76450400
O	-6.68730800	5.83003100	-1.76100800
O	-8.29865100	3.89187400	-2.43705100
O	-7.51198000	4.12635800	-0.00049800
O	-6.68989500	5.82591900	1.76508900
O	-8.30222600	3.88619900	2.43424600
Si	-6.18738200	7.19756900	-2.37654900
Si	-6.19087300	7.19202100	2.38454800
Si	-8.82553400	3.39147800	-3.85282100
Si	-8.83119000	3.38250600	3.84807200
Si	-8.13852700	0.44193600	-4.42845200
Si	-8.14501600	0.43162800	4.41783700
O	-8.75500900	1.81130900	-3.91631100
O	-8.76075000	1.80219300	3.90798300
O	-9.03783900	-0.73167100	-3.81398100
O	-9.04342400	-0.74054200	3.79931100
Si	-9.44298100	-2.26505000	-3.76496500
Si	-9.44849700	-2.27380100	3.74612800
O	-8.16271800	-3.17413800	-3.80628700
O	-8.16828700	-3.18298400	3.78720900
Si	-6.88105500	-4.08607100	-3.73120800
Si	-6.88652100	-4.09473800	3.71188600
O	-6.88668100	-4.90668300	-2.37066600
O	-6.89014400	-4.91218300	2.34943100
Si	-5.01890100	-8.05212600	-2.39346000
Si	-5.02239600	-8.05767200	2.36763300
Si	-7.32496700	-6.19415900	-1.55458600
Si	-7.32723100	-6.19775300	1.52971100
O	-6.42265200	-7.44360200	-1.94738000
O	-6.42549200	-7.44811000	1.92091500
O	-7.16808300	-5.86628000	-0.01188600
H	5.64064700	-8.03007000	4.00741100
H	5.64653700	-8.02071900	-4.01751500
H	8.80001400	-3.51344300	-5.98115700
H	8.80311700	-3.51166900	-1.90192200
H	8.80031300	-3.51611300	1.90699200
H	8.79123200	-3.52738400	5.98617600

H	6.88578400	-3.61806500	7.40695900
H	6.89665300	-3.60080500	-7.40494400
H	7.52193200	0.88849500	-6.08564700
H	7.51305600	0.87425100	6.09904500
H	9.09601300	3.00628000	-2.31344400
H	9.09258900	3.00086800	2.33412100
H	7.23623500	6.71018400	-3.89446400
H	7.23050100	6.70107900	3.92102100
H	5.43866900	5.40406000	4.93836800
H	3.60767300	9.73274300	-3.92415500
H	3.44370800	8.32596100	-5.88317200
H	3.60189300	9.72356500	3.95243100
H	3.43505900	8.31222300	5.90792100
H	5.44590000	5.41554500	-4.91745900
H	4.04324500	3.36245800	6.14954900
H	1.64696500	3.24680200	5.94218900
H	4.05223400	3.37682200	-6.13544100
H	1.65565700	3.26059100	-5.93187100
H	1.09918500	5.73594500	-4.84659200
H	1.09206100	5.72463300	4.86186800
H	-2.33727000	8.18351200	-4.90134200
H	-1.10642400	9.91308400	-3.79200100
H	-1.11200700	9.90422300	3.81377900
H	-2.34447600	8.17207400	4.91727700
H	-2.37675700	4.45536600	-3.84985600
H	-2.38241800	4.44638900	3.85705300
H	-6.13405500	7.06793200	3.84819700
H	-7.10616600	8.26631100	1.97339100
H	-7.10327600	8.27089900	-1.96423700
H	-6.12841200	7.07688900	-3.84039800
H	-8.04348200	4.01690300	-4.92898800
H	-10.24219300	3.76635100	-3.96880200
H	-10.24801400	3.75711000	3.96284600
H	-8.05071300	4.00542000	4.92683800
H	-4.61181300	1.38012700	-4.87800100
H	-8.12042500	0.38893500	-5.89738700
H	-10.29849900	-2.57399600	-4.91975700
H	-10.19348300	-2.47753800	-2.51897500
H	-4.61896900	1.36876300	4.87474600
H	-4.52615400	-2.44527700	5.88954400
H	-10.30570600	-2.58543500	4.89893800
H	-10.19716400	-2.48338500	2.49854300
H	-4.51749100	-2.43153100	-5.90155400
H	-8.12907100	0.37520300	5.88666700

H	-6.86913700	-5.03286100	4.84349300
H	-8.71905300	-6.56166900	1.83185900
H	-8.71634500	-6.55736800	-1.85962700
H	-6.86201400	-5.02154700	-4.86497100
H	-4.97126200	-9.43265000	-1.89068800
H	-4.97401600	-9.43702500	1.86171700
H	-2.51530000	-5.34195400	-4.93583900
H	-2.52255400	-5.35347600	4.91996800
H	-0.29816800	-9.60983000	-1.87368500
H	-0.30089800	-9.61417100	1.85116100
H	1.00917700	-7.85331800	-4.88463400
H	1.00201600	-7.86468300	4.86810600
H	0.93337500	-4.20598800	-5.89611400
H	0.92465200	-4.21982500	5.88802900
H	4.68366200	-6.82732500	-5.89991900
H	4.67500900	-6.84106300	5.89117500
H	3.27421000	-0.81655900	-7.44678100
H	3.26321000	-0.83388500	7.44996400
H	-4.88943500	-8.07903200	3.83145600
H	-4.88379200	-8.07007500	-3.85713000
Al	3.47316400	-0.26614900	1.41848400
Si	2.92841800	-2.17140500	-3.65192800
Si	3.51184000	-0.18134500	-1.48464700
Si	2.80262500	2.65388600	-2.23286600
Si	6.56993000	-0.13511600	-2.29659800
Si	2.80241100	2.59212100	2.24849600
Si	2.92306800	-2.19844400	3.65339400
Si	6.55394400	-0.13676300	2.29958500
O	2.72635200	-1.07488600	-2.53777400
O	2.79695500	1.24625200	-1.49069300
O	3.29112800	-0.96760800	-0.13295100
O	5.07753800	0.02363100	-1.79399800
O	5.10832500	0.02527000	1.74018800
O	2.74754000	-1.67469900	2.15959200
O	2.54688800	1.08677800	1.84662300
O	1.52287100	-2.88220300	3.89138200
O	1.55531200	-2.93530300	-3.72292600
O	-0.65104500	-4.04106500	3.91950600
O	-0.64835700	-4.01532400	-3.97821400
O	-2.89829600	-2.96890100	4.08505500
O	-4.17783900	-0.84192300	4.03834500
O	-4.59512100	0.97314800	2.33972300
O	-3.16637100	3.08628700	1.79923700
O	-0.79740300	3.94920400	2.13076000

O	1.59705400	3.36760900	1.52835200
O	1.49776500	3.32683700	-1.64432200
O	-0.84224800	3.95940400	-2.11215600
O	-3.19122200	3.06643200	-1.82841000
O	-2.90080900	-2.96297900	-4.08101500
O	-4.16560100	-0.82903800	-4.06047100
O	-4.62197800	0.95084000	-2.32947700
O	-4.68779700	1.86238700	0.00342500
O	0.19357800	5.08283900	-0.00008200
Si	0.81290800	-4.15079300	4.49130800
Si	-2.19246900	-4.37873500	3.93153500
Si	0.83981900	-4.12686600	-4.47730500
Si	-2.18220100	-4.36846500	-3.94732100
Si	-4.29562100	-2.34086200	-4.50606700
Si	-4.30248600	-2.35028800	4.49455100
Si	-4.98139400	0.50392000	3.80930800
Si	-4.97670300	0.50841200	-3.81680300
Si	-4.53948500	2.32861000	1.51509900
Si	-4.55780500	2.31091700	-1.51376000
Si	-2.36091900	4.28337900	-2.44798700
Si	-2.32440000	4.28462100	2.44868600
Si	0.49502300	4.53840000	-1.46866800
Si	0.53703300	4.53031900	1.46229900
C	0.54211000	-2.59139000	-0.27537400
H	0.33670600	-3.64746400	-0.40951100
H	0.81777500	-2.04696200	-1.17276600
C	0.40115700	-1.96827100	0.94785200
H	1.58258700	-1.98318400	1.42380800
H	0.29695300	-0.88296200	0.93539600
H	-0.12540200	-2.50273100	1.73888300
O	2.76588000	-3.44915600	-0.92104900
H	3.11850900	-2.65584300	-0.47544400
C	3.37281800	-4.62018600	-0.37884200
H	3.32022500	-5.38782300	-1.15652500
H	4.43378700	-4.42773400	-0.17232800
C	2.65756400	-5.08663500	0.87454800
H	3.13950500	-5.97581700	1.29454500
H	2.65959300	-4.30332400	1.63709800
H	1.61803000	-5.34795400	0.65390300

TS12

O	4.36485600	3.27907400	-1.75922000
O	3.29519500	2.44082500	-3.93729400

O	7.35085600	-1.99279400	-1.86356300
O	7.75036900	0.53919100	-1.88269900
O	6.71543700	-0.58223400	-3.95534300
O	4.19311100	-3.43621900	-3.79001100
O	3.11331400	-1.43933600	-4.98218900
O	1.17807100	-5.71192600	-3.86563700
O	4.19606400	-3.43298500	3.80344100
O	3.11683100	-1.43552700	4.99487900
O	1.18090500	-5.70879600	3.88305500
Si	1.35114800	-7.28869500	-3.79227000
Si	1.35392700	-7.28562400	3.81083100
O	-2.70604500	-4.97282100	-2.42582000
O	2.89528100	-7.67298400	-3.88937800
O	0.84283600	-7.82954300	-2.38216200
O	-2.70426400	-4.97085300	2.44548000
O	2.89813300	-7.66983300	3.90712200
O	0.84458600	-7.82761100	2.40153500
Si	-2.83293400	-6.27134600	-1.51984800
Si	-0.43960600	-8.24578500	-1.52612800
Si	-2.83181600	-6.27011000	1.54065100
Si	-0.43848200	-8.24454200	1.54677300
O	-1.65141200	-7.27482900	-1.87207600
O	-4.20947900	-6.99652300	-1.75185300
O	-2.74965900	-5.79403800	0.01022700
O	-0.01306700	-8.13330700	0.01017200
O	-1.65003600	-7.27330600	1.89282400
O	-4.20819300	-6.99509700	1.77424700
O	4.36617700	3.28039300	1.76701200
O	3.29803800	2.44406600	3.94653900
O	7.35220200	-1.99128200	1.87349400
O	7.75181100	0.54073300	1.89024000
O	6.71838700	-0.57903500	3.96456300
Si	3.68681700	-0.51548900	6.15364200
Si	3.13997100	2.42707000	5.52115500
Si	6.73368200	-0.63768200	5.53098000
O	3.03228000	0.93489600	6.05660000
O	5.26908300	-0.40346000	6.08022300
O	7.26912800	-2.04100600	6.04235000
Si	3.13593500	2.42262300	-5.51174300
Si	6.72965600	-0.64210600	-5.52172200
Si	3.68233800	-0.52058600	-6.14235100
O	3.02790600	0.93000200	-6.04591100
O	7.26478000	-2.04585900	-6.03234600
O	5.26462500	-0.40842000	-6.07007500

Si	5.74138800	4.00412000	-1.52711700
Si	8.13469900	2.02971300	-1.53340500
Si	5.74249000	4.00540000	1.53336300
Si	8.13582200	2.03095800	1.53948600
O	6.92288900	3.00067200	-1.87936700
O	5.86825600	5.30268300	-2.43310100
O	5.82464800	4.48146500	0.00294100
O	8.56124400	2.14219200	0.00288200
O	6.92427200	3.00218900	1.88553700
O	5.87004400	5.30464800	2.43818900
Si	7.32423200	-3.53882600	-1.53659000
Si	7.32534700	-3.53757700	1.54772400
O	6.17713600	-4.26806800	-2.35375900
O	7.04178100	-3.76949500	0.00583000
O	6.17885600	-4.26617000	2.36634500
Si	5.38186900	-4.46641600	-3.71487000
O	4.80714600	-5.94432300	-3.75147400
O	6.36603700	-4.24794100	-4.93473500
Si	7.24299900	-3.62757400	-6.09340300
Si	4.34404100	-7.29983200	-4.42485800
Si	5.38454300	-4.46346800	3.72820400
O	4.80989200	-5.94128200	3.76643000
O	6.36965600	-4.24394300	4.94716600
Si	7.24746200	-3.62264700	6.10469600
Si	4.34728300	-7.29624800	4.44123900
O	-5.69854200	-2.90896900	-3.74886000
O	-5.69579100	-2.90593100	3.76904000
O	-6.57701600	0.61975600	-3.86536500
O	-6.57418200	0.62288700	3.88333500
O	-5.54457300	3.68501300	-1.87508000
O	-5.54319700	3.68653100	1.88981900
O	-2.50147300	5.86938600	-1.79747300
O	-2.50015400	5.87084200	1.80822500
O	1.47286300	5.71972800	-2.35607100
O	1.47459200	5.72163700	2.36403000
Si	6.23098800	5.93361200	-3.84829300
Si	6.23380400	5.93672700	3.85260500
Si	1.52469200	6.53654500	-3.71791700
Si	1.52741600	6.53955200	3.72518300
Si	-6.74313800	4.66807200	-1.52283900
Si	-6.74201700	4.66930700	1.53766000
Si	-2.72771200	7.40272200	-1.52201100
Si	-2.72659300	7.40395400	1.53168700
O	5.47170400	7.33370800	-3.97398300

O	5.47461300	7.33692100	3.97771500
O	0.29824000	7.52147300	-3.79306600
O	2.87576800	7.36653000	-3.75614600
O	0.30101700	7.52454100	3.80043300
O	2.87851600	7.36956500	3.76175100
Si	4.12598900	8.06427500	-4.43098700
Si	4.12922900	8.06785900	4.43511300
O	-4.23446000	7.78952300	-1.82941600
O	-1.74980300	8.32338900	-2.40164000
O	-2.41102700	7.74035600	0.00463500
O	-4.23312200	7.79100600	1.83988300
O	-1.74804400	8.32533200	2.40985800
Si	-0.92678600	8.50373200	-3.75181400
Si	-0.92403800	8.50676600	3.75928200
O	-6.27629800	6.15201700	-1.75640800
O	-7.99858900	4.30998500	-2.42763500
O	-7.19675600	4.50196000	0.00769500
O	-6.27500800	6.15343900	1.76969000
O	-7.99680800	4.31195300	2.44366500
Si	-5.69828400	7.48717300	-2.37465900
Si	-5.69654100	7.48909500	2.38644100
Si	-8.55537600	3.83897000	-3.84204100
Si	-8.55256300	3.84208400	3.85885700
Si	-8.04201100	0.85354500	-4.41372100
Si	-8.03877600	0.85711700	4.43257700
O	-8.57708600	2.25728200	-3.90309300
O	-8.57422400	2.26044300	3.92120400
O	-9.00744200	-0.26474500	-3.79641800
O	-9.00465600	-0.26167000	3.81688100
Si	-9.50115700	-1.77184500	-3.74452900
Si	-9.49841300	-1.76881100	3.76657000
O	-8.27606900	-2.75402700	-3.78578200
O	-8.27328800	-2.75095900	3.80772100
Si	-7.04961300	-3.73895500	-3.71063000
Si	-7.04689400	-3.73594600	3.73247000
O	-7.10144300	-4.55577000	-2.34877900
O	-7.09971600	-4.55386300	2.37131800
Si	-5.42008100	-7.80470200	-2.36858300
Si	-5.41834000	-7.80277700	2.39251500
Si	-7.61302700	-5.81428400	-1.53018500
Si	-7.61189900	-5.81303800	1.55411400
O	-6.78548200	-7.11476100	-1.92196200
O	-6.78406700	-7.11320100	1.94633600
O	-7.43551300	-5.49374800	0.01182000

H	5.23011200	-8.39384900	4.02077800
H	5.22717700	-8.39709000	-4.00415500
H	8.63555100	-4.08415600	-5.97835000
H	8.64350600	-4.07635000	-1.89913300
H	8.64490200	-4.07481100	1.90976400
H	8.63992800	-4.07932000	5.98899300
H	6.73409000	-4.05668600	7.41196900
H	6.72867300	-4.06267000	-7.39994800
H	7.61592200	0.38459400	-6.08846500
H	7.62043700	0.38946600	6.09624000
H	9.31508400	2.41283900	-2.32133300
H	9.31678900	2.41471000	2.32622400
H	7.67237400	6.21636900	-3.90623300
H	7.67523200	6.21952900	3.90925700
H	5.81207900	5.03064000	4.93059200
H	4.22599900	9.44511400	-3.93682700
H	3.97808700	8.04728700	-5.89342800
H	4.22888100	9.44829500	3.93976400
H	3.98240200	8.05205100	5.89767400
H	5.80847500	5.02665900	-4.92524100
H	4.30152600	3.07562700	6.14651800
H	1.90233400	3.09945100	5.94191800
H	4.29700300	3.07072400	-6.13848200
H	1.89796100	3.09459800	-5.93215000
H	1.48789100	5.59982000	-4.85021300
H	1.49144100	5.60374400	4.85825800
H	-1.80022400	8.24332100	-4.90515500
H	-0.46943100	9.89994700	-3.79989000
H	-0.46665100	9.90302100	3.80589600
H	-1.79663300	8.24728800	4.91347100
H	-2.05557600	4.52540900	-3.84770300
H	-2.05275900	4.52852500	3.85921200
H	-5.64533900	7.36413900	3.85022300
H	-6.54818000	8.61424900	1.97456100
H	-6.54962100	8.61265700	-1.96306900
H	-5.64815100	7.36103100	-3.83837900
H	-7.73947600	4.41613900	-4.92004100
H	-9.94792800	4.29555000	-3.95709600
H	-9.94502700	4.29875600	3.97455900
H	-7.73587200	4.42011900	4.93579300
H	-4.46716500	1.58402400	-4.86855000
H	-8.02874100	0.79733800	-5.88258900
H	-10.37456300	-2.03219200	-4.89790800
H	-10.26130600	-1.93835500	-2.49739300

H	-4.46359800	1.58796500	4.88420100
H	-4.59190700	-2.22344900	5.90496200
H	-10.37097300	-2.02822500	4.92079700
H	-10.25947300	-1.93632900	2.52012600
H	-4.59621800	-2.22821200	-5.88614200
H	-8.02443000	0.80209800	5.90147400
H	-7.08286500	-4.67175900	4.86554500
H	-9.02220200	-6.09480000	1.85833800
H	-9.02355300	-6.09629100	-1.83315000
H	-7.08641900	-4.67568000	-4.84292400
H	-5.45234900	-9.18488900	-1.86366800
H	-5.45097600	-9.18337400	1.88874100
H	-2.76582900	-5.24884200	-4.91795500
H	-2.76222600	-5.24485800	4.93787900
H	-0.79749200	-9.63395200	-1.85140800
H	-0.79612800	-9.63244800	1.87343900
H	0.60643700	-7.96116600	-4.86655100
H	0.61000200	-7.95722700	4.88620000
H	0.74203700	-4.31714500	-5.88372800
H	0.74634600	-4.31238300	5.90033700
H	4.33325600	-7.15250600	-5.88741600
H	4.33756700	-7.14774000	5.90368600
H	3.27450600	-1.07220900	-7.44231700
H	3.27994900	-1.06618700	7.45444800
H	-5.28514100	-7.82961700	3.85622400
H	-5.28795500	-7.83272200	-3.83236700
Al	3.52093600	-0.44554700	1.52295200
Si	2.87151700	-2.41741500	-3.64220000
Si	3.53268500	-0.44915300	-1.49622100
Si	3.01503900	2.41622700	-2.27599700
Si	6.59190000	-0.57529300	-2.27286000
Si	3.00150900	2.40063200	2.27770200
Si	2.87615300	-2.39812900	3.62057100
Si	6.58783800	-0.56704100	2.28414700
O	2.99653800	-1.77168200	-2.21116400
O	2.83645600	0.83433100	-2.16666800
O	2.85633100	-0.55974900	-0.06836400
O	5.14193400	-0.33015700	-1.61428300
O	5.21110000	-0.25761400	1.59279700
O	2.92086100	-1.90393400	2.13875200
O	2.63100100	0.88259600	2.11161600
O	1.41874600	-2.98296500	3.85144400
O	1.41915300	-3.02374600	-3.74909600
O	-0.80854300	-4.04447000	3.96677900

O	-0.82229500	-4.02203400	-3.96775800
O	-3.00087100	-2.84834900	4.07235600
O	-4.15279700	-0.65342900	4.03395500
O	-4.45920400	1.18657500	2.34710100
O	-2.91029000	3.21519100	1.80385600
O	-0.49000500	3.93885500	2.13775700
O	1.86391400	3.21125000	1.47632400
O	1.79967500	3.12022900	-1.52024700
O	-0.49351500	3.92978800	-2.16202300
O	-2.90895800	3.21298600	-1.78297400
O	-3.01243600	-2.84848200	-4.05137400
O	-4.15772400	-0.65356100	-4.00995900
O	-4.45794800	1.19385300	-2.33313900
O	-4.50042800	2.07675800	0.01035900
O	0.53082000	4.99507800	-0.01348800
Si	0.67271200	-4.21443300	4.49076600
Si	-2.35755800	-4.29818000	3.94972700
Si	0.65814600	-4.22340300	-4.46491200
Si	-2.37187100	-4.29735700	-3.92923800
Si	-4.36725000	-2.15016900	-4.48869200
Si	-4.35822100	-2.15270000	4.50747800
Si	-4.87638200	0.74001900	3.82097700
Si	-4.88078800	0.74245600	-3.80178900
Si	-4.32649900	2.54074500	1.52503800
Si	-4.32846200	2.54296600	-1.50104000
Si	-2.00593200	4.36257800	-2.44207100
Si	-1.99656800	4.36409300	2.45184800
Si	0.86286800	4.41558400	-1.46389000
Si	0.87058900	4.43806900	1.45190600
H	1.69102400	-2.67493800	0.34038500
C	0.73488600	-2.30012700	-0.03256800
H	0.55428500	-2.67631500	-1.03910900
H	-0.01902000	-2.67170900	0.66690100
C	0.78358100	-0.82017000	0.01736400
H	0.73444500	-0.20592500	-0.86990700
H	0.84450100	-0.28910100	0.95820500
O	-1.31165500	-0.45050100	0.14117700
C	-2.15260200	-1.01239000	-0.89215300
H	-2.19487900	-0.26115900	-1.68161400
H	-1.67631400	-1.91365500	-1.29487100
C	-3.53735400	-1.32750400	-0.36643600
H	-4.02077800	-0.43389100	0.03309300
H	-4.15384600	-1.71383300	-1.18259400
H	-3.49649300	-2.09877900	0.41191200

H	-1.60752800	-0.79196900	0.99413600

TS13

O	-4.29940300	3.34872700	1.75937400
O	-3.24561200	2.50096000	3.94149400
O	-7.36094100	-1.87947400	1.87224500
O	-7.72410000	0.65802200	1.88405900
O	-6.70892700	-0.47266300	3.96144900
O	-4.22754300	-3.36294700	3.80801400
O	-3.12126300	-1.37864300	4.99685800
O	-1.24562100	-5.68151200	3.89490500
O	-4.21742400	-3.37968100	-3.78564200
O	-3.10752400	-1.40108300	-4.98026000
O	-1.23516400	-5.69882800	-3.85384600
Si	-1.44118700	-7.25582000	3.82534400
Si	-1.43078600	-7.27278800	-3.77769400
O	2.65117300	-5.00201600	2.45998900
O	-2.99084400	-7.61765400	3.92073700
O	-0.93826500	-7.80762700	2.41755100
O	2.65783300	-5.01291600	-2.41129100
O	-2.98022000	-7.63504800	-3.87573600
O	-0.93175300	-7.81830000	-2.36612800
Si	2.76097200	-6.30461800	1.55763500
Si	0.33954300	-8.24448800	1.56486800
Si	2.76513800	-6.31144600	-1.50284900
Si	0.34373200	-8.25134300	-1.50802400
O	1.56456600	-7.29011000	1.91040800
O	4.12656700	-7.04885600	1.79395900
O	2.68718900	-5.83020600	0.02617500
O	-0.08269000	-8.12995700	0.02753200
O	1.56969500	-7.29850900	-1.85447700
O	4.13137300	-7.05672200	-1.73212800
O	-4.29472900	3.34080800	-1.76685800
O	-3.23491800	2.48335200	-3.94227800
O	-7.35579400	-1.88779400	-1.86466800
O	-7.71895800	0.64960000	-1.88875500
O	-6.69814000	-0.49037900	-3.95833300
Si	-3.66221500	-0.47607400	-6.14236100
Si	-3.07430900	2.45999300	-5.51659100
Si	-6.71164100	-0.55288500	-5.52472900
O	-2.98718800	0.96503000	-6.04792200
O	-5.24288300	-0.34118700	-6.07202100
O	-7.26628800	-1.94973600	-6.03336700

Si	-3.08935200	2.48461700	5.51626800
Si	-6.72671300	-0.52819100	5.52793800
Si	-3.67903000	-0.44878200	6.15361500
O	-3.00368100	0.99201200	6.05452800
O	-7.28280600	-1.92277000	6.04128800
O	-5.25942300	-0.31411000	6.07825400
Si	-5.66497000	4.09281300	1.52294700
Si	-8.08637800	2.15297300	1.53017900
Si	-5.66078600	4.08602000	-1.53753200
Si	-8.08224800	2.14612300	-1.54277100
O	-6.86136100	3.10735200	1.87573000
O	-5.77474500	5.39544800	2.42529700
O	-5.73873400	4.56726300	-0.00851000
O	-8.50863800	2.26750000	-0.00715100
O	-6.85623200	3.09894900	-1.88915700
O	-5.76811600	5.38457300	-2.44597600
Si	-7.35592800	-3.42659600	1.54935700
Si	-7.35175300	-3.43348700	-1.53501300
O	-6.22084100	-4.17005900	2.37045100
O	-7.07417900	-3.66536400	0.00804700
O	-6.21436300	-4.18064000	-2.34961100
Si	-5.43084500	-4.37620400	3.73347300
O	-4.87745900	-5.86210700	3.77494400
O	-6.41386900	-4.14042100	4.95103200
Si	-7.28383800	-3.50446900	6.10651900
Si	-4.43501600	-7.22233900	4.45268900
Si	-5.42065400	-4.39287300	-3.70956900
O	-4.86721300	-5.87887800	-3.74296500
O	-6.40044100	-4.16242100	-4.93081900
Si	-7.26721900	-3.53168700	-6.09154000
Si	-4.42293800	-7.24211900	-4.41337800
O	5.67070400	-2.97783400	3.78288600
O	5.68095900	-2.99461600	-3.73498700
O	6.59953800	0.53821700	3.89171000
O	6.61009600	0.52092800	-3.85696600
O	5.61463200	3.61270800	1.89159800
O	5.61976000	3.60430600	-1.87328800
O	2.60333300	5.84031300	1.80292000
O	2.60823800	5.83226300	-1.80275900
O	-1.37369700	5.74917400	2.35491800
O	-1.36729200	5.73861000	-2.36519300
Si	-6.13082300	6.03525200	3.83819500
Si	-6.12033100	6.01807000	-3.86267700
Si	-1.41613500	6.57024800	3.71452400

Si	-1.40598100	6.55365100	-3.72853900
Si	6.82778700	4.57753300	1.53889900
Si	6.83195700	4.57070500	-1.52159100
Si	2.85203800	7.36950900	1.52385400
Si	2.85620100	7.36269500	-1.52984100
O	-5.35173600	7.42463600	3.96155800
O	-5.34090400	7.40689200	-3.99011300
O	-0.17580700	7.53766400	3.78925800
O	-2.75522400	7.41963000	3.74820700
O	-0.16546000	7.52072200	-3.80421600
O	-2.74497900	7.40285500	-3.76966400
Si	-3.99646200	8.13701900	4.41901900
Si	-3.98438000	8.11724000	-4.44705200
O	4.36365100	7.73546000	1.83289300
O	1.88592900	8.30643300	2.39934800
O	2.54286100	7.70762200	-0.00422900
O	4.36865300	7.72727300	-1.83639200
O	1.89248700	8.29569700	-2.41213600
Si	1.06326200	8.50213300	3.74759300
Si	1.07349400	8.48537300	-3.76347900
O	6.38189600	6.06863600	1.76776300
O	8.07640900	4.20385900	2.44683600
O	7.28160800	4.40088900	0.00960500
O	6.38670000	6.06076500	-1.75832500
O	8.08304600	4.19299100	-2.42444900
Si	5.82204100	7.41357600	2.38150200
Si	5.82852400	7.40295200	-2.37958300
Si	8.62394000	3.72864100	3.86344600
Si	8.63443700	3.71146000	-3.83742800
Si	8.06679500	0.75240900	4.44202800
Si	8.07884400	0.73267000	-4.40423900
O	8.62284100	2.14697000	3.92867500
O	8.63350000	2.12951300	-3.89560000
O	9.01713400	-0.38124600	3.82935200
O	9.02750400	-0.39823400	-3.78392100
Si	9.48925200	-1.89540800	3.78227400
Si	9.49949000	-1.91216600	-3.72880100
O	8.25012400	-2.85979600	3.82393900
O	8.26046600	-2.87673700	-3.76953800
Si	7.00978900	-3.82721800	3.74920700
Si	7.01993400	-3.84381900	-3.69386900
O	7.05223400	-4.64828700	2.38959100
O	7.05866400	-4.65881800	-2.33049400
Si	5.32437800	-7.87269400	2.41493100

Si	5.33086600	-7.88331500	-2.34615000
Si	7.54710900	-5.91616900	1.57519000
Si	7.55131200	-5.92305100	-1.50909900
O	6.70030800	-7.20360000	1.96891000
O	6.70558000	-7.21223200	-1.89937500
O	7.37687000	-5.59719200	0.03204000
H	-5.32215200	-8.32582400	-3.99160200
H	-5.33308600	-8.30791800	4.03330400
H	-8.68259700	-3.94132400	5.99021100
H	-8.68341600	-3.94417300	1.91097500
H	-8.67821800	-3.95267700	-1.89792900
H	-8.66629200	-3.96802300	-5.97709300
H	-6.75787900	-3.97649700	-7.39676400
H	-6.77805900	-3.94344700	7.41510200
H	-7.59912200	0.51260900	6.09043100
H	-7.58258500	0.48537300	-6.09423100
H	-9.26250800	2.55507300	2.31501800
H	-9.25616200	2.54470700	-2.33253600
H	-7.56809700	6.33881500	3.89285900
H	-7.55745100	6.32137900	-3.92260700
H	-5.70980000	5.10318400	-4.93754700
H	-4.07579300	9.51784200	3.92107400
H	-3.85133800	8.12177200	5.88175700
H	-4.06506100	9.50026700	-3.95549300
H	-3.83527700	8.09546500	-5.90930700
H	-5.72322700	5.12517600	4.91825500
H	-4.22536400	3.12350300	-6.14569500
H	-1.82643700	3.11342200	-5.93693700
H	-4.24207800	3.15096400	6.13926500
H	-1.84259100	3.13987000	5.93709400
H	-1.39473500	5.63608000	4.84932900
H	-1.38150900	5.61441800	-4.85910700
H	1.93088600	8.23226000	4.90314800
H	0.62591300	9.90488900	3.79121200
H	0.63627700	9.88792000	-3.81454900
H	1.94426300	8.21035600	-4.91544700
H	2.13466600	4.50829200	3.85587300
H	2.14517200	4.49109900	-3.85101700
H	5.77805700	7.27487900	-3.84312200
H	6.69551700	8.51685400	-1.96914900
H	6.69015200	8.52563900	1.96846900
H	5.76758100	7.29203200	3.84545500
H	7.81455500	4.32030000	4.93849600
H	10.02270300	4.16549300	3.97975600

H	10.03350900	4.14779700	-3.95187300
H	7.82798100	4.29831000	-4.91730600
H	4.50202200	1.53530700	4.88865500
H	8.05018800	0.70027900	5.91101200
H	10.35684500	-2.16521500	4.93786500
H	10.24908200	-2.07610100	2.53692100
H	4.51530900	1.51354600	-4.86406700
H	4.59066600	-2.30200300	-5.87462800
H	10.37022400	-2.18712100	-4.88080600
H	10.25591700	-2.08729700	-2.48058500
H	4.57459100	-2.27568800	5.91644100
H	8.06624200	0.67398500	-5.87301300
H	7.04441900	-4.78304400	-4.82443000
H	8.95794700	-6.22582300	-1.81010100
H	8.95291900	-6.21758700	1.88137600
H	7.03119300	-4.76138100	4.88401000
H	5.33770300	-9.25453000	1.91368500
H	5.34281600	-9.26290500	-1.83871000
H	2.70270300	-5.27229200	4.95293700
H	2.71615400	-5.29431900	-4.90284500
H	0.67690900	-9.63678200	1.89440800
H	0.68198000	-9.64509400	-1.83042900
H	-0.70805400	-7.93607000	4.90268900
H	-0.69476200	-7.95783100	-4.85002700
H	-0.79308600	-4.28782000	5.91011000
H	-0.77695100	-4.31421600	-5.87400400
H	-4.42463800	-7.07132100	5.91487500
H	-4.40857400	-7.09762800	-5.87618900
H	-3.28140700	-1.00275900	7.45572800
H	-3.26104500	-1.03596200	-7.44098000
H	5.19981800	-7.91210300	-3.81001900
H	5.18934200	-7.89495000	3.87854800
Al	-3.49097000	-0.48335700	-1.38917100
Si	-2.87507700	-2.34881900	3.67702800
Si	-3.55172800	-0.43283500	1.47271100
Si	-2.97550600	2.44897600	2.23617000
Si	-6.60067400	-0.47250700	2.30422300
Si	-2.96317500	2.40744700	-2.24211100
Si	-2.86599300	-2.37802200	-3.64456600
Si	-6.57626600	-0.47799900	-2.29305800
O	-2.71376600	-1.31947200	2.50399200
O	-2.88273900	1.02773500	1.52610100
O	-3.36468500	-1.22641800	0.13697700
O	-5.10589700	-0.23562200	1.84819700

O	-5.13264600	-0.23809000	-1.76158300
O	-2.69125900	-1.82130000	-2.16893100
O	-2.65479400	0.93416700	-1.79441100
O	-1.43997800	-3.00662500	-3.88543100
O	-1.49017800	-3.09247000	3.70645000
O	0.78577700	-4.06850900	-3.90344900
O	0.78540200	-4.00514800	4.00692400
O	2.98805000	-2.90018200	-4.08245900
O	4.18072000	-0.71498200	-4.03756700
O	4.52757600	1.11447300	-2.32485300
O	2.98600300	3.15283200	-1.81052600
O	0.58973700	3.92389700	-2.11116600
O	-1.77485600	3.23427600	-1.54452300
O	-1.70017100	3.19324300	1.65428800
O	0.61513600	3.93297000	2.11845500
O	3.00019800	3.15431500	1.83404100
O	2.99054300	-2.87850400	4.08629600
O	4.15277700	-0.69084300	4.07041800
O	4.52789300	1.10869200	2.34165400
O	4.54138400	2.01712900	0.00679400
O	-0.47268500	5.00405500	0.00961100
Si	-0.67010400	-4.23861500	-4.47978500
Si	2.33830300	-4.33618000	-3.91629900
Si	-0.69503000	-4.21406100	4.49344800
Si	2.33032200	-4.31331600	3.96229400
Si	4.35210300	-2.19462700	4.52004700
Si	4.36376100	-2.22104800	-4.48379000
Si	4.91825900	0.66541900	-3.80049900
Si	4.90552200	0.68038800	3.82830500
Si	4.39481000	2.46986100	-1.50907300
Si	4.40303400	2.46329700	1.52449700
Si	2.11773300	4.33593400	2.45319400
Si	2.09619300	4.32713200	-2.44340400
Si	-0.75352900	4.45005700	1.47892500
Si	-0.77789300	4.44723300	-1.45981900
C	-0.49298100	-2.64986500	0.27316000
H	-0.90443100	-2.16128200	1.14880200
H	0.57862800	-2.58038700	0.12861100
C	-1.29920000	-3.39735500	-0.55760600
H	-1.85700100	-2.51210000	-1.28606800
H	-0.81953000	-4.06214600	-1.27373900
H	-2.25305500	-3.71951300	-0.13600400
O	-0.16221600	-0.39965600	-0.47255000
C	0.19727100	-0.25461600	-1.84362700

H	-0.00312900	0.77706000	-2.15251200
H	-0.47600500	-0.89865200	-2.41025700
C	0.18482200	0.73841500	0.31712000
H	1.10922200	1.18463900	-0.07286100
C	1.63791200	-0.64758500	-2.08879900
H	1.80401600	-1.70290000	-1.85160700
H	1.88867200	-0.51286500	-3.14178500
H	2.33643100	-0.04914700	-1.49908200
H	-0.62008800	1.47229600	0.21705000
C	0.38721400	0.34325500	1.76444000
H	1.21724200	-0.36218200	1.87328700
H	0.62150200	1.24097800	2.34240100
H	-0.51715200	-0.10273200	2.18494300

TS15

O	-4.21729200	3.44049200	1.76802200
O	-3.17498100	2.57505000	3.94871600
O	-7.36600000	-1.73559100	1.88482300
O	-7.68650000	0.80764200	1.89718700
O	-6.68768900	-0.34002500	3.97319600
O	-4.25537300	-3.27154400	3.81638400
O	-3.11436500	-1.30614500	5.00381000
O	-1.31267500	-5.63983500	3.89925000
O	-4.25562600	-3.28807100	-3.77727900
O	-3.11426100	-1.32832200	-4.97331800
O	-1.31280500	-5.65694400	-3.84951000
Si	-1.53472900	-7.21063500	3.82990800
Si	-1.53471700	-7.22740200	-3.77313900
O	2.59306500	-5.02577100	2.45917300
O	-3.09011400	-7.54641300	3.92735000
O	-1.04301300	-7.77073800	2.42143100
O	2.59306800	-5.03654200	-2.41211200
O	-3.09014300	-7.56360000	-3.86913200
O	-1.04303700	-7.78128200	-2.36225300
Si	2.67978600	-6.32998800	1.55663900
Si	0.22614900	-8.22894200	1.56703800
Si	2.67977100	-6.33673400	-1.50384800
Si	0.22613800	-8.23571600	-1.50585700
O	1.46747600	-7.29527800	1.91097500
O	4.03301000	-7.09705400	1.79112700
O	2.61194100	-5.85432900	0.02529000
O	-0.19614500	-8.10726500	0.03026700
O	1.46746200	-7.30357600	-1.85391400

O	4.03299800	-7.10482600	-1.73496300
O	-4.21743700	3.43267100	-1.75821300
O	-3.17505800	2.55765300	-3.93506300
O	-7.36595800	-1.74381200	-1.85209300
O	-7.68651300	0.79932100	-1.87563100
O	-6.68772400	-0.35753000	-3.94659400
Si	-3.65489100	-0.39407600	-6.13465500
Si	-3.01695700	2.53167900	-5.50958900
Si	-6.70435300	-0.41972300	-5.51297200
O	-2.95564600	1.03548900	-6.04107500
O	-5.23297800	-0.23268100	-6.06221100
O	-7.28304400	-1.80704300	-6.02091100
Si	-3.01692500	2.55600900	5.52328200
Si	-6.70432300	-0.39532200	5.53970500
Si	-3.65490800	-0.36711000	6.16133300
O	-2.95560300	1.06215100	6.06138600
O	-7.28306300	-1.78039700	6.05375800
O	-5.23291700	-0.20592700	6.08807600
Si	-5.57049100	4.20740500	1.53343100
Si	-8.02410500	2.30848200	1.54383000
Si	-5.57048900	4.20069300	-1.52705200
Si	-8.02417400	2.30171400	-1.52912300
O	-6.78278300	3.24214700	1.88777800
O	-5.65718700	5.51165600	2.43596000
O	-5.63831600	4.68310100	0.00208500
O	-8.44642500	2.43015600	0.00706500
O	-6.78280000	3.23384500	-1.87711300
O	-5.65721400	5.50091100	-2.43531800
Si	-7.38738500	-3.28256400	1.56188700
Si	-7.38742300	-3.28937200	-1.52248700
O	-6.26384600	-4.04501400	2.38145100
O	-7.11173200	-3.52595100	0.02019700
O	-6.26381700	-4.05547000	-2.33861500
Si	-5.47561200	-4.26445700	3.74341600
O	-4.94718800	-5.75944000	3.78411000
O	-6.45292000	-4.01226600	4.96228800
Si	-7.31055600	-3.36185900	6.11894700
Si	-4.52674000	-7.12694100	4.46123200
Si	-5.47559200	-4.28092800	-3.69963400
O	-4.94721500	-5.77601100	-3.73380500
O	-6.45299400	-4.03400400	-4.91957400
Si	-7.31060300	-3.38875200	-6.07912400
Si	-4.52677600	-7.14648500	-4.40484400
O	5.64790200	-3.05262000	3.77810900

O	5.64788400	-3.06920300	-3.73977200
O	6.63576400	0.44733900	3.88579100
O	6.63573400	0.43025600	-3.86289200
O	5.69994400	3.53802700	1.88707400
O	5.69992800	3.52972500	-1.87781700
O	2.72634300	5.81586600	1.80245900
O	2.72632200	5.80791200	-1.80322300
O	-1.25092000	5.79146500	2.35974100
O	-1.25096400	5.78102700	-2.36037500
Si	-6.00059800	6.15727600	3.84934900
Si	-6.00062800	6.14030000	-3.85153200
Si	-1.27776200	6.61306900	3.71942300
Si	-1.27777900	6.59667000	-3.72364700
Si	6.92865300	4.48237200	1.53278800
Si	6.92864100	4.47562400	-1.52770400
Si	3.00030900	7.34068700	1.52310300
Si	3.00030100	7.33395300	-1.53059500
O	-5.19813800	7.53338100	3.97171200
O	-5.19817000	7.51585000	-3.97996700
O	-0.02127300	7.55952800	3.79253500
O	-2.60236100	7.48480500	3.75490900
O	-0.02130200	7.54278700	-3.80094700
O	-2.60238800	7.46823200	-3.76296800
Si	-3.83049000	8.22289500	4.42739100
Si	-3.83052300	8.20335100	-4.43869000
O	4.51826100	7.68119900	1.83014200
O	2.05122800	8.29365100	2.39990600
O	2.69482100	7.68401600	-0.00455800
O	4.51825100	7.67310900	-1.83914600
O	2.05121200	8.28304300	-2.41158300
Si	1.23375300	8.50306500	3.74924900
Si	1.23372300	8.48650500	-3.76183100
O	6.50815600	5.98073700	1.76228500
O	8.17203300	4.08774600	2.43905500
O	7.37741300	4.29821000	0.00288700
O	6.50814200	5.97296100	-1.76380700
O	8.17201300	4.07700800	-2.43223400
Si	5.97177000	7.33485600	2.37680300
Si	5.97174900	7.32435800	-2.38428600
Si	8.71339200	3.60333600	3.85492300
Si	8.71336500	3.58636000	-3.84595900
Si	8.10713800	0.63684600	4.43416400
Si	8.10710200	0.61734200	-4.41211100
O	8.68583000	2.02190300	3.92011000

O	8.68580000	2.00465400	-3.90417200
O	9.03750100	-0.51257000	3.82019500
O	9.03747000	-0.52935500	-3.79308500
Si	9.48407600	-2.03444100	3.77244900
Si	9.48405000	-2.05099900	-3.73863300
O	8.22899100	-2.97789600	3.81573700
O	8.22895900	-2.99463600	-3.77774800
Si	6.97249600	-3.92436000	3.74262600
Si	6.97247100	-3.94076300	-3.70045700
O	6.99934600	-4.74595700	2.38293300
O	6.99932700	-4.75636400	-2.33715600
Si	5.21764800	-7.94091000	2.41048400
Si	5.21763000	-7.95140500	-2.35060200
Si	7.47178800	-6.02192700	1.56784200
Si	7.47177600	-6.02872800	-1.51645000
O	6.60402100	-7.29498300	1.96265300
O	6.60400700	-7.30351400	-1.90563600
O	7.30487600	-5.70006300	0.02492800
H	-5.44349100	-8.21496500	-3.98190200
H	-5.44346100	-8.19727200	4.04301200
H	-8.71660400	-3.77516900	6.00448700
H	-8.72289100	-3.77780200	1.92525600
H	-8.72289700	-3.78620600	-1.88365300
H	-8.71665100	-3.80154900	-5.96282900
H	-6.81053700	-3.84198500	-7.38503700
H	-6.81047800	-3.80932900	7.42684500
H	-7.55839200	0.65994600	6.10338500
H	-7.55850400	0.63303400	-6.08128900
H	-9.19227600	2.73022800	2.33024300
H	-9.19228100	2.71998500	-2.31731700
H	-7.43250200	6.48491800	3.90593000
H	-7.43253300	6.46768900	-3.90954300
H	-5.60694000	5.21870500	-4.92697100
H	-3.88729400	9.60487900	3.92958800
H	-3.68370000	8.20514200	5.88993400
H	-3.88732500	9.58751400	-3.94698600
H	-3.68374900	8.17914700	-5.90114200
H	-5.60690000	5.24043400	4.92884100
H	-4.15754800	3.21444600	-6.13714400
H	-1.75885300	3.16409200	-5.93157500
H	-4.15747400	3.24158000	6.14782800
H	-1.75878200	3.19022400	5.94246700
H	-1.27053900	5.67861800	4.85417500
H	-1.27057800	5.65721400	-4.85427100

H	2.09825900	8.21861100	4.90364200
H	0.82007000	9.91296200	3.79348700
H	0.82004100	9.89619400	-3.81228200
H	2.09822000	8.19696600	-4.91496300
H	2.23811400	4.49179800	3.85599700
H	2.23808800	4.47481000	-3.85090100
H	5.91719400	7.19722100	-3.84776000
H	6.85786100	8.42352900	-1.97497600
H	6.85787600	8.43221000	1.96264700
H	5.91722300	7.21417000	3.84082500
H	7.91548000	4.20844500	4.93106400
H	10.11944300	4.01664300	3.96938500
H	10.11941100	3.99915800	-3.96225100
H	7.91544000	4.18671700	-4.92474800
H	4.55660700	1.47944600	4.88555200
H	8.09161200	0.58492900	5.90316800
H	10.34855300	-2.31882900	4.92687900
H	10.23911000	-2.22780000	2.52608100
H	4.55656700	1.45794500	-4.86718100
H	4.56652900	-2.35828200	-5.87794300
H	10.34851400	-2.34047400	-4.89180200
H	10.23909000	-2.23886300	-2.49142900
H	4.56656400	-2.33228100	5.91313800
H	8.09156300	0.55894900	-5.88086800
H	6.97968500	-4.88021000	-4.83107400
H	8.87273000	-6.35505400	-1.81933100
H	8.87274600	-6.34691600	1.87214900
H	6.97972400	-4.85880700	4.87737500
H	5.20711000	-9.32275100	1.90918400
H	5.20709500	-9.33102600	-1.84321500
H	2.64336500	-5.29699600	4.95204300
H	2.64334800	-5.31876300	-4.90374900
H	0.54053600	-9.62671900	1.89609200
H	0.54051800	-9.63493300	-1.82874800
H	-0.81168800	-7.90314800	4.90625700
H	-0.81172100	-7.92465000	-4.84646900
H	-0.83413300	-4.25403400	5.91388800
H	-0.83410200	-4.28011800	-5.87023700
H	-4.51188500	-6.97618900	5.92340600
H	-4.51193100	-7.00218400	-5.86766900
H	-3.26490900	-0.92774700	7.46290100
H	-3.26490100	-0.96055600	-7.43382300
H	5.08417200	-7.97791900	-3.81429500
H	5.08420200	-7.96096900	3.87427900

Al	-3.46021400	-0.33602100	-1.43952000
Si	-2.87752300	-2.29518100	3.69060600
Si	-3.53957500	-0.37102500	1.46711000
Si	-2.90156400	2.51818000	2.24367000
Si	-6.58332700	-0.34122500	2.31670100
Si	-2.87689800	2.52363600	-2.23333800
Si	-2.88645400	-2.30807600	-3.63425100
Si	-6.56855900	-0.34753500	-2.28888500
O	-2.76112700	-1.39970500	2.40728400
O	-2.81391600	1.05117500	1.62132300
O	-3.37144200	-1.10179500	0.08165800
O	-5.09366300	-0.14489000	1.82287200
O	-5.10093400	-0.14860900	-1.80629700
O	-2.54499100	-1.56624000	-2.26306500
O	-2.57223600	1.06780800	-1.72560800
O	-1.49724000	-2.99799400	-3.88710600
O	-1.48748400	-3.00765300	3.77654500
O	0.74123900	-4.02481100	-3.90919800
O	0.75665400	-4.00823400	4.02870200
O	2.96042800	-2.92493500	-4.06204300
O	4.17114700	-0.76149200	-4.03382700
O	4.55642900	1.05246600	-2.32628600
O	3.07010000	3.12669200	-1.80910000
O	0.68462700	3.93803100	-2.10792600
O	-1.70313700	3.34857400	-1.52798700
O	-1.63677100	3.24059700	1.61059900
O	0.69009900	3.93759700	2.15860900
O	3.07291100	3.14006800	1.80582400
O	2.97336200	-2.90732300	4.08352400
O	4.17481400	-0.73989200	4.06881700
O	4.55652800	1.06499400	2.34596700
O	4.61549200	1.95691100	0.00579200
O	-0.31782200	5.04737000	0.03061800
Si	-0.71823600	-4.21976800	-4.47707600
Si	2.28558000	-4.35236700	-3.91560100
Si	-0.72564200	-4.18612900	4.50265000
Si	2.29254600	-4.33270100	3.96039700
Si	4.34408100	-2.24714600	4.51737400
Si	4.34291000	-2.26841700	-4.48290700
Si	4.94393200	0.60216700	-3.80228000
Si	4.94384800	0.62043900	3.82461400
Si	4.46203300	2.41044000	-1.50884300
Si	4.46409300	2.41608600	1.51893800
Si	2.20863600	4.32031100	2.45319900

Si	2.19825600	4.31033800	-2.44340100
Si	-0.65246300	4.47837700	1.48598500
Si	-0.65225100	4.51018600	-1.43876200
C	0.50849600	-1.98435500	-0.98107600
H	0.79714600	-2.99140700	-1.26664900
H	0.72338100	-1.70113100	0.04142600
C	0.01716100	-1.09527000	-1.90696500
H	-1.24478400	-1.46610000	-1.95865700
H	-0.06589900	-0.04421200	-1.64205200
H	0.20003800	-1.33409500	-2.95375700
O	-1.05263500	-3.13363100	0.34125800
H	-1.47120800	-2.46867200	0.90198500
H	-1.81554300	-3.58554700	-0.06879000
O	-3.51642800	-3.80290700	-0.58823600
H	-3.76954100	-2.91450500	-0.29229700
H	-4.21162100	-4.40912600	-0.31776200

TS16

O	-4.31592600	3.31856600	1.77992700
O	-3.25879900	2.47134200	3.96064400
O	-7.35416200	-1.92342900	1.88220400
O	-7.72862500	0.61239600	1.89877900
O	-6.70883500	-0.51771400	3.97421900
O	-4.21456700	-3.39661700	3.81578600
O	-3.11737400	-1.40967400	5.00863700
O	-1.22236300	-5.70203400	3.89887200
O	-4.20287600	-3.39882500	-3.77788600
O	-3.10156800	-1.41302700	-4.96850200
O	-1.21030000	-5.70452900	-3.84989600
Si	-1.41089700	-7.27706300	3.82627200
Si	-1.39892200	-7.27948700	-3.77678400
O	2.67164800	-5.00245000	2.46605500
O	-2.95894700	-7.64597800	3.92065500
O	-0.90524600	-7.82393900	2.41753200
O	2.67931700	-5.00403200	-2.40523500
O	-2.94670700	-7.64845800	-3.87583500
O	-0.89774400	-7.82546000	-2.36615800
Si	2.78742800	-6.30282700	1.56124300
Si	0.37466500	-8.25347600	1.56428000
Si	2.79222900	-6.30380100	-1.49924700
Si	0.37948800	-8.25445200	-1.50861800
O	1.59535500	-7.29431100	1.91189100
O	4.15627900	-7.04142400	1.79642900

O	2.71183300	-5.82582900	0.03067400
O	-0.04777300	-8.13789500	0.02707800
O	1.60126500	-7.29550800	-1.85300300
O	4.16181500	-7.04254400	-1.72966600
O	-4.31052200	3.31739200	-1.74631300
O	-3.24647000	2.46881500	-3.92314500
O	-7.34824000	-1.92460000	-1.85471600
O	-7.72270100	0.61119200	-1.87404300
O	-6.69640600	-0.52028000	-3.94558000
Si	-3.66014400	-0.48828400	-6.12895200
Si	-3.08545000	2.44917400	-5.49746700
Si	-6.70931900	-0.57985800	-5.51209400
O	-2.99156300	0.95562900	-6.03162800
O	-5.24141200	-0.36057500	-6.05868000
O	-7.25763900	-1.97819500	-6.02350900
Si	-3.10277800	2.45269300	5.53541700
Si	-6.72668200	-0.57630600	5.54059500
Si	-3.67950600	-0.48451300	6.16705000
O	-3.01056400	0.95946200	6.07084700
O	-7.27665700	-1.97432500	6.05117300
O	-5.26046900	-0.35674000	6.09162100
Si	-5.68474900	4.05700900	1.54463800
Si	-8.09749000	2.10639200	1.54767400
Si	-5.67993100	4.05607100	-1.51584800
Si	-8.09272400	2.10541900	-1.52528200
O	-6.87680500	3.06555700	1.89529600
O	-5.80050400	5.35742100	2.44944500
O	-5.76032300	4.53404500	0.01407200
O	-8.51995300	2.22196800	0.01047800
O	-6.87089700	3.06435700	-1.86959900
O	-5.79286600	5.35586400	-2.42183800
Si	-7.34219300	-3.46989600	1.55636900
Si	-7.33737800	-3.47088700	-1.52800800
O	-6.20396600	-4.20985800	2.37627700
O	-7.05907900	-3.70446600	0.01466400
O	-6.19650900	-4.21140800	-2.34379300
Si	-5.41333000	-4.41508000	3.73906700
O	-4.85333600	-5.89857700	3.77781800
O	-6.39763300	-4.18600000	4.95687100
Si	-7.27065400	-3.55613500	6.11338900
Si	-4.40497100	-7.25811600	4.45306300
Si	-5.40159600	-4.41751100	-3.70399200
O	-4.84153300	-5.90096800	-3.74010500
O	-6.38216000	-4.18909900	-4.92500200

Si	-7.25150800	-3.56001900	-6.08469600
Si	-4.39105600	-7.26093700	-4.41302400
O	5.68187100	-2.96736300	3.79342900
O	5.69368300	-2.96976400	-3.72446100
O	6.59500800	0.55257800	3.90914300
O	6.60717100	0.55011100	-3.83954900
O	5.59680800	3.62645800	1.91469300
O	5.60271600	3.62525800	-1.85020200
O	2.57563100	5.84079100	1.82964200
O	2.58128400	5.83963900	-1.77604500
O	-1.40106200	5.73088300	2.38064700
O	-1.39367800	5.72934800	-2.33947500
Si	-6.15970800	5.99293600	3.86348900
Si	-6.14762000	5.99048600	-3.83740100
Si	-1.44742500	6.54916500	3.74180600
Si	-1.43573000	6.54680800	-3.70127300
Si	6.80572300	4.59735100	1.56408300
Si	6.81052600	4.59637600	-1.49641200
Si	2.81757500	7.37161000	1.55354100
Si	2.82237100	7.37063600	-1.50016100
O	-5.38684500	7.38553900	3.98965900
O	-5.37436400	7.38300600	-3.96203000
O	-0.21143500	7.52195500	3.81863900
O	-2.79029200	7.39250800	3.77683200
O	-0.19951600	7.51953700	-3.77485100
O	-2.77849100	7.39011500	-3.74105400
Si	-4.03484600	8.10308100	4.44875600
Si	-4.02092900	8.10026000	-4.41733500
O	4.32748200	7.74370200	1.86358800
O	1.84713100	8.30254900	2.43062100
O	2.50719700	7.71125500	0.02604300
O	4.33324500	7.74253300	-1.80570400
O	1.85468500	8.30101700	-2.38087400
Si	1.02333300	8.49201000	3.77906700
Si	1.03512100	8.48961900	-3.73202100
O	6.35314600	6.08601400	1.79569800
O	8.05581800	4.22751100	2.47156300
O	7.26062800	4.42564600	0.03454900
O	6.35868000	6.08488800	-1.73039900
O	8.06346400	4.22596100	-2.39973100
Si	5.78718300	7.42727400	2.41188300
Si	5.79465400	7.42575700	-2.34921200
Si	8.60518200	3.75203600	3.88737800
Si	8.61727200	3.74958600	-3.81351300

Si	8.06118600	0.77225400	4.46017100
Si	8.07507000	0.76943600	-4.38611500
O	8.61111500	2.17025500	3.94959100
O	8.62339600	2.16776500	-3.87470000
O	9.01668800	-0.35598600	3.84553100
O	9.02863700	-0.35841000	-3.76775800
Si	9.49555800	-1.86793600	3.79566400
Si	9.50735100	-1.87032700	-3.71542600
O	8.26073000	-2.83791300	3.83523800
O	8.27264600	-2.84032900	-3.75825500
Si	7.02473300	-3.81070600	3.75840600
Si	7.03642000	-3.81307000	-3.68468600
O	7.07110300	-4.62898300	2.39723600
O	7.07851100	-4.63048600	-2.32285800
Si	5.35762700	-7.86109900	2.41607600
Si	5.36510000	-7.86261300	-2.34501600
Si	7.57178400	-5.89309300	1.58052400
Si	7.57662500	-5.89407500	-1.50377300
O	6.73065000	-7.18503200	1.97161500
O	6.73672200	-7.18626600	-1.89667900
O	7.40042900	-5.57193700	0.03794900
H	-5.28551600	-8.34943800	-3.99349900
H	-5.29811300	-8.34688300	4.03142400
H	-8.66743000	-3.99899400	5.99596100
H	-8.66743300	-3.99406800	1.91672700
H	-8.66144500	-3.99528600	-1.89218600
H	-8.64864700	-4.00280100	-5.97136900
H	-6.73993500	-4.00006700	-7.39066200
H	-6.76318300	-3.99535100	7.42123700
H	-7.60383000	0.45952100	6.10489200
H	-7.58477000	0.45559200	-6.07979700
H	-9.27555400	2.50175100	2.33303700
H	-9.26824600	2.50027600	-2.31452800
H	-7.59833200	6.28998800	3.91843300
H	-7.58606600	6.28750100	-3.89704800
H	-5.73280400	5.07948900	-4.91392800
H	-4.12023000	9.48448300	3.95342600
H	-3.88994600	8.08569000	5.91149100
H	-4.10786700	9.48197500	-3.92315700
H	-3.87144100	8.08193700	-5.87959800
H	-5.74827300	5.08262600	4.94189500
H	-4.23932600	3.10874800	-6.12554100
H	-1.84041900	3.10895600	-5.91630900
H	-4.25858300	3.11270900	6.15944500

H	-1.85903100	3.11269100	5.95774600
H	-1.42208800	5.61294000	4.87483400
H	-1.40685000	5.60984900	-4.83362300
H	1.89192300	8.22380400	4.93428400
H	0.57973100	9.89271900	3.82527000
H	0.59166900	9.89029800	-3.78050700
H	1.90733200	8.22067900	-4.88433200
H	2.11250000	4.50278400	3.87995600
H	2.12460200	4.50033300	-3.82695200
H	5.74504500	7.30024900	-3.81300300
H	6.65659500	8.54272400	-1.93647800
H	6.65041400	8.54397700	2.00114900
H	5.73297600	7.30269600	3.87559100
H	7.79295500	4.33803300	4.96338700
H	10.00196100	4.19489300	4.00480800
H	10.01440900	4.19236900	-3.92683800
H	7.80842400	4.33489600	-4.89243600
H	4.49287500	1.53841100	4.90755700
H	8.04452400	0.71724900	5.92905000
H	10.36411600	-2.13607900	4.95091800
H	10.25643100	-2.04286800	2.55012500
H	4.50818100	1.53530600	-4.84518700
H	4.60073700	-2.27793700	-5.86300200
H	10.37952800	-2.13920300	-4.86777400
H	10.26430600	-2.04446600	-2.46739100
H	4.58221900	-2.27417600	5.92809200
H	8.06301700	0.71349600	-5.85500000
H	7.06531200	-4.75001800	-4.81702900
H	8.98465500	-6.19000600	-1.80506200
H	8.97886300	-6.18883000	1.88642200
H	7.05007500	-4.74692700	4.89143100
H	5.37720500	-9.24190400	1.91220000
H	5.38309500	-9.24310000	-1.84020300
H	2.72389100	-5.27724700	4.95849500
H	2.73938400	-5.28042200	-4.89731000
H	0.71816500	-9.64487900	1.89123500
H	0.72400800	-9.64606600	-1.83360900
H	-0.67495400	-7.95609200	4.90246800
H	-0.65964200	-7.95919900	-4.85027000
H	-0.77643900	-4.31018500	5.91682200
H	-0.75786200	-4.31403900	-5.86731700
H	-4.39555300	-7.10984100	5.91553500
H	-4.37704700	-7.11359400	-5.87555300
H	-3.27967500	-1.03919500	7.46818600

H	-3.25622800	-1.04390300	-7.42855400
H	5.23446900	-7.88919500	-3.80896200
H	5.22240100	-7.88674700	3.87962000
Al	-3.48943000	-0.49454700	-1.40268500
Si	-2.86380200	-2.38856700	3.69589900
Si	-3.55835700	-0.45533400	1.47334500
Si	-2.98456800	2.43002300	2.25611300
Si	-6.60010700	-0.51505900	2.31042800
Si	-2.96860900	2.40267300	-2.21750000
Si	-2.85145200	-2.38516900	-3.62688100
Si	-6.57524900	-0.51229600	-2.27784200
O	-2.77189500	-1.34807700	2.52888600
O	-2.86916800	0.99431000	1.57959000
O	-3.24092200	-1.21708600	0.13296400
O	-5.12894900	-0.27334300	1.76974900
O	-5.14355300	-0.27150800	-1.70871200
O	-2.59708600	-1.68870300	-2.24774200
O	-2.65170800	0.95150300	-1.71033400
O	-1.43075900	-3.01851800	-3.89467500
O	-1.45757100	-3.07932000	3.74612700
O	0.81068500	-4.04059700	-3.93239800
O	0.80866000	-4.00908200	4.04141200
O	3.00500000	-2.88575000	-4.05413000
O	4.16079600	-0.69486600	-4.01278000
O	4.50117400	1.12471900	-2.30824500
O	2.97483400	3.16163000	-1.78987700
O	0.57963200	3.92870400	-2.07799400
O	-1.78170500	3.24002000	-1.53215200
O	-1.71395100	3.16751000	1.64795100
O	0.59042200	3.92342200	2.15308900
O	2.98257500	3.16462200	1.84116000
O	3.00440200	-2.87908100	4.08071200
O	4.15443500	-0.68846600	4.08341000
O	4.50985300	1.12202000	2.36416700
O	4.53329300	2.02435700	0.02695000
O	-0.48359000	5.00426800	0.04040700
Si	-0.65403500	-4.24632800	-4.47596800
Si	2.35702000	-4.32853800	-3.90774600
Si	-0.67715900	-4.23225200	4.49980000
Si	2.34813200	-4.32037400	3.96814000
Si	4.35907000	-2.19423700	4.53081300
Si	4.37183900	-2.19931600	-4.46877900
Si	4.91171800	0.68287800	-3.78300300
Si	4.90103900	0.68694500	3.84695100

Si	4.38515000	2.48171100	-1.48824000
Si	4.38936400	2.47637600	1.54477600
Si	2.09361300	4.33443400	2.47656400
Si	2.08193000	4.33504700	-2.41963900
Si	-0.77429600	4.43614900	1.50322000
Si	-0.78977200	4.45239500	-1.43010100
C	0.22422300	-0.76666100	1.36785700
H	1.03238200	-1.44915000	1.61162700
H	-0.65458500	-0.83913700	1.99615900
C	0.37812600	0.24060800	0.42955500
H	0.09130300	-0.46635800	-0.60864900
H	-0.36208100	1.03802100	0.41479600
H	1.39778700	0.50735100	0.14829400
O	-0.79114000	-2.58626700	0.47976400
H	-0.41538100	-2.74213100	-0.39521000
H	-1.70454300	-2.28024700	0.31457000
O	-0.01389000	-1.17934100	-1.75221700
H	-0.95365100	-1.29021900	-2.05303500
C	0.83094700	-0.66855700	-2.80221100
H	1.81991400	-0.55222500	-2.35179100
H	0.90376200	-1.44404000	-3.56537400
C	0.31394700	0.63968200	-3.36394500
H	0.23858700	1.40886100	-2.59209800
H	0.99673100	0.99518100	-4.14210300
H	-0.67394600	0.50435900	-3.81359900
